# Supplementary material for: Sensitivity analysis for mistakenly adjusting for mediators in estimating total effect in observational studies
Source: BMJ Open. 2017 Nov 20;7(11):e015640. doi: 10.1136/bmjopen-2016-015640 (PMC5719285; doi:10.1136/bmjopen-2016-015640)
Supplement: Supplementary Appendix 1 [file bmjopen-2016-015640supp001.pdf]

Appendix:

The effect of adjusting for mediator was biased for estimating the total effect of exposure on outcome using logistic regression model. Theoretical derivation of Figure 1a as follow：

Suppose the logistic models among *E*, *M* and *D* are:

$$\text{logit}\{P(D=1 \mid e,m)\} = \alpha_1 + \beta_0 e + \beta_2 m,$$

$$\text{logit}\{P(M=1 \mid e)\} = \alpha_0 + \beta_1 e.$$

The total effect ($\beta_{E\to D}^{TE}$) of exposure *E* on outcome *D* on the odds ratio ($OR_{E\to D}^{TE}$) scale was equal to

$$\begin{aligned}
\beta_{E\to D}^{TE} &= \log(OR_{E\to D}^{TE}) \\
&= \log\left\{\frac{P(D_e=1)/\{1-P(D_e=1)\}}{P(D_{e^*}=1)/\{1-P(D_{e^*}=1)\}}\right\} \\
&= \log\left\{\frac{P(D_e=1)\times\{1-P(D_{e^*}=1)\}}{\{1-P(D_e=1)\}\times P(D_{e^*}=1)}\right\} \\
&= \log\left\{\frac{P(D=1 \mid e=1)\times P(D=0 \mid e^*=0)}{P(D=0 \mid e=1)\times P(D=1 \mid e^*=0)}\right\} \\
&= \log\left\{\frac{[\sum_m P(D=1 \mid e=1,m)P(m \mid e=1)]\times[\sum_m P(D=0 \mid e^*=0,m)P(m \mid e^*=0)]}{[\sum_m P(D=0 \mid e=1,m)P(m \mid e=1)]\times[\sum_m P(D=1 \mid e^*=0,m)P(m \mid e^*=0)]}\right\}
\end{aligned}$$

The effect ($\beta_{ED|M}(m)$) of adjusting for mediator *M* by logistic regression model is given

$$\begin{aligned}
\beta_{ED|M}(m) &= \text{logit}\{P(D=1 \mid e=1,m)\} - \text{logit}\{P(D=1 \mid e^*=0,m)\} \\
&= \log\left\{\frac{P(D=1 \mid e=1,m)\times P(D=0 \mid e^*=0,m)}{P(D=0 \mid e=1,m)\times P(D=1 \mid e^*=0,m)}\right\} \\
&= \beta_0
\end{aligned}$$

Therefore,

$$
\begin{aligned}
bias &= \beta_0 - \log(OR_{E\to D}^{TE}) \\
&= \log\left\{ \frac{\exp(\beta_0)}{\exp(\beta_0)\dfrac{\exp(\beta_2)\times A_1 + \exp(\beta_2)\times B_1 + C_1 + D_1}{\exp(\beta_2)\times A_1 + B_1 + \exp(\beta_2)\times C_1 + D_1}} \right\} \\
&= \log\left\{ \frac{\exp(\beta_2)\times A_1 + B_1 + \exp(\beta_2)\times C_1 + D_1}{\exp(\beta_2)\times A_1 + \exp(\beta_2)\times B_1 + C_1 + D_1} \right\}
\end{aligned}
$$

where

$$
\begin{aligned}
A_1 &= \exp(\beta_1 + \alpha_0)\times(1+\exp(\beta_0+\alpha_1))\times\exp(\alpha_0)\times(1+\exp(\alpha_1)) \\
B_1 &= \exp(\beta_1 + \alpha_0)\times(1+\exp(\beta_0+\alpha_1))\times(1+\exp(\beta_2+\alpha_1)) \\
C_1 &= (1+\exp(\beta_0+\beta_2+\alpha_1))\times\exp(\alpha_0)\times(1+\exp(\alpha_1)) \\
D_1 &= (1+\exp(\beta_0+\beta_2+\alpha_1))\times(1+\exp(\beta_2+\alpha_1))
\end{aligned}
$$

Focusing on the difference of between $\exp(\beta_2)\times B_1 + C_1$ and $B_1 + \exp(\beta_2)\times C_1$.

$$
\begin{aligned}
T(\beta_1) &= \exp(\beta_2)\times B_1 + C_1 - (B_1 + \exp(\beta_2)\times C_1) \\
&= \exp(\beta_2)\times(B_1 - C_1) - (B_1 - C_1) \\
&= (\exp(\beta_2)-1)\times(B_1 - C_1) \\
&= (\exp(\beta_2)-1)\times(\exp(\beta_1+\alpha_0)\times(1+\exp(\beta_0+\alpha_1))\times(1+\exp(\beta_2+\alpha_1)) \\
&\quad -(1+\exp(\beta_0+\beta_2+\alpha_1))\times\exp(\alpha_0)\times(1+\exp(\alpha_1))) \\
&= (\exp(\beta_2)-1)\times\exp(\alpha_0)\times[\exp(\beta_1)\times(1+\exp(\beta_0+\alpha_1)\times(1+\exp(\beta_2+\alpha_1)) \\
&\quad -(1+\exp(\beta_0+\beta_2+\alpha_1))\times(1+\exp(\alpha_1))]
\end{aligned}
$$

Then, detailed dissection:

1: $\beta_2 = 0$, $bias = 0$.

2: $\beta_2 > 0$,

① $\beta_1 = 0$: (i) $\beta_0 = 0$, $bias = 0$; (ii) $\beta_0 > 0$, $bias > 0$; (iii) $\beta_0 < 0$, $bias < 0$.

② $\beta_1 < 0$: (i) $\beta_0 = 0$, $bias > 0$; (ii) $\beta_0 > 0$, $bias > 0$; (iii) $\beta_0 < 0$, $bias > 0$.

proof (iii)

$$
\begin{aligned}
T(\beta_1) &= (\exp(\beta_2)-1)\times\exp(\alpha_0) \\
&\quad \times\{\exp(\beta_1)\times[1+\exp(\beta_0+\alpha_1)+\exp(\beta_2+\alpha_1)+\exp(\beta_0+\beta_2+2\alpha_1)] \\
&\quad -[1+\exp(\beta_0+\beta_2+\alpha_1)+\exp(\alpha_1)+\exp(\beta_0+\beta_2+2\alpha_1)]\}
\end{aligned}
$$

when $\beta_0 < 0$ and $\beta_2 > 0 \Rightarrow \exp(\beta_0) - 1 < 0 \qquad \exp(\beta_2) - 1 > 0$

According to $(a-1)(b-1) = ab - a - b + 1$, when $(a-1)(b-1) < 0 \Rightarrow ab + 1 < a + b$

$$
\begin{aligned}
&1+\exp(\beta_0+\beta_2+\alpha_1)+\exp(\alpha_1)+\exp(\beta_0+\beta_2+2\alpha_1)\\
&<1+\exp(\beta_0+\alpha_1)+\exp(\beta_2+\alpha_1)+\exp(\beta_0+\beta_2+2\alpha_1)\\
&\Rightarrow \exp(\beta_0+\beta_2)+1<\exp(\beta_0)+\exp(\beta_2)
\end{aligned}
$$

when

$$\beta_1<\log\left\{\frac{\exp(\beta_0+\beta_2)+1}{\exp(\beta_0)+\exp(\beta_2)}\right\}<0$$

$$\beta_1<\log\left\{\frac{1+\exp(\beta_0+\beta_2+\alpha_1)+\exp(\alpha_1)+\exp(\beta_0+\beta_2+2\alpha_1)}{1+\exp(\beta_0+\alpha_1)+\exp(\beta_2+\alpha_1)+\exp(\beta_0+\beta_2+2\alpha_1)}\right\}<0$$

$$\Rightarrow \exp(\beta_1)<\frac{1+\exp(\beta_0+\beta_2+\alpha_1)+\exp(\alpha_1)+\exp(\beta_0+\beta_2+2\alpha_1)}{1+\exp(\beta_0+\alpha_1)+\exp(\beta_2+\alpha_1)+\exp(\beta_0+\beta_2+2\alpha_1)}<1$$

$$
\begin{aligned}
\Rightarrow T(\beta_1)&=(\exp(\beta_2)-1)\times\exp(\alpha_0)\\
&\times\{\exp(\beta_1)\times[1+\exp(\beta_0+\alpha_1)+\exp(\beta_2+\alpha_1)+\exp(\beta_0+\beta_2+2\alpha_1)]\\
&-[1+\exp(\beta_0+\beta_2+\alpha_1)+\exp(\alpha_1)+\exp(\beta_0+\beta_2+2\alpha_1)]\}\\
&<0
\end{aligned}
$$

Therefore, when $\beta_2>0$, $\beta_1<0$, $\beta_0<0$, then $bias>0$.

③ $\beta_1>0$:(i) $\beta_0=0$, $bias<0$;(ii) $\beta_0<0$, $bias<0$;(iii) $\beta_0>0$, $bias<0$.

proof (iii)

$$
\begin{aligned}
T(\beta_1)&=(\exp(\beta_2)-1)\times\exp(\alpha_0)\\
&\times\{\exp(\beta_1)\times[1+\exp(\beta_0+\alpha_1)+\exp(\beta_2+\alpha_1)+\exp(\beta_0+\beta_2+2\alpha_1)]\\
&-[1+\exp(\beta_0+\beta_2+\alpha_1)+\exp(\alpha_1)+\exp(\beta_0+\beta_2+2\alpha_1)]\}
\end{aligned}
$$

when $\beta_0>0$ and $\beta_2>0\Rightarrow\exp(\beta_0)-1>0\quad \exp(\beta_2)-1>0$

According to $(a-1)(b-1)=ab-a-b+1$, when $ab>0\Rightarrow ab+1>a+b$

$$
\begin{aligned}
&1+\exp(\beta_0+\beta_2+\alpha_1)+\exp(\alpha_1)+\exp(\beta_0+\beta_2+2\alpha_1)\\
&>1+\exp(\beta_0+\alpha_1)+\exp(\beta_2+\alpha_1)+\exp(\beta_0+\beta_2+2\alpha_1)\\
&\Rightarrow \exp(\beta_0+\beta_2)+1>\exp(\beta_0)+\exp(\beta_2)
\end{aligned}
$$

when

$$\beta_1>\log\left\{\frac{\exp(\beta_0+\beta_2)+1}{\exp(\beta_0)+\exp(\beta_2)}\right\}>0$$

$$\beta_1>\log\left\{\frac{1+\exp(\beta_0+\beta_2+\alpha_1)+\exp(\alpha_1)+\exp(\beta_0+\beta_2+2\alpha_1)}{1+\exp(\beta_0+\alpha_1)+\exp(\beta_2+\alpha_1)+\exp(\beta_0+\beta_2+2\alpha_1)}\right\}>0$$

$$\Rightarrow \exp(\beta_1)>\frac{1+\exp(\beta_0+\beta_2+\alpha_1)+\exp(\alpha_1)+\exp(\beta_0+\beta_2+2\alpha_1)}{1+\exp(\beta_0+\alpha_1)+\exp(\beta_2+\alpha_1)+\exp(\beta_0+\beta_2+2\alpha_1)}>1$$

$$
\begin{aligned}
\Rightarrow T(\beta_1) &= (\exp(\beta_2)-1)\times\exp(\alpha_0) \\
&\times\{\exp(\beta_1)\times[1+\exp(\beta_0+\alpha_1)+\exp(\beta_2+\alpha_1)+\exp(\beta_0+\beta_2+2\alpha_1)] \\
&-[1+\exp(\beta_0+\beta_2+\alpha_1)+\exp(\alpha_1)+\exp(\beta_0+\beta_2+2\alpha_1)]\} \\
&> 0
\end{aligned}
$$

Therefore, when $\beta_2 > 0$, $\beta_1 > 0$, $\beta_0 > 0$, then $bias < 0$.

3: $\beta_2 < 0$,

① $\beta_1 = 0$:(i) $\beta_0 = 0$, $bias = 0$;(ii) $\beta_0 > 0$, $bias > 0$;(iii) $\beta_0 < 0$, $bias < 0$.

② $\beta_1 < 0$:(i) $\beta_0 = 0$, $bias < 0$;(ii) $\beta_0 < 0$, $bias < 0$;(iii) $\beta_0 > 0$, $bias < 0$.

proof (iii)

$$
\begin{aligned}
T(\beta_1) &= (\exp(\beta_2)-1)\times\exp(\alpha_0) \\
&\times\{\exp(\beta_1)\times[1+\exp(\beta_0+\alpha_1)+\exp(\beta_2+\alpha_1)+\exp(\beta_0+\beta_2+2\alpha_1)] \\
&-[1+\exp(\beta_0+\beta_2+\alpha_1)+\exp(\alpha_1)+\exp(\beta_0+\beta_2+2\alpha_1)]\}
\end{aligned}
$$

when $\beta_0 > 0$ and $\beta_2 < 0 \Rightarrow \exp(\beta_0)-1 > 0 \qquad \exp(\beta_2)-1 < 0$

According to $(a-1)(b-1) = ab-a-b+1$, when $ab < 0 \Rightarrow ab+1 < a+b$

$$
\begin{aligned}
&1+\exp(\beta_0+\beta_2+\alpha_1)+\exp(\alpha_1)+\exp(\beta_0+\beta_2+2\alpha_1) \\
&< 1+\exp(\beta_0+\alpha_1)+\exp(\beta_2+\alpha_1)+\exp(\beta_0+\beta_2+2\alpha_1) \\
&\Rightarrow \exp(\beta_0+\beta_2)+1 < \exp(\beta_0)+\exp(\beta_2)
\end{aligned}
$$

when

$$\beta_1 < \log\left\{\frac{\exp(\beta_0+\beta_2)+1}{\exp(\beta_0)+\exp(\beta_2)}\right\} < 0$$

$$\beta_1 < \log\left\{\frac{1+\exp(\beta_0+\beta_2+\alpha_1)+\exp(\alpha_1)+\exp(\beta_0+\beta_2+2\alpha_1)}{1+\exp(\beta_0+\alpha_1)+\exp(\beta_2+\alpha_1)+\exp(\beta_0+\beta_2+2\alpha_1)}\right\} < 0$$

$$\Rightarrow \exp(\beta_1) < \frac{1+\exp(\beta_0+\beta_2+\alpha_1)+\exp(\alpha_1)+\exp(\beta_0+\beta_2+2\alpha_1)}{1+\exp(\beta_0+\alpha_1)+\exp(\beta_2+\alpha_1)+\exp(\beta_0+\beta_2+2\alpha_1)} < 1$$

$$
\begin{aligned}
\Rightarrow T(\beta_1) &= (\exp(\beta_2)-1)\times\exp(\alpha_0) \\
&\times\{\exp(\beta_1)\times[1+\exp(\beta_0+\alpha_1)+\exp(\beta_2+\alpha_1)+\exp(\beta_0+\beta_2+2\alpha_1)] \\
&-[1+\exp(\beta_0+\beta_2+\alpha_1)+\exp(\alpha_1)+\exp(\beta_0+\beta_2+2\alpha_1)]\} \\
&> 0
\end{aligned}
$$

Therefore, when $\beta_2 < 0$, $\beta_1 < 0$, $\beta_0 > 0$, then $bias < 0$.

③ $\beta_1 > 0$:(i) β_0=0, $bias > 0$;(ii) $\beta_0 > 0$, $bias > 0$;(iii) $\beta_0 < 0$, $bias > 0$.

proof (iii)

$$T(\beta_1) = (\exp(\beta_2) - 1) \times \exp(\alpha_0)$$
$$\times\{\exp(\beta_1) \times [1 + \exp(\beta_0 + \alpha_1) + \exp(\beta_2 + \alpha_1) + \exp(\beta_0 + \beta_2 + 2\alpha_1)]$$
$$-[1 + \exp(\beta_0 + \beta_2 + \alpha_1) + \exp(\alpha_1) + \exp(\beta_0 + \beta_2 + 2\alpha_1)]\}$$

when $\beta_0 < 0$ and $\beta_2 < 0 \Rightarrow \exp(\beta_0) - 1 < 0 \quad \exp(\beta_2) - 1 < 0$

According to $(a-1)(b-1) = ab - a - b + 1$, when $ab > 0 \Rightarrow ab + 1 > a + b$

$$1 + \exp(\beta_0 + \beta_2 + \alpha_1) + \exp(\alpha_1) + \exp(\beta_0 + \beta_2 + 2\alpha_1)$$
$$> 1 + \exp(\beta_0 + \alpha_1) + \exp(\beta_2 + \alpha_1) + \exp(\beta_0 + \beta_2 + 2\alpha_1)$$
$$\Rightarrow \exp(\beta_0 + \beta_2) + 1 > \exp(\beta_0) + \exp(\beta_2)$$

when

$$\beta_1 > \log\left\{\frac{\exp(\beta_0 + \beta_2) + 1}{\exp(\beta_0) + \exp(\beta_2)}\right\} > 0$$

$$\beta_1 > \log\left\{\frac{1 + \exp(\beta_0 + \beta_2 + \alpha_1) + \exp(\alpha_1) + \exp(\beta_0 + \beta_2 + 2\alpha_1)}{1 + \exp(\beta_0 + \alpha_1) + \exp(\beta_2 + \alpha_1) + \exp(\beta_0 + \beta_2 + 2\alpha_1)}\right\} > 0$$

$$\Rightarrow \exp(\beta_1) > \frac{1 + \exp(\beta_0 + \beta_2 + \alpha_1) + \exp(\alpha_1) + \exp(\beta_0 + \beta_2 + 2\alpha_1)}{1 + \exp(\beta_0 + \alpha_1) + \exp(\beta_2 + \alpha_1) + \exp(\beta_0 + \beta_2 + 2\alpha_1)} > 1$$

$$\Rightarrow T(\beta_1) = (\exp(\beta_2) - 1) \times \exp(\alpha_0)$$
$$\times\{\exp(\beta_1) \times [1 + \exp(\beta_0 + \alpha_1) + \exp(\beta_2 + \alpha_1) + \exp(\beta_0 + \beta_2 + 2\alpha_1)]$$
$$-[1 + \exp(\beta_0 + \beta_2 + \alpha_1) + \exp(\alpha_1) + \exp(\beta_0 + \beta_2 + 2\alpha_1)]\}$$
$$< 0$$

Therefore, when $\beta_2 < 0$, $\beta_1 > 0$, $\beta_0 < 0$, then $bias > 0$.

In conclusion:

1: $\beta_2 = 0$, $bias = 0$.

2: $\beta_2 \neq 0$, $\beta_1 = 0$:(i) $\beta_0 = 0$, $bias = 0$;(ii) $\beta_0 > 0$, $bias > 0$;(iii) $\beta_0 < 0$, $bias < 0$.

3: (i) $\beta_1\beta_2 > 0$, $bias < 0$. (ii) $\beta_1\beta_2 < 0$, $bias > 0$.
