# Supplementary material for: Sensitivity analysis for mistakenly adjusting for mediators in estimating total effect in observational studies
Source: BMJ Open. 2017 Nov 20;7(11):e015640. doi: 10.1136/bmjopen-2016-015640 (PMC5719285; doi:10.1136/bmjopen-2016-015640)
Supplement: Author's manuscript [file bmjopen-2016-015640.draft_revisions.pdf]

BMJ Open is committed to open peer review. As part of this commitment we make the peer review history of every article we publish publicly available.

When an article is published we post the peer reviewers' comments and the authors' responses online. We also post the versions of the paper that were used during peer review. These are the versions that the peer review comments apply to.

The versions of the paper that follow are the versions that were submitted during the peer review process. They are not the versions of record or the final published versions. They should not be cited or distributed as the published version of this manuscript.

BMJ Open is an open access journal and the full, final, typeset and author-corrected version of record of the manuscript is available on our site with no access controls, subscription charges or pay-per-view fees (http://bmjopen.bmj.com).

If you have any questions on BMJ Open's open peer review process please email info.bmjopen@bmj.com

BMJ Open

A Sensitivity Analysis for Mistaking Mediators as Confounders in the Perspective of Causal Diagrams: a Simulation Study

Journal:	*BMJ Open*
Manuscript ID	bmjopen-2016-015640
Article Type:	Research
Date Submitted by the Author:	22-Dec-2016
Complete List of Authors:	Wang, Tingting; Shandong University, Department of Epidemiology and Biostatistics<br>Li, Hongkai; Shandong University, Department of Epidemiology and Biostatistics<br>Su, Ping; Shandong University, Department of Epidemiology and Biostatistics<br>Yu, Yuanyuan; Shandong University, Department of Epidemiology and Biostatistics<br>Sun, Xiaoru; Shandong University, Department of Epidemiology and Biostatistics<br>Liu, Yi; Shandong University, Department of Epidemiology and Biostatistics<br>Yuan, Zhongshang; Shandong University, Department of Epidemiology and Biostatistics<br>Xue, Fuzhong; Shandong University, Department of Epidemiology and Biostatistics
**Primary Subject Heading**:	Epidemiology
Secondary Subject Heading:	Public health
Keywords:	EPIDEMIOLOGY, PUBLIC HEALTH, STATISTICS & RESEARCH METHODS

SCHOLARONE™
Manuscripts

A Sensitivity Analysis for Mistaking Mediators as Confounders in the Perspective of Causal Diagrams: a Simulation Study

Tingting Wang[†], Hongkai Li[†], Ping Su, Yuanyuan Yu, Xiaoru Sun, Yi Liu, Zhongshang Yuan, Fuzhong Xue*
Department of biostatistics, School of Public Health, Shandong University, Jinan City, Shandong Province, P.R.China
Email addresses:
TTW: wangtingting161780@163.com
HKL: lihongkaiyouxiang@163.com
PS: sdsuping@163.com
YYY: yu_yy_1993@163.com
XRS: sunny150139@126.com
YL: liuyi238@sdu.edu.cn
ZSY: yuanzhongshang@sdu.edu.cn

*Corresponding Author
xuefzh@sdu.edu.cn (FZX)

Word Count: 2899

Abstract

Objectives: In observational studies, when the underlying structure is unknown and only limited knowledge is available, a sensitivity analysis between the effect of exposure-mediator and the effect of mediator-outcome was dissected under mistakenly controlling for mediators to estimate the total effect of exposure on outcome. Through simulation, we focused on six causal diagrams concerning different roles of mediators to compare the sensitivity of the effect of exposure-mediator with the effect of mediator-outcome in adjusting for mediator under the framework of logistic regression model.

Setting: Based on the causal relationships in real world, we generated the simulation data by varying across the effect of exposure-mediator and the effect of mediator-outcome. And compared the bias of varying across the effect exposure-mediator with the bias of varying across the effect mediator-outcome mistakenly adjusting for mediator. The magnitude of bias was defined by the

For peer review only - http://bmjopen.bmj.com/site/about/guidelines.xhtml

difference between the estimated causal effect by logistic regression models and the true causal effect based on *do calculus*.

Results: Simulation results revealed that, when there are only a single mediator, two series mediators, two independent parallel mediators and two correlated parallel mediators, the bias that varied across the effect exposure-mediator was larger than the one that varied across the effect mediator-outcome under adjusting for the mediator. However, the bias performances were opposite result in scenarios of a single mediator and two independent parallel mediators in the presence of unobserved confounders.

Conclusions: We concluded that the sensitivity between the effect exposure-mediator and the effect mediator-outcome was related to whether there is unobserved confounder in causal diagrams.

Keywords: observational study; mediator; confounder; causal diagram; sensitivity analysis

Strengths and limitations of this study

Based on the *do calculus* calculated the total causal effect of exposure on outcome in perspective of causal diagram.

Various simulations were conducted to assess the consequences between the effect of exposure-mediator (E→M) and the effect of mediator-outcome (M→D) under mistakenly adjust for mediator in logistic regression model.

The simulated parameters were based on the observational study.

The limitation was only considered the binary variable and was not known the conclusion of continuous variable.

Introduction

Estimating the total effects of the exposure (E) on the outcome (D) is still a great challenge in the analytic epidemiology study, because researchers often do not fully

For peer review only - http://bmjopen.bmj.com/site/about/guidelines.xhtml

acknowledge the distinction between confounders and mediators.[1-3] If confounders and mediators are misclassified, the ability to control confounder in the estimation of the total effect of the exposure on the outcome is hampered. Causal diagrams have provided a formal conceptual framework to identify and select confounders,[4-5] so that it can avoid falling into analytic pitfalls.[6] In practice, even the underlying causal diagrams and the role of covariates (mediator, confounder, collider and instrumental variable) are not all known, investigators usually controlled the covariates that are both associated with the outcome and exposure to estimate the total effect of the exposure on the outcome.[7-10] Therefore, our paper paid attention to the bias behavior that varied across the effect exposure-mediator (E→M) and the effect mediator-outcome (M→D) under mistakenly regraded mediator as confounder in logistic regression model.

Recently, epidemiologists mainly explore the mediator mechanisms of the total effect, direct effect and indirect effect of exposure on outcome.[11-13] Arbitrarily controlling for a mediator would generally obtain biased estimates of the total effect of the exposure on the outcome.[6, 14-15] Nevertheless, in the perspective of causal diagrams, little attention was paid to the consequences of biases of mistakenly adjusting for mediators in the logistic regression model. Hence, we focused on the sensitivity analysis technique to assess the impact between the effect E→M and the effect M→D with adjusting for mediator under the framework of logistic regression model.

In this paper, six typical causal diagrams corresponding to causal correlation are given in Figure 1. We considered a study examining the effect of a potentially beneficial exposure E on outcome D and explored the sensitivity of the effect E→D

For peer review only - http://bmjopen.bmj.com/site/about/guidelines.xhtml

For peer review only

and the effect M→D. And we performed various quantitative simulations to dissect the bias that varied across the effect E→M and the one that varied across the effect M→D under the models of adjusting for different mediators. It may provide a guide for studying the importance between the effect magnitude of pathway and the direction from exposure to mediator or from mediator to outcome.

Methods

A directed acyclic graph (DAG) is composed of variables (nodes) and arrows (directed edges) between nodes such that the graph is acyclic. Pearl formalized causal diagrams as directed acyclic graphs (DAGs), providing investigators with powerful tools for bias assessment.[16] The causal directed acyclic graph theory provides a device for deducing the statistical associations implied by causal relations. Furthermore, given a set of observed statistical associations, a researcher armed with causal diagrams theory can systematically characterize all causal structures compatible with the observations.[17-18]

The total causal effect can be calculated based on the *do-calculus* and *back-door* criterion proposed by Judea Pearl.[19-20] For exposure X and outcome Y, a set of variables Z is said to satisfy the backdoor path criterion with respect to (X, Y) if no variable in Z is a descendant of X and if Z blocks all back-door paths from X to Y. Then the causal effect of X on Y is given by the formula,

$$P(y \mid do(x)) = \sum_{Z} P(y \mid x, z) P(z)$$

Note that the expression on the right hand side of the equation is simply a standardized mean. The difference $E(Y \mid do(x')) - E(Y \mid do(x''))$ is taken as the definition of "causal effect", where x' and x'' are two distinct realizations of X.[19]

For peer review only - http://bmjopen.bmj.com/site/about/guidelines.xhtml

Besides it can be shown that if ignorability holds for $Y(x)$ and X (alternatively if there are no back-door paths from X to Y in the corresponding causal DAGs), then $p(y \mid do(x)) = p(y \mid x)$.[21-22] Taking Figure 1a as an example, the true total causal effect (β) of E on D on the scale of logarithm odds ratio was equal to

$$\beta = \text{logit}(P(D=1 \mid do(E=1))) - \text{logit}(P(D=1 \mid do(E=0)))$$
$$= \text{logit}(\sum_M P(D=1 \mid E=1, M)) - \text{logit}(\sum_M P(D=1 \mid E=0, M))$$

The estimation after adjusting for M was equal to

$$\beta_M = \text{logit}(P(D=1 \mid E=1, M)) - \text{logit}(P(D=1 \mid E=0, M))$$

Note that the bias was defined by taking a difference between estimated exposure effect by adjusting for mediator using logistic regression and the true total causal effect based on *do calculus* i.e. $bias = \beta_M - \beta$. We dissected the biases behavior between the effect E→M and the effect M→D mistakenly controlling mediator under logistic regression model.

Simulation

As shown in Figure 1, six scenarios were designed to dissect bias behaviors caused by mistaking mediators as confounders using logistic regression model. We made the following assumptions for the simulation: 1) all variables were binary following a Bernoulli distribution; 2) the effect from parent nodes to their child node were positive and log-linearly additive. Taking Figure 1a as an example, we randomly generated the exposure E following a Bernoulli distribution (i.e. let $P(E=1) = \pi$), Then, $P_M = \exp(\alpha_0 + c_1 E)/(1+\exp(\alpha_0 + c_1 E))$ for calculating the distribution probability of child node M from its parent node E, similarity, $P_D = \exp(\beta_0 + c_2 M + c_0 E)/(1+\exp(\beta_0 + c_2 M + c_0 E))$ to generate the distribution

For peer review only - http://bmjopen.bmj.com/site/about/guidelines.xhtml

probability of D, where the parameters α_0 and β_0 denoted the intercept of M and D respectively, and effect parameter c_1, c_2, c_0 referred to the effects of the parent node on their corresponding child node using log odds ratio scale.

After generating data, we have dissected the biases behavior between the effect E→M and the effect M→D mistakenly controlling mediator under logistic regression model. In scenario 1 (Figure 1a), we compared the bias that varied across the effect E→M with the one that varied across the effect M→D with adjusting for mediator M under the logistic regression model. Similarly, in scenario 2 (Figure 1b), the bias that varied across the effect E→M_1 and the effect M_1→M_2 with the bias that varied across the effect M_2→D were explored with adjustment for M_1, adjustment for M_2 and adjustment for M_1 M_2 under the logistic regression model, respectively. In scenario 3 (Figure 1c), we dissected the bias that varied across the effect E→M_1 with the bias that varied across the effect M_1→D and payed close attention to the bias that varied across the effect E→M_2 with the bias that varied across the effect M_2→D with adjustment for M_1, adjustment for M_2 and adjustment for M_1 M_2 under the logistic regression model, respectively. The biases comparison of scenario 4 (Figure 1d) were same as scenario 3 (Figure 1c). In scenario 5 (Figure 1e), we excavated the bias that varied across the effect E→M and the bias that varied across the effect M→D with adjustment for M under the logistic regression model. In scenario 6 (Figure 1f), we compared the bias that varied across the effect E→M_1 with the bias that varied across the effect M_1→D and explored the bias that varied across the effect E→M_2 with the bias that varied across the effect M_2→D with adjustment for M_1, adjustment for M_2

For peer review only - http://bmjopen.bmj.com/site/about/guidelines.xhtml

and adjustment for M_1 M_2 under the logistic regression model, respectively. We explored the biases behavior with adjusting the mediator under logistic regression model and thus identified the sensitivity between effect of exposure-mediator and effect of mediator-outcome.

For each of 6 simulation scenarios, we observed bias performances of varying across distinct effects under adjusting mediator using logistic regression model with 1000 simulations repetitions. All simulations were conducted using software R from CRAN (http://cran.r-project.org/).

Results

Scenario 1: one single mediator (Figure 1a)

For Figure 1(a) of the simplest case, E has a direct (E→D) effect and an indirect (E→M→D) effect on D. Figure 2A depicted that the bias that varied across the effect E→M was obviously larger than the bias that varied across the effect M→D. In particular, if the effect E→M was specified to zero in Figure 2B, M became an independent cause of the outcome, and in this case adjusting for M obtained a positive bias. Moreover, Figure 2 indicated that adjusting for mediator M using logistic regression model was indeed biased to the total effect of the exposure on the outcome. The true total causal effect (β) of E on D was calculated as

$$\beta = \log(OR) = \log(\frac{P(D=1 \mid do(E=1))P(D=0 \mid do(E=0))}{P(D=0 \mid do(E=1))P(D=1 \mid do(E=0))})$$

$$= \log(\frac{\sum_M P(D=1 \mid E=1, M)P(M \mid E=1)\sum_M P(D=0 \mid E=0, M)P(M \mid E=0)}{\sum_M P(D=0 \mid E=1, M)P(M \mid E=1)\sum_M P(D=1 \mid E=0, M)P(M \mid E=0)})$$

By conditioning on mediator M, the effect of E on D was equal to

$$\beta_M = \log(OR_M) = \log(\frac{P(D=1 \mid E=1, M)P(D=0 \mid E=0, M)}{P(D=0 \mid E=1, M)P(D=1 \mid E=0, M)})$$

For peer review only - http://bmjopen.bmj.com/site/about/guidelines.xhtml

After a series of derivations (Appendix 1), we obtained $bias = 0$ under condition of $c_2 = 0$ (c_2 of the effect M→D), suggesting that the estimation of E on D was unbiased under adjusting for M when the effect M→D (c_2) was null. Only if the $c_2 \neq 0$ and $c_1 = 0$ (c_1 of the effect E→M), 1) while $bias = 0$, if $c_0 = 0$ (c_0 the effect E→D), indicating that the estimation of E on D was unbiased with adjusting for M; 2) the $bias > 0$, if $c_0 > 0$, indicating that the effect of E on D overestimated with adjusting for M; 3) the $bias < 0$, if $c_0 > 0$, indicating that the effect of E on D underestimated with adjusting for M. And we gained $bias > 0$, if $c_1 c_2 < 0$, suggesting that the effect of E on D overestimated with adjusting for M when the effect E→M (c_1) and the effect M→D (c_2) were opposite (i.e. the effect E→M was positive ($c_1 > 0$) and the effect M→D was negative ($c_2 < 0$) or the effect E→M was negative ($c_1 < 0$) and the effect M→D was positive ($c_2 > 0$)). The result was $bias < 0$ under conditions of $c_1 c_2 > 0$, indicating that the effect of E on D underestimated with adjusting for M when the effect E→M (c_1) and the effect M→D (c_2) were positive or the effect E→M (c_1) and the effect M→D (c_2) were negative. The detail of theoretical derivation was in Appendix1.

Scenario 2: two series mediators (Figure 1b)

Figure 1(b) is a depiction through two series mediators, decomposing total effects into direct (E→D) and indirect (E→M_1→M_2→D) components. The bias that varied across the effect E→M_1 was larger than the one that varied across the effect M_2→D under adjustment for M_1, adjustment for M_2 and adjustment for M_1 M_2 in Figure 3, when the correlation of series mediators was strong to avoid M_2 became an independent cause

For peer review only - http://bmjopen.bmj.com/site/about/guidelines.xhtml

of the outcome.

Scenario 3: two independent parallel mediators (Figure 1c)

Figure 1c shows that the exposure E independently causes M_1 and M_2 and indirectly influences the outcome D through M_1 and M_2, forming three causal paths E→D, E→M_1→D and E→M_2→D. We obtained that the bias that varied across the effect E→M_1 was distinctly larger than the one that varied across the effect M_1→D under adjustment for M_1 in Figure 4A. However, the bias with the effect E→M_2 increasing was nearly equal to the one with the effect M_2→D increasing under identical adjustment for M_1 in Figure 4A. Then, an above similar result can be obtained the biases behavior in Figure 4B. In addition, Figure 4C indicated that biases that varied across the effect E→M_1 and varied across the effect E→M_2 were obviously larger than the one with the effect M_1→D and the effect M_2→D increasing under adjustment for M_1 M_2.

Scenario 4: two correlated parallel mediators (Figure 1d)

In Figure 1d, there exists five paths from E to D: E→D, E→M_1→D, E→M_2→D, E→M_1←M_2→D and E→M_2→M_1→D. In particular, the path E→M_1←M_2→D is a blocked path, due to the M_1 being a collider node. Figure 5A indicated that the bias of the effect E→M_1 was obviously larger than the one of the effect M_1→D under the adjustment for M_1 with the OR of effect increasing. However, the bias that varied across the effect E→M_2 was almost equal to the one that varied across the effect M_2→D under identical adjustment model. Similarly, Figure 5B showed an analogous result for biases behavior. Besides, Figure 5C manifested that biases that varied across

For peer review only - http://bmjopen.bmj.com/site/about/guidelines.xhtml

the effect E→M_1 and the effect E→M_2 were larger than the ones that varied across the effect M_1→D and the effect M_2→D under adjusting for M_1 and M_2. Simultaneously, the effect E→M_2 was more sensitive than the effect E→M_1, which adjustment for the collider node M_1 would partially open the path E→M_1←M_2→D.

Scenario 5: a single mediator with an unobserved confounder (Figure 1e)

For Figure 1e, in the framework of a causal diagram with exposure E, outcome D, mediator M and unobserved confounder U, it revealed that the bias that varied across the effect E→M was lower than the one that varied across the effect M→D in the presence of unobserved confounder that distorts the association between the exposure and outcome (E←U→D) in Figure 6.

Scenario 6: two parallel mediators with an unobserved confounder (Figure 1f)

As described above, Figure 1f is a depiction of two parallel mediators M_1 and M_2 with an unobserved confounder U. Figure 7A indicated that the bias that varied across the effect E→M_1 was obviously less than the one that varied across the effect M_1→D under the adjustment for M_1, while the bias with the effect E→M_2 increasing was larger than the bias with the effect M_2→D increasing under the identical adjustment for M_1. A similar result can also be obtained in Figure 7B. Besides, biases that varied across the effect E→M_1 and varied across the effect E→M_2 were distinctly less than the ones with the effect M_1→D and the effect M_2→D increasing under common model of adjusting for M_1 and M_2 (Figure 7C).

Discussion

In this study, we dissected the sensitivity of the bias that varied across the effect

For peer review only - http://bmjopen.bmj.com/site/about/guidelines.xhtml

exposure-mediator and the one that varied across the effect mediator-outcome with adjusting for mediators under the framework of logistic regression model. When there are a single mediator (Figure 1a in scenario 1), two series mediators (Figure 1b in scenario 2), two independent parallel (Figure 1c in scenario 3) and two correlated parallel mediators (Figure 1d in scenario 4), the bias that varied across the effect exposure-mediator was larger than the one that varied across the effect mediator-outcome under adjusting for the mediator (Figure 2, Figure 3, Figure 4 & Figure 5). However, there are a single mediator and two independent parallel mediators in the presence of the unobserved confounder (Figure 1e in scenario 5 & Figure 1f in scenario 6), which the opposite result was presented that the bias that varied across the effect mediator-outcome was larger than the one that varied across the effect exposure-mediator under adjusting for the mediator (Figure 6 & Figure 7).

Obviously, adjustment for mediator indeed led to bias for estimating the total effect of the exposure on outcome.[6, 14-15] Unfortunately, mediators and confounders were indistinguishable in terms of statistical association and conceptual grounds[3]. Investigators also paid little attention to the consequences of biases caused by mistaking mediators as confounders to estimate the total effect of exposure on outcome under logistic regression model. Most of the studies focused on the mediation effect analysis such as the calculation of direct effects and indirect effect.[15, 23-25] Our study results revealed that the effect exposure-mediator was more sensitive than the effect mediator-outcome under adjusting for the mediator in the absence of the unobserved confounder in causal diagrams (Figure 1a, Figure 1b, Figure 1c &

For peer review only - http://bmjopen.bmj.com/site/about/guidelines.xhtml

Figure 1d). Nevertheless, the opposite result that was presented that the effect mediator-outcome was more sensitive than the effect exposure-mediator in the presence of the unobserved confounder in causal diagrams (Figure 1e & Figure 1f). Therefore, the biases that varied across different effects depended on the causal diagrams framework whether there exited unobserved confounder.

Note that, in the perspective of diagrams, our simulation study was not comprehensive to evaluate the bias behavior of adjusting for the mediator in logistic regression, since it only considered binary variables, the certain scenarios of effect size and the common type of models. The present work ought to reinforce the mechanisms and conceptual frameworks of confounder and mediator form causal diagrams so as to assess the total effect, indirect effect and direct effect, hence avoid falling into analytic pitfalls.

Conclusion

In conclusion, we showed that the sensitivity between the effect exposure-mediator and the effect mediator-outcome was related to whether there is confounder in causal diagrams. The effect exposure-mediator was more sensitive than the effect mediator-outcome under adjusting for the mediator in the absence of unobserved confounder, however, the sensitivity was opposite in the presence of unobserved confounder.

Statements

Ethics approval and materials

Not applicable

Competing interests

The authors declare that they have no competing interests.

For peer review only - http://bmjopen.bmj.com/site/about/guidelines.xhtml

Funding

This work was supported by grants from National Natural Science Foundation of China (grant number 81573259)

Provenance and peer review

Not commissioned; externally peer reviewed.

Data sharing statement

No additional data are available.

Authors' contributions

TTW and HKL jointly conceived the idea behind the article and designed the study. TTW helped conduct the literature review, performed the simulation and prepared the first draft of the manuscript. PS, YYY, XRS, YL and ZSY participated in the design of the study and the revision of the manuscript. FZX advised on critical revision of the manuscript for important intellectual content. All authors read and approved the final manuscript.

Acknowledgments

We would like to thank anonymous reviewers and academic editors for providing us with constructive comments and suggestions and also wish to acknowledge our colleagues for their invaluable work.

Open Access

This is an Open Access article distributed in accordance with the Creative Commons Attribution Non Commercial (CC BY-NC 4.0) license, which permits others to distribute, remix, adapt, build upon this work noncommercially, and license their derivative works on different terms, provided the original work is properly cited and the use is non-commercial. See: http:// creativecommons.org/licenses/by-nc/4.0/

For peer review only

References

1 Bulterys M, Morgenstern H. Confounding or intermediate effect? An appraisal of iatrogenic bias in perinatal AIDS research. *Paediatr Perinat Epidemiol* 1993;7:387–394.

2 Herna´n MA, Hernandez-Diaz S, Werler MM, et al. Causal knowledge as a prerequisite for confounding evaluation: an application to birth defects epidemiology. *Am J Epidemiol* 2002;155:176–184.

3 MacKinnon DP, Krull JL, and Lockwood CM. Equivalence of the mediation, confounding and suppression effect. *Prevention Science* 2000;1:173–181.

4 Howards PP, Schisterman EF, Poole C, et al. "Toward a clearer definition of confounding"

For peer review only - http://bmjopen.bmj.com/site/about/guidelines.xhtml

revisited with directed acyclic graphs, *Am J Epidemiol* 2012;176:506–511.

5 Williamson EJ, Aitken Z, Lawrie J, et al. Introduction to causal diagrams for confounder selection. *Respirology*. 2014;19:303-311.

6 Wil cox AJ, Weinberg CR, Basso O. On the pitfalls of adjusting for gestational age at birth. *Am J Epidemiol*. 2011;174:1062–1068.

7 Herna´n MA, Hernandez-Diaz S, Werler MM, et al. Causal knowledge as a prerequisite for confounding evaluation: an application to birth defects epidemiology. *Am J Epidemiol* 2002; 155:176–84.

8 HK. Methodological issues of confounding in analytical epidemiology studies. Caspian J intern Med 2012;3:488-495.

9 Kamangar F MD. Confounding variable in epidemiology studies: Basics and beyond.Arch Tran Med. 2012;15:508-516.

10 Gaskins AJ, Schisterman EF. The effect of lipid adjustment on the analysis of environmental contaminants and the outcome of human health risks. *Methods Mol Biol* 2009;580:371-381.

11 Robins JM, Greenland S. Identifiability and exchangeability for direct and indirect effects. *Epidemiology* 1992;3:143–155.

12 Pearl J. Direct and indirect effects. Seventeenth conference of uncertainy in artificial intelligence, 2001. San Francisco, CA: Morgan Kaufmann, 2001.

13 Huang B, Sivaganesan S, Succop P, et al. Statistical assessment of mediational effects for logistic mediational models. *Statist. Med* 2004;23:2713-2728.

14 Robins J. The control of confounding by intermediate variable. *Statistics in Medicine*. 1989;8:679-701.

15 Schistermana EF, Coleb SR, Platt RW. Overadjustment Bias and Unnecessary Adjustment in Epidemiologic Studies. *Epidemiology*. 2009;20:488–495.

16 Pearl J. Causal diagrams for empirical research. *Biometrika*. 1995;82:669-688.

17 Pearl J. Causality: models, reasoning, and inference. *Econometric Theory.* 2003;19: 675-685.

18 Joffe M, Gambhir M, Chadeau-Hyam M, et al. Causal diagrams in systems epidemiology. *Emerg Themes Epidemiol*. 2012; 9:1-18.

19 Pearl, J. Causality: Models, reasoning, and inference. Cambridge: Cambridge University Press; 2000.

20 Pearl J. Causal inference in statistics: an overview. Statistics Surveys. 2009; 3:96–146.

21 Rubin DB. Should observational studies be designed to allow lack of balance in covariate distributions across treatment groups? *Statistics in Medicine*. 2009;28:1420-1423.

22 Shpitser I. Counterfactual graphical models for longitudinal mediation analysis with unobserved confounding. *Cognitive Science* 2013;37:1011-1035.

23 Lange T, Rasmussen M, Thygesen LC. Assessing natural direct and indirect effects through multiple pathways. *American Journal of Epidemiology*. 2013;179, 513–518.

24 Shpitser, Ilya and VanderWeele, Tyler J. "A complete graphical criterion for the adjustment formula in mediation analysis," *The International Journal of Biostatistics*.2011;7,1-24.

25 Pearl J. Direct and indirect effects. Seventeenth conference of uncertainy in artificial intelligence, 2001. San Francisco, CA: Morgan Kaufmann, 2001.

Figure 1： Six causal diagrams were designed for estimating the causal effect of E on D. a) a single mediator M; b) two series mediators M_1 and M_2; c) two independent

For peer review only - http://bmjopen.bmj.com/site/about/guidelines.xhtml

parallel mediators M_1 and M_2; d) two correlated parallel mediators M_1 and M_2; e) a single mediator with an unobserved confounder U; f) two independent parallel mediators M_1 and M_2 with an unobserved confounder U.

Figure 2: The biases with the effect E→M (red) and the effect M→D (blue) increasing, respectively. Comparison of the bias of different effects in adjustment mediator. The OR of target effect (e.g. E→M) from 1 to 10 given other effects fixed ln2 in Figure 2A. The OR of the effect M→D from 1 to 10 with the effect E→M being equal to zero in Figure 2B (Color figure online).

Figure 3: The biases with the effect E→M_1 (red), the effect M_1→M_2 (blue) and the effect M_2→D (black) increasing, respectively. Comparison of the bias of different effects in three adjustment models: A) adjustment for M_1, B) adjustment for M_2 and C) adjustment for M_1 and M_2. The OR of target effect (e.g. E→M_1) from 1 to 10 given the effect M_1→M_2 fixed ln8 and other effects fixed ln2 in Figure 3 (Color figure online).

Figure 4: The biases with the effect E→M_1 (red), the effect E→M_2 (blue), the effect M_1→D (black) and the effect M_2→D (green) increasing, respectively. Comparison of the bias of different effects in three adjustment models: A) adjustment for M_1, B) adjustment for M_2 and C) adjustment for M_1 and M_2. The OR of target effects (e.g. E →M_1) from 1 to 10 given other edges effects fixed ln2 in Figure 4 (Color figure online).

Figure 5: The biases with the effect E→M_1 (red), the effect E→M_2 (blue), the effect M_1→D (black), the effect M_2→D (green) and the effect M_1→M_2 (purple) increasing, respectively. Comparison of the bias of different effects in three adjustment models: A) adjustment for M_1, B) adjustment for M_2 and C) adjustment for M_1 and M_2. The OR of target effects (e.g. E→M_1) from 1 to 10 given other effects fixed ln2 in Figure 5 (Color figure online).

Figure 6: The biases with the effect E→M (red) and the effect M→D (blue) respectively. Comparison of the bias of different effects in adjustment mediator M. The OR of target effects (e.g. E→M) from 1 to 10 given the effects of causal edges fixed ln2 and the effect of confounder edges fixed ln5 in Figure ln8 (Color figure online).

Figure 7: The biases with the effect E→M_1 (red), the effect E→M_2 (blue), the effect M_1→D (black) and the effect M_2→D (green) respectively. Comparison of the bias of different effects in three adjustment models: A) adjustment for M_1, B) adjustment for M_2, and C) adjustment for M_1 and M_2. The OR of target effects (e.g. E→M_1) from 1 to 10 given the effects of causal edges fixed ln2 and the effect of confounder edges fixed ln5 in Figure 7 (Color figure online).

For peer review only - http://bmjopen.bmj.com/site/about/guidelines.xhtml

For peer review only

For peer review only - http://bmjopen.bmj.com/site/about/guidelines.xhtml

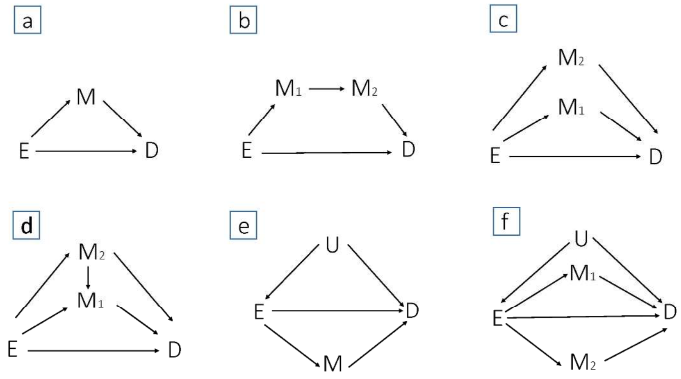

Six causal diagrams were designed for estimating the causal effect of E on D

338x190mm (300 x 300 DPI)

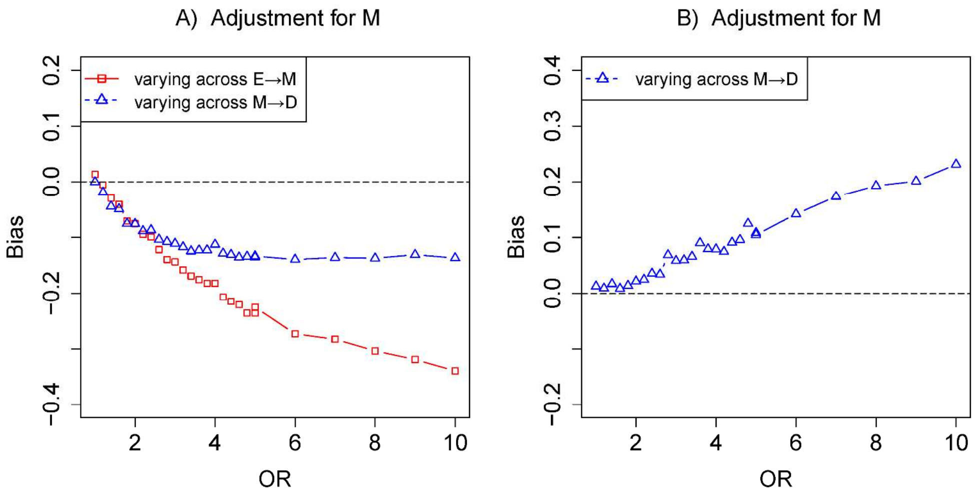

The biases with the effect E→M (red) and the effect M→D (blue) increasing, respectively.

281x148mm (300 x 300 DPI)

For peer review only

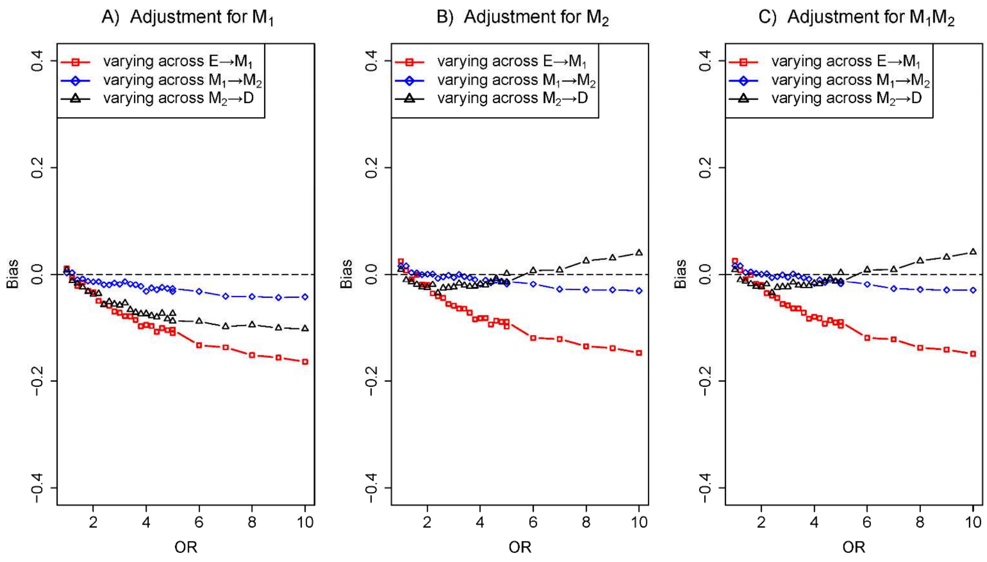

The biases with the effect E→M_1 (red), the effect M1→M_2 (blue) and the effect M_2→D (black) increasing, respectively.

270x155mm (300 x 300 DPI)

For peer review only - http://bmjopen.bmj.com/site/about/guidelines.xhtml

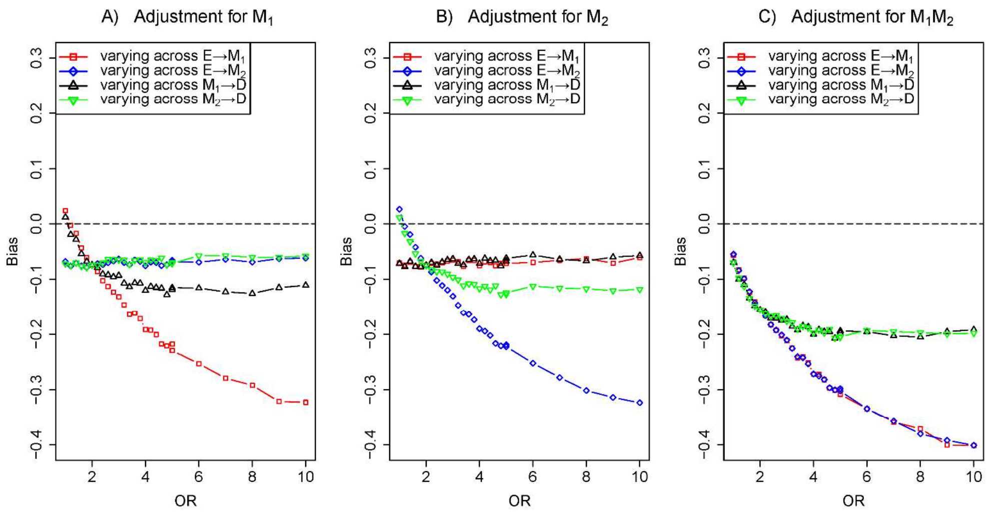

The biases with the effect E→M_1 (red), the effect E→M_2 (blue), the effect M_1→D (black) and the effect M_2→D (green) increasing, respectively.

279x147mm (300 x 300 DPI)

review only

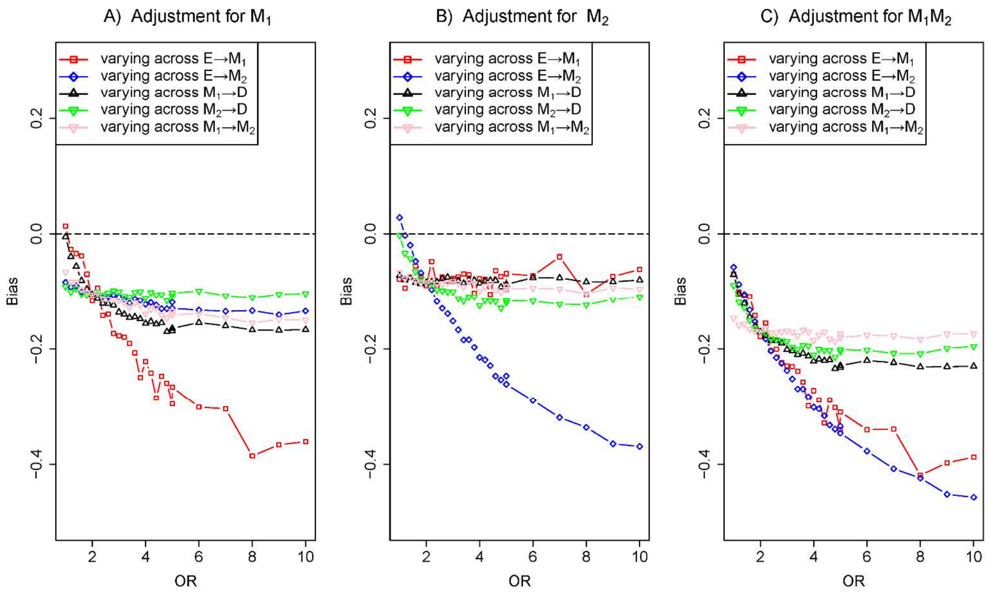

The biases with the effect E→M_1 (red), the effect E→M_2 (blue), the effect M_1→D (black), the effect M_2→D (green) and the effect M_1→M_2 (purple) increasing, respectively.

278x169mm (300 x 300 DPI)

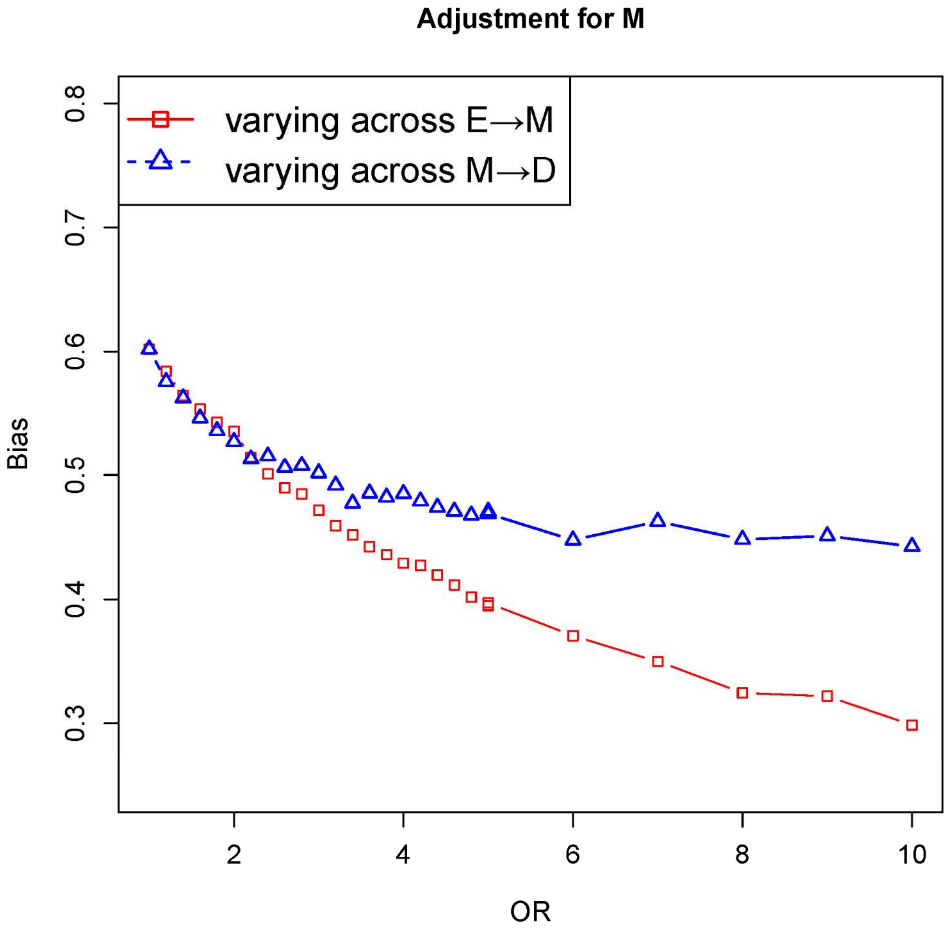

The biases with the effect E→M (red) and the effect M→D (blue) respectively.

177x177mm (300 x 300 DPI)

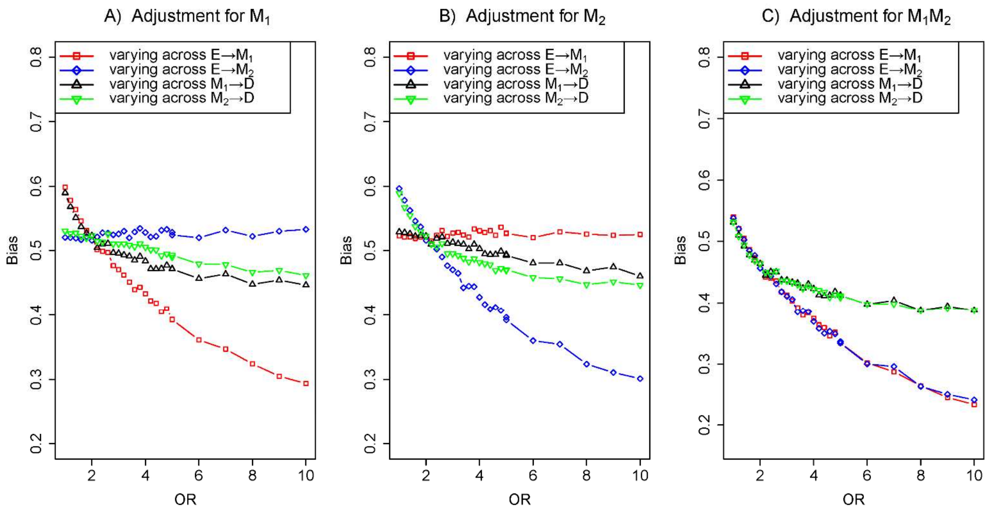

The biases with the effect E→M_1 (red), the effect E→M_2 (blue), the effect M_1→D (black) and the effect M_2→D (green) respectively.

281x148mm (300 x 300 DPI)

review only

Appendix 1:

Theoretical derivation was that adjusting for mediator was biased for estimating the total effect of exposure on outcome using logistic regression model. The bias was defined by taking a difference between estimated exposure effect by adjusting for mediator by logistic regression adjustment model and the true total effect based on *do calculus*.

Deducing the bias of Figure 1a in scenario 1 as follow:

Let E, M and D indicate exposure, mediator and outcome. The effect E→D, E→M and M→D defined to c_0, c_1 and c_2, respectively.

Suppose the logistic models among them are:

$$E \sim \text{Bernoulli}(1, P_E)$$

$$\text{logit}(P(M=1|E)) = \alpha_0 + c_1 E$$

$$\text{logit}(D=1|M,E) = \beta_0 + c_2 M + c_0 E$$

The total effect under do calculus ($\ln(OR)$):

$$OR = \frac{P(D=1|do(E=1))P(D=0|do(E=0))}{P(D=0|do(E=1))P(D=1|do(E=0))}$$
$$= \frac{\sum_M P(D=1|E=1,M)P(M|E=1)\sum_M P(D=0|E=0,M)P(M|E=0)}{\sum_M P(D=0|E=1,M)P(M|E=1)\sum_M P(D=1|E=0,M)P(M|E=0)}$$

The effect of adjusting for mediator M ($\ln(OR_M)$):

$$OR_M = \frac{P(D=1|E=1,M)P(D=0|E=0,M)}{P(D=0|E=1,M)P(D=1|E=0,M)}$$
$$= \frac{\frac{\exp(c_0 + c_2 \times M + \beta_0)}{1+\exp(c_0 + c_2 \times M + \beta_0)} \times \frac{1}{1+\exp(c_2 \times M + \beta_0)}}{\frac{1}{1+\exp(c_0 + c_2 \times M + \beta_0)} \times \frac{\exp(c_2 \times M + \beta_0)}{1+\exp(c_2 \times M + \beta_0)}}$$
$$= \exp(c_0)$$

where

$$A=\sum_{M}P(D=1\mid E=1,M)P(M\mid E=1)$$
$$=P(D=1\mid E=1,\ M=1)P(M=1\mid E=1)+P(D=1\mid E=1,\ M=0)P(M=0\mid E=1)$$
$$=\frac{\exp(c_0+c_2+\beta_0)}{1+\exp(c_0+c_2+\beta_0)}\times\frac{\exp(c_1+\alpha_0)}{1+\exp(c_1+\alpha_0)}+\frac{\exp(c_0+\beta_0)}{1+\exp(c_0+\beta_0)}\times\frac{1}{1+\exp(c_1+\alpha_0)}$$
$$B=\sum_{M}P(D=0\mid E=0,M)P(M\mid E=0)$$
$$=P(D=0\mid E=0,\ M=1)P(M=1\mid E=0)+P(D=0\mid E=0,\ M=0)P(M=0\mid E=0)$$
$$=\frac{1}{1+\exp(c_2+\beta_0)}\times\frac{\exp(\alpha_0)}{1+\exp(\alpha_0)}+\frac{1}{1+\exp(\beta_0)}\times\frac{1}{1+\exp(\alpha_0)}$$

$$C=\sum_{M}P(D=0\mid E=1,M)P(M\mid E=1)$$
$$=P(D=0\mid E=1,\ M=1)P(M=1\mid E=1)+P(D=0\mid E=1,\ M=0)P(M=0\mid E=1)$$
$$=\frac{1}{1+\exp(c_0+c_2+\beta_0)}\times\frac{\exp(c_1+\alpha_0)}{1+\exp(c_1+\alpha_0)}+\frac{1}{1+\exp(c_0+\beta_0)}\times\frac{1}{1+\exp(c_1+\alpha_0)}$$
$$D=\sum_{M}P(D=1\mid E=0,M)P(M\mid E=0)$$
$$=P(D=1\mid E=0,\ M=1)P(M=1\mid E=0)+P(D=1\mid E=0,\ M=0)P(M=0\mid E=0)$$
$$=\frac{\exp(c_2+\beta_0)}{1+\exp(c_2+\beta_0)}\times\frac{\exp(\alpha_0)}{1+\exp(\alpha_0)}+\frac{\exp(\beta_0)}{1+\exp(\beta_0)}\times\frac{1}{1+\exp(\alpha_0)}$$

Then $OR=\frac{AB}{CD}$

Reduction of fractions to a common denominator:

Then the numerators of A, B, C and D were defined by A_1, B_1, C_1 and D_1

$$A_1=\exp(c_0+c_2+\beta_0)\times\exp(c_1+\alpha_0)\times(1+\exp(c_0+\beta_0))+(1+\exp(c_0+c_2+\beta_0))\times\exp(c_0+\beta_0)$$
$$B_1=\exp(\alpha_0)\times(1+\exp(\beta_0))+(1+\exp(c_2+\beta_0))$$
$$C_1=\exp(c_1+\alpha_0)\times(1+\exp(c_0+\beta_0))+(1+\exp(c_0+c_2+\beta_0))$$
$$D_1=\exp(c_2+\beta_0)\times\exp(\alpha_0)\times(1+\exp(\beta_0))+\exp(\beta_0)\times(1+\exp(c_2+\beta_0))$$

Then

$$S_1=\exp(c_0+c_2+\beta_0)\times\exp(c_1+\alpha_0)\times(1+\exp(c_0+\beta_0))\times\exp(\alpha_0)\times(1+\exp(\beta_0))$$
$$S_2=\exp(c_0+c_2+\beta_0)\times\exp(c_1+\alpha_0)\times(1+\exp(c_0+\beta_0))\times(1+\exp(c_2+\beta_0))$$
$$S_3=(1+\exp(c_0+c_2+\beta_0))\times\exp(c_0+\beta_0)\times\exp(\alpha_0)\times(1+\exp(\beta_0))$$
$$S_4=(1+\exp(c_0+c_2+\beta_0))\times\exp(c_0+\beta_0)\times(1+\exp(c_2+\beta_0))$$
$$S_5=\exp(c_1+\alpha_0)\times(1+\exp(c_0+\beta_0))\times\exp(c_2+\beta_0)\times\exp(\alpha_0)\times(1+\exp(\beta_0))$$
$$S_6=\exp(c_1+\alpha_0)\times(1+\exp(c_0+\beta_0))\times\exp(\beta_0)\times(1+\exp(c_2+\beta_0))$$
$$S_7=(1+\exp(c_0+c_2+\beta_0))\times\exp(c_2+\beta_0)\times\exp(\alpha_0)\times(1+\exp(\beta_0))$$
$$S_8=(1+\exp(c_0+c_2+\beta_0))\times\exp(\beta_0)\times(1+\exp(c_2+\beta_0))$$
$$OR=\frac{S_1+S_2+S_3+S_4}{S_5+S_6+S_7+S_8}$$

For peer review only

Comparing the difference of S_1 and S_5, S_2 and S_6, S_3 and S_7, S_4 and S_8, respectively.

$$OR=\frac{\exp(c_0+c_2+\beta_0)\times A_2+\exp(c_0+c_2+\beta_0)\times B_2+\exp(c_0+\beta_0)\times C_2+\exp(c_0+\beta_0)\times D_2}{\exp(c_2+\beta_0)\times A_2+\exp(\beta_0)\times B_2+\exp(c_2+\beta_0)\times C_2+\exp(\beta_0)\times D_2}$$

$$=\exp(c_0)\frac{\exp(c_2)\times A_2+\exp(c_2)\times B_2+C_2+D_2}{\exp(c_2)\times A_2+B_2+\exp(c_2)\times C_2+D_2}$$

$$A_2=\exp(c_1+\alpha_0)\times(1+\exp(c_0+\beta_0))\times\exp(\alpha_0)\times(1+\exp(\beta_0))$$

$$B_2=\exp(c_1+\alpha_0)\times(1+\exp(c_0+\beta_0))\times(1+\exp(c_2+\beta_0))$$

$$C_2=(1+\exp(c_0+c_2+\beta_0))\times\exp(\alpha_0)\times(1+\exp(\beta_0))$$

$$D_2=(1+\exp(c_0+c_2+\beta_0))\times(1+\exp(c_2+\beta_0))$$

The effect of adjusting for intermediate M ($\ln(OR_M)$):

$$OR_M=\frac{P(D=1\mid E=1,M)P(D=0\mid E=0,M)}{P(D=0\mid E=1,M)P(D=1\mid E=0,M)}$$

$$=\frac{\frac{\exp(c_0+c_2\times M+\beta_0)}{1+\exp(c_0+c_2\times M+\beta_0)}\times\frac{1}{1+\exp(c_2\times M+\beta_0)}}{\frac{1}{1+\exp(c_0+c_2\times M+\beta_0)}\times\frac{\exp(c_2\times M+\beta_0)}{1+\exp(c_2\times M+\beta_0)}}$$

$$=\exp(c_0)$$

Therefore, we have

$$Bias=\ln(OR_M)\text{-}\ln(OR)$$

$$=\ln(\frac{OR_M}{OR})$$

$$=\ln(\frac{\exp(c_0)}{\exp(c_0)\frac{\exp(c_2)\times A_2+\exp(c_2)\times B_2+C_2+D_2}{\exp(c_2)\times A_2+B_2+\exp(c_2)\times C_2+D_2}})$$

$$=\ln(\frac{\exp(c_2)\times A_2+B_2+\exp(c_2)\times C_2+D_2}{\exp(c_2)\times A_2+\exp(c_2)\times B_2+C_2+D_2})$$

$$\frac{OR_M}{OR}=\frac{\exp(c_0)}{\exp(c_0)\frac{\exp(c_2)\times A_2+\exp(c_2)\times B_2+C_2+D_2}{\exp(c_2)\times A_2+B_2+\exp(c_2)\times C_2+D_2}}$$

$$=\frac{\exp(c_2)\times A_2+B_2+\exp(c_2)\times C_2+D_2}{\exp(c_2)\times A_2+\exp(c_2)\times B_2+C_2+D_2}$$

Focusing on the difference of between $\exp(c_2)\times B_2+C_2$ and $B_2+\exp(c_2)\times C_2$

The difference：

For peer review only

$$
\begin{aligned}
T(c_1) &= \exp(c_2)\times B_2 + C_2 - (B_2 + \exp(c_2)\times C_2) \\
&= \exp(c_2)\times(B_2 - C_2) - (B_2 - C_2) \\
&= (\exp(c_2)-1)\times(B_2 - C_2) \\
&= (\exp(c_2)-1)\times(\exp(c_1+\alpha_0)\times(1+\exp(c_0+\beta_0))\times(1+\exp(c_2+\beta_0)) - \\
&\quad (1+\exp(c_0+c_2+\beta_0))\times\exp(\alpha_0)\times(1+\exp(\beta_0))) \\
&= (\exp(c_2)-1)\times\exp(\alpha_0)\times(\exp(c_1)\times(1+\exp(c_0+\beta_0)\times(1+\exp(c_2+\beta_0)) - \\
&\quad (1+\exp(c_0+c_2+\beta_0))\times(1+\exp(\beta_0)))
\end{aligned}
$$

Then, detailed dissection:

1: $c_2 = 0, \dfrac{OR_M}{OR} = 1,\ OR_M = OR$ i.e. $bias = 0$

2： $c_2 > 0$, ① $c_1 = 0,\ c_0 = 0,\ \dfrac{OR_M}{OR} = 1$, i.e. $bias = 0$

$c_0 > 0, \dfrac{OR_M}{OR} > 1$, i.e. $bias > 0$

$c_0 < 0, \dfrac{OR_M}{OR} < 1$, i.e. $bias < 0$

② $c_1 < 0,\ c_0 = 0,\ \dfrac{OR_M}{OR} > 1$, i.e. $bias > 0$

$c_0 > 0, \dfrac{OR_M}{OR} > 1$, i.e. $bias > 0$

$c_0 < 0, \dfrac{OR_M}{OR} > 1$, i.e. $bias > 0$

In the proof

$$
\begin{aligned}
T(c_1) &= (\exp(c_2)-1)\times\exp(\alpha_0)\times(\exp(c_1)\times(1+\exp(c_0+\beta_0)+\exp(c_2+\beta_0)+\exp(c_0+c_2+2\beta_0)) - \\
&\quad (1+\exp(c_0+c_2+\beta_0)+\exp(\beta_0)+\exp(c_0+c_2+2\beta_0)))
\end{aligned}
$$

when $c_0 < 0$ and $c_2 > 0 \Rightarrow \exp(c_0) - 1 < 0 \quad \exp(c_2) - 1 > 0$

According to $(a-1)(b-1) = ab - a - b + 1$, when $(a-1)(b-1) < 0 \Rightarrow ab + 1 < a + b$

$$
\begin{aligned}
&1+\exp(c_0+c_2+\beta_0)+\exp(\beta_0)+\exp(c_0+c_2+2\beta_0) < 1+\exp(c_0+\beta_0)+\exp(c_2+\beta_0)+\exp(c_0+c_2+2\beta_0) \\
&\Rightarrow \exp(c_0+c_2)+1 < \exp(c_0)+\exp(c_2)
\end{aligned}
$$

When

$$
c_1 < \log\left(\frac{\exp(c_0+c_2)+1}{\exp(c_0)+\exp(c_2)}\right) < 0
$$

$$
c_1 < \log\left(\frac{1+\exp(c_0+c_2+\beta_0)+\exp(\beta_0)+\exp(c_0+c_2+2\beta_0)}{1+\exp(c_0+\beta_0)+\exp(c_2+\beta_0)+\exp(c_0+c_2+2\beta_0)}\right) < 0
$$

$$
\Rightarrow \exp(c_1) < \frac{1+\exp(c_0+c_2+\beta_0)+\exp(\beta_0)+\exp(c_0+c_2+2\beta_0)}{1+\exp(c_0+\beta_0)+\exp(c_2+\beta_0)+\exp(c_0+c_2+2\beta_0)} < 1
$$

$$
\begin{aligned}
\Rightarrow T(c_1) &= (\exp(c_2)-1)\times\exp(\alpha_0)\times(\exp(c_1)\times(1+\exp(c_0+\beta_0)+\exp(c_2+\beta_0)+\exp(c_0+c_2+2\beta_0)) - \\
&\quad (1+\exp(c_0+c_2+\beta_0)+\exp(\beta_0)+\exp(c_0+c_2+2\beta_0))) \\
&< 0
\end{aligned}
$$

For peer review only

Therefore, when $c_2 > 0$, $c_1 < 0$, $c_0 < 0$, then $\frac{OR_M}{OR} > 1$, i.e. $bias > 0$

$$③\ c_1 > 0, c_0 = 0, \frac{OR_M}{OR} < 1, \text{ i.e. } bias < 0$$

$$c_0 < 0, \frac{OR_M}{OR} < 1, \text{ i.e. } bias < 0$$

$$c_0 > 0, \frac{OR_M}{OR} < 1, \text{ i.e. } bias < 0$$

In the proof

$$T(c_1) = (\exp(c_2) - 1) \times \exp(\beta_0) \times (\exp(c_1) \times (1 + \exp(c_0 + \beta_0) + \exp(c_2 + \beta_0) + \exp(c_0 + c_2 + 2\beta_0)) - (1 + \exp(c_0 + c_2 + \beta_0) + \exp(\beta_0) + \exp(c_0 + c_2 + 2\beta_0)))$$

when $c_0 > 0$ and $c_2 > 0$ $\Rightarrow \exp(c_0) - 1 > 0$ $\exp(c_2) - 1 > 0$

According to $(a-1)(b-1) = ab - a - b + 1$, when $ab > 0 \Rightarrow ab + 1 > a + b$

$$1 + \exp(c_0 + c_2 + \beta_0) + \exp(\beta_0) + \exp(c_0 + c_2 + 2\beta_0) > 1 + \exp(c_0 + \beta_0) + \exp(c_2 + \beta_0) + \exp(c_0 + c_2 + 2\beta_0)$$
$$\Rightarrow \exp(c_0 + c_2) + 1 > \exp(c_0) + \exp(c_2)$$

When

$$c_1 > \log(\frac{\exp(c_0 + c_2) + 1}{\exp(c_0) + \exp(c_2)}) > 0$$

$$c_1 > \log(\frac{1 + \exp(c_0 + c_2 + \beta_0) + \exp(\beta_0) + \exp(c_0 + c_2 + 2\beta_0)}{1 + \exp(c_0 + \beta_0) + \exp(c_2 + \beta_0) + \exp(c_0 + c_2 + 2\beta_0)}) > 0$$

$$\Rightarrow \exp(c_1) > \frac{1 + \exp(c_0 + c_2 + \beta_0) + \exp(\beta_0) + \exp(c_0 + c_2 + 2\beta_0)}{1 + \exp(c_0 + \beta_0) + \exp(c_2 + \beta_0) + \exp(c_0 + c_2 + 2\beta_0)} > 1$$

$$\Rightarrow T(c_1) = (\exp(c_2) - 1) \times \exp(\alpha_0) \times (\exp(c_1) \times (1 + \exp(c_0 + \beta_0) + \exp(c_2 + \beta_0) + \exp(c_0 + c_2 + 2\beta_0)) - (1 + \exp(c_0 + c_2 + \beta_0) + \exp(\beta_0) + \exp(c_0 + c_2 + 2\beta_0))) > 0$$

Therefore, when $c_2 > 0$, $c_1 > 0$, $c_0 > 0$, then $\frac{OR_M}{OR} < 1$, i.e. $bias < 0$

3: $c_2 < 0$, ① $c_1 = 0$, $c_0 = 0$, $\frac{OR_M}{OR} = 1$, i.e. $bias = 0$

$$c_0 > 0, \frac{OR_M}{OR} > 1, \text{ i.e. } bias > 0$$

$$c_0 < 0, \frac{OR_M}{OR} < 1, \text{ i.e. } bias < 0$$

$$②\ c_1 < 0,\ c_0 = 0,\ \frac{OR_M}{OR} < 1, \text{ i.e. } bias < 0$$

$$c_0 < 0,\ \frac{OR_M}{OR} > 1, \text{ i.e. } bias < 0$$

$$c_0 > 0,\ \frac{OR_M}{OR} > 1, \text{ i.e. } bias < 0$$

In the proof

For peer review only

$$T(c_1) = (\exp(c_2) - 1) \times \exp(\beta_0) \times (\exp(c_1) \times (1 + \exp(c_0 + \beta_0) + \exp(c_2 + \beta_0) + \exp(c_0 + c_2 + 2\beta_0)) - (1 + \exp(c_0 + c_2 + \beta_0) + \exp(\beta_0) + \exp(c_0 + c_2 + 2\beta_0)))$$

when $c_0 > 0$ and $c_2 < 0$ $\Rightarrow \exp(c_0) - 1 > 0 \quad \exp(c_2) - 1 < 0$

According to $(a-1)(b-1) = ab - a - b + 1$, when $ab < 0 \Rightarrow ab + 1 < a + b$

$$1 + \exp(c_0 + c_2 + \beta_0) + \exp(\beta_0) + \exp(c_0 + c_2 + 2\beta_0) < 1 + \exp(c_0 + \beta_0) + \exp(c_2 + \beta_0) + \exp(c_0 + c_2 + 2\beta_0)$$
$$\Rightarrow \exp(c_0 + c_2) + 1 < \exp(c_0) + \exp(c_2)$$

When

$$c_1 < \log\left(\frac{\exp(c_0 + c_2) + 1}{\exp(c_0) + \exp(c_2)}\right) < 0$$
$$c_1 < \log\left(\frac{1 + \exp(c_0 + c_2 + \beta_0) + \exp(\beta_0) + \exp(c_0 + c_2 + 2\beta_0)}{1 + \exp(c_0 + \beta_0) + \exp(c_2 + \beta_0) + \exp(c_0 + c_2 + 2\beta_0)}\right) < 0$$
$$\Rightarrow \exp(c_1) < \frac{1 + \exp(c_0 + c_2 + \beta_0) + \exp(\beta_0) + \exp(c_0 + c_2 + 2\beta_0)}{1 + \exp(c_0 + \beta_0) + \exp(c_2 + \beta_0) + \exp(c_0 + c_2 + 2\beta_0)} < 1$$
$$\Rightarrow T(c_1) = (\exp(c_2) - 1) \times \exp(\alpha_0) \times (\exp(c_1) \times (1 + \exp(c_0 + \beta_0) + \exp(c_2 + \beta_0) + \exp(c_0 + c_2 + 2\beta_0)) - (1 + \exp(c_0 + c_2 + \beta_0) + \exp(\beta_0) + \exp(c_0 + c_2 + 2\beta_0))) > 0$$

Therefore, when $c_2 < 0$, $c_1 < 0$, $c_0 > 0$, then $\frac{OR_M}{OR} < 1$, i.e. $bias < 0$

③ $c_1 > 0$, $c_0 = 0$, $\frac{OR_M}{OR} > 1$, i.e. $bias > 0$

$c_0 > 0$, $\frac{OR_M}{OR} > 1$, i.e. $bias > 0$

$c_0 < 0$, $\frac{OR_M}{OR} > 1$, i.e. $bias > 0$

In the proof

$$T(c_1) = (\exp(c_2) - 1) \times \exp(\beta_0) \times (\exp(c_1) \times (1 + \exp(c_0 + \beta_0) + \exp(c_2 + \beta_0) + \exp(c_0 + c_2 + 2\beta_0)) - (1 + \exp(c_0 + c_2 + \beta_0) + \exp(\beta_0) + \exp(c_0 + c_2 + 2\beta_0)))$$

when $c_0 < 0$ and $c_2 < 0$ $\Rightarrow \exp(c_0) - 1 < 0 \quad \exp(c_2) - 1 < 0$

According to $(a-1)(b-1) = ab - a - b + 1$, when $ab > 0 \Rightarrow ab + 1 > a + b$

$$1 + \exp(c_0 + c_2 + \beta_0) + \exp(\beta_0) + \exp(c_0 + c_2 + 2\beta_0) > 1 + \exp(c_0 + \beta_0) + \exp(c_2 + \beta_0) + \exp(c_0 + c_2 + 2\beta_0)$$
$$\Rightarrow \exp(c_0 + c_2) + 1 > \exp(c_0) + \exp(c_2)$$

When

For peer review only

$$c_1 > \log\left(\frac{\exp(c_0+c_2)+1}{\exp(c_0)+\exp(c_2)}\right) > 0$$

$$c_1 > \log\left(\frac{1+\exp(c_0+c_2+\beta_0)+\exp(\beta_0)+\exp(c_0+c_2+2\beta_0)}{1+\exp(c_0+\beta_0)+\exp(c_2+\beta_0)+\exp(c_0+c_2+2\beta_0)}\right) > 0$$

$$\Rightarrow \exp(c_1) > \frac{1+\exp(c_0+c_2+\beta_0)+\exp(\beta_0)+\exp(c_0+c_2+2\beta_0)}{1+\exp(c_0+\beta_0)+\exp(c_2+\beta_0)+\exp(c_0+c_2+2\beta_0)} > 1$$

$$\begin{aligned}\Rightarrow T(c_1) = {} & (\exp(c_2)-1)\times\exp(\alpha_0)\times(\exp(c_1)\times(1+\exp(c_0+\beta_0)+\exp(c_2+\beta_0)+\exp(c_0+c_2+2\beta_0)) - \\ & (1+\exp(c_0+c_2+\beta_0)+\exp(\beta_0)+\exp(c_0+c_2+2\beta_0))) \\ & < 0\end{aligned}$$

Therefore, when $c_2 < 0, c_1 > 0, c_0 < 0$, then $\frac{OR_M}{OR} > 1$, i.e. $bias > 0$

In conclusion:

1: $c_2 = 0, \frac{OR_M}{OR} = 1$, i.e. $OR_M = OR$ i.e. $bias = 0$

2: $c_2 \neq 0, c_1 = 0, c_0 = 0$, $\frac{OR_M}{OR} = 1$, i.e. $bias = 0$

$c_0 > 0$, $\frac{OR_M}{OR} > 1$, i.e. $bias > 0$

$c_0 < 0$, $\frac{OR_M}{OR} < 1$, i.e. $bias < 0$

3: $c_1 c_2 > 0$, $\frac{OR_M}{OR} < 1$, i.e. $bias < 0$

$c_1 c_2 < 0$, $\frac{OR_M}{OR} > 1$, i.e. $bias > 0$

For peer review only

STROBE 2007 (v4) checklist of items to be included in reports of observational studies in epidemiology*

Checklist for cohort, case-control, and cross-sectional studies (combined)

Section/Topic	Item #	Recommendation	Reported on page #
**Title and abstract**	1	(*a*) Indicate the study's design with a commonly used term in the title or the abstract	1
		(*b*) Provide in the abstract an informative and balanced summary of what was done and what was found	2
**Introduction**			
Background/rationale	2	Explain the scientific background and rationale for the investigation being reported	3
Objectives	3	State specific objectives, including any pre-specified hypotheses	3-4
**Methods**			
Study design	4	Present key elements of study design early in the paper	4
Setting	5	Describe the setting, locations, and relevant dates, including periods of recruitment, exposure, follow-up, and data collection	5-6
Participants	6	(*a*) *Cohort study*—Give the eligibility criteria, and the sources and methods of selection of participants. Describe methods of follow-up<br>*Case-control study*—Give the eligibility criteria, and the sources and methods of case ascertainment and control selection. Give the rationale for the choice of cases and controls<br>*Cross-sectional study*—Give the eligibility criteria, and the sources and methods of selection of participants	5-6
		(*b*) *Cohort study*—For matched studies, give matching criteria and number of exposed and unexposed<br>*Case-control study*—For matched studies, give matching criteria and the number of controls per case	
Variables	7	Clearly define all outcomes, exposures, predictors, potential confounders, and effect modifiers. Give diagnostic criteria, if applicable	5-6
Data sources/ measurement	8*	For each variable of interest, give sources of data and details of methods of assessment (measurement). Describe comparability of assessment methods if there is more than one group	*5-6*
Bias	9	Describe any efforts to address potential sources of bias	5-6
Study size	10	Explain how the study size was arrived at	5-6
Quantitative variables	11	Explain how quantitative variables were handled in the analyses. If applicable, describe which groupings were chosen and why	Not applicable
Statistical methods	12	(*a*) Describe all statistical methods, including those used to control for confounding	4-6
		(*b*) Describe any methods used to examine subgroups and interactions	Not applicable
		(*c*) Explain how missing data were addressed	Not applicable
		(*d*) *Cohort study*—If applicable, explain how loss to follow-up was addressed<br>*Case-control study*—If applicable, explain how matching of cases and controls was addressed	Not applicable

For peer review only

		Cross-sectional study—If applicable, describe analytical methods taking account of sampling strategy	
		(*e*) Describe any sensitivity analyses	6
**Results**			
Participants	13*	(a) Report numbers of individuals at each stage of study—eg numbers potentially eligible, examined for eligibility, confirmed eligible, included in the study, completing follow-up, and analysed	Not applicable
		(b) Give reasons for non-participation at each stage	Not applicable
		(c) Consider use of a flow diagram	Not applicable
Descriptive data	14*	(a) Give characteristics of study participants (eg demographic, clinical, social) and information on exposures and potential confounders	7-10
		(b) Indicate number of participants with missing data for each variable of interest	Not applicable
		(c) *Cohort study*—Summarise follow-up time (eg, average and total amount)	Not applicable
Outcome data	15*	*Cohort study*—Report numbers of outcome events or summary measures over time	Not applicable
		Case-control study—Report numbers in each exposure category, or summary measures of exposure	Not applicable
		Cross-sectional study—Report numbers of outcome events or summary measures	7-10
Main results	16	(*a*) Give unadjusted estimates and, if applicable, confounder-adjusted estimates and their precision (eg, 95% confidence interval). Make clear which confounders were adjusted for and why they were included	7-10
		(*b*) Report category boundaries when continuous variables were categorized	Not applicable
		(*c*) If relevant, consider translating estimates of relative risk into absolute risk for a meaningful time period	Not applicable
Other analyses	17	Report other analyses done—eg analyses of subgroups and interactions, and sensitivity analyses	7-10
**Discussion**			
Key results	18	Summarise key results with reference to study objectives	11-12
Limitations	19	Discuss limitations of the study, taking into account sources of potential bias or imprecision. Discuss both direction and magnitude of any potential bias	11-12
Interpretation	20	Give a cautious overall interpretation of results considering objectives, limitations, multiplicity of analyses, results from similar studies, and other relevant evidence	11-12
Generalisability	21	Discuss the generalisability (external validity) of the study results	12
**Other information**			
Funding	22	Give the source of funding and the role of the funders for the present study and, if applicable, for the original study on which the present article is based	13

*Give information separately for cases and controls in case-control studies and, if applicable, for exposed and unexposed groups in cohort and cross-sectional studies.

Note: An Explanation and Elaboration article discusses each checklist item and gives methodological background and published examples of transparent reporting. The STROBE checklist is best used in conjunction with this article (freely available on the Web sites of PLoS Medicine at http://www.plosmedicine.org/, Annals of Internal Medicine at http://www.annals.org/, and Epidemiology at http://www.epidem.com/). Information on the STROBE Initiative is available at www.strobe-statement.org.

For peer review only

BMJ Open

Sensitivity analysis for mistakenly adjusting for mediators in estimating total effect from the perspective of causal diagrams

Journal:	*BMJ Open*
Manuscript ID	bmjopen-2016-015640.R1
Article Type:	Research
Date Submitted by the Author:	08-Mar-2017
Complete List of Authors:	Wang, Tingting; Shandong University, Department of Epidemiology and Biostatistics<br>Li, Hongkai; Shandong University, Department of Epidemiology and Biostatistics<br>Su, Ping; Shandong University, Department of Epidemiology and Biostatistics<br>Yu, Yuanyuan; Shandong University, Department of Epidemiology and Biostatistics<br>Sun, Xiaoru; Shandong University, Department of Epidemiology and Biostatistics<br>Liu, Yi; Shandong University, Department of Epidemiology and Biostatistics<br>Yuan, Zhongshang; Shandong University, Department of Epidemiology and Biostatistics<br>Xue, Fuzhong; Shandong University, Department of Epidemiology and Biostatistics
**Primary Subject Heading**:	Epidemiology
Secondary Subject Heading:	Public health
Keywords:	EPIDEMIOLOGY, PUBLIC HEALTH, STATISTICS & RESEARCH METHODS

SCHOLARONE™
Manuscripts

For peer review only - http://bmjopen.bmj.com/site/about/guidelines.xhtml

Sensitivity analysis for mistakenly adjusting for mediators in estimating total effect from the perspective of causal diagrams

Tingting Wang†, Hongkai Li†, Ping Su, Yuanyuan Yu, Xiaoru Sun, Yi Liu, Zhongshang Yuan, Fuzhong Xue*

Department of Biostatistics, School of Public Health, Shandong University, Jinan City, Shandong Province, P.R.China

Email addresses:

TTW: wangtingting161780@163.com

HKL: lihongkaiyouxiang@163.com

PS: sdsuping@163.com

YYY: yu_yy_1993@163.com

XRS: sunny150139@126.com

YL: liuyi238@sdu.edu.cn

ZSY: yuanzhongshang@sdu.edu.cn

*Corresponding Author

xuefzh@sdu.edu.cn (FZX)

Word Count: 3033

Abstract

Objectives: In observational studies, epidemiologists often attempt to estimate the total effect of exposure on outcome of interest. However, when the underlying diagram is unknown and only limited knowledge is available, it is necessary to dissect bias performances in mistakenly adjusting for mediators under logistic regression in estimating the total effect of exposure on outcome. Through simulation, we focus on six causal diagrams concerning different roles of mediators. Sensitivity analysis was conducted to assess the bias performances of varying across the effects of exposure-mediator and mediator-outcome in adjusting for mediator under the framework of logistic regression model.

Setting: Based on the causal relationships in real world, we compare the bias of varying across the effect of exposure-mediator with the one of varying across the effect of mediator-outcome in adjusting for mediator. The magnitude of the bias was

For peer review only

defined by the difference between the estimated effect using logistic regression and the total effect of the exposure on the outcome.

Results: In the following four scenarios: a single mediator, two series mediators, two independent parallel mediators or two correlated parallel mediators, the bias of varying across the effect of exposure-mediator was greater than the one of varying across the effect mediator-outcome in adjusting for the mediator. While in other two scenarios: a single mediator or two independent parallel mediators in the presence of unobserved confounders, the bias of varying across the effect of exposure-mediator was less than the one of varying across the effect mediator-outcome in adjusting for the mediator.

Conclusions: The biases were higher sensitive to the variation of effects of exposure-mediator than effects of mediator-outcome in adjusting for mediator in the absence of unobserved confounders; while the biases were higher sensitive to the variation of effects of mediator-outcome than effects of exposure-mediator in the presence of unobserved confounder.

Strengths and limitations of this study

1) For six different causal diagrams, we compared biases of distinct adjustment strategies with and without adjusting for mediators by conducting simulation studies.

2) Sensitivity analysis was conducted to assess the performances of varying across the effects of exposure-mediator and mediator-outcome.

3) The simulation schemes and parameters were conducted mainly based on real observational studies.

4) Combination of theoretical derivation and simulation studies make the results more credible.

For peer review only - http://bmjopen.bmj.com/site/about/guidelines.xhtml

5) The limitation of simulation studies was under the framework of logistic regression and only focused on binary variables.

Introduction

Estimating the total effects of the exposure (E) on the outcome (D) is a great challenge in epidemiology studies, because confounders are commonly confused with mediators.[1-3] If confounders and mediators are misclassified, the ability to control confounder in the estimation of the total effect of the exposure on the outcome is hampered. Causal diagrams provides a formal conceptual framework to identify and select confounders,[4-5] so that it can avoid falling into analytic pitfalls.[6] In practice, even the underlying causal diagrams and the role of covariates (mediator, confounder, collider and instrumental variable) are not all learned, investigators usually adjusted for the covariates that are associated with the outcome and exposure.[7-10] Therefore, our paper focuses on the bias of varying across the effects of exposure-mediator ($E \rightarrow M$) and mediator-outcome ($M \rightarrow D$) in mistakenly adjusting for mediators under logistic regression model.

The causal inference literature has made a considerable contribution to mediation analysis by providing definitions for direct and indirect effects that allow for the effect decomposition of a total effect into a direct and an indirect effect.[11-19] Arbitrarily adjusting for a mediator would generally lead to biased estimate of the total effect of the exposure on the outcome.[6, 20-21] Nevertheless, in the perspective of causal diagrams, little attention was paid to the biases in adjusting for mediators under the logistic regression model in estimating the total effect of E on D. Hence, we focused on the sensitivity analysis technique to assess the effects $E \rightarrow M$ and $M \rightarrow D$ in adjusting for mediator.

For peer review only - http://bmjopen.bmj.com/site/about/guidelines.xhtml

In this paper, six typical causal diagrams corresponding to causal correlation are given in Figure 1: a single mediator M (Figure 1a); two series mediators (Figure 1b); two independent parallel mediators (Figure 1c); two correlated parallel mediators (Figure 1d); a single mediator with an unobserved confounder (Figure 1e); two parallel mediators with an unobserved confounder (Figure 1f). The paper aim to explore the sensitivity of bias to the variation of the effects of $E \rightarrow D$ and $M \rightarrow D$ in adjusting for mediator. Hence, both theoretical proofs and quantitative simulations were performed to dissect the bias of varying across the effect of $E \rightarrow M$ and the one of varying across the effect of $M \rightarrow D$ in adjusting for mediators under logistic model.

Methods

A directed acyclic graph (*DAG*) is composed of variables (nodes) and arrows (directed edges) between nodes such that the graph is acyclic. Pearl formalized causal diagrams as directed acyclic graphs (*DAG*s), providing investigators with powerful tools for bias assessment.[22] The causal directed acyclic graph theory provides a device for deducing the statistical associations implied by causal relations. Furthermore, given a set of observed statistical associations, a researcher armed with causal diagrams theory can systematically characterize all causal structures compatible with the observations.[23-24]

The total effect can be calculated based on the *do-calculus* and *back-door* criterion proposed by Judea Pearl.[25-26] For exposure X and outcome Y, a set of variables Z is said to satisfy the backdoor path criterion with respect to (X, Y) if no variable in Z is a descendant of X and if Z blocks all back-door paths from X to Y. Then the effect of X on Y is given by the formula,

For peer review only - http://bmjopen.bmj.com/site/about/guidelines.xhtml

$$P(y \mid do(x)) = \sum_{Z} P(y \mid x, z) P(z)$$

Note that the expression on the right hand side of the equation is simply a standardized mean. The difference $E(Y \mid do(x')) - E(Y \mid do(x''))$ is taken as the definition of "causal effect", where x' and x'' are two distinct realizations of X.[21] The interventional distribution, such as that corresponding to $Y(x)$, namely $P(y|do(x))$, is not necessarily equal to a conditional distribution $P(y \mid x)$. It stands for the probability of $Y = y$ when the exposure X set to level x. The ignorability assumption $Y(x) \perp X$ states that if we happen to have information on the exposure variable, it does not give us any information about the outcome Y after the intervention $do(x)$ was performed. Besides it can be shown that if ignorability holds for $Y(x)$ and X (alternatively if there are no back-door paths from X to Y in the corresponding causal DAGs), then $p(y \mid do(x)) = p(y \mid x)$.[27-28]

Let D_e and M_e denote respectively the values of the outcome and mediator that would have been observed had the exposure E been set to level e. On the odds ratio ($OR^{TE}_{E \to D}$) scale, the total effect ($\beta^{TE}_{E \to D} = \log(OR^{TE}_{E \to D})$), comparing exposure level e with e^*, is given by $OR^{TE}_{E \to D} = \dfrac{P(D_e = 1)/\{1 - P(D_e = 1)\}}{P(D_{e^*} = 1)/\{1 - P(D_{e^*} = 1)\}}$.[18-19] While the effect ($\beta_{ED|M}(m)$) of adjusting for mediator M by logistic regression model can be given

$$\begin{aligned}\beta_{ED|M}(m) &= \operatorname{logit}\{P(D = 1 \mid e = 1, m)\} - \operatorname{logit}\{P(D = 1 \mid e^* = 0, m)\} \\ &= \log\left\{\frac{P(D = 1 \mid e = 1, m) P(D = 0 \mid e^* = 0, m)}{P(D = 0 \mid e = 1, m) P(D = 1 \mid e^* = 0, m)}\right\}\end{aligned}$$

where $P(D = 1 \mid e, m)$ denotes the probability of $D = 1$ when the exposure E, and mediator M, have been set to level e, and m, respectively. Taking Figure 1a as an example, the logistic regression is

$$\operatorname{logit}\{P(D = 1 \mid e, m)\} = \alpha_1 + \beta_0 e + \beta_2 m.$$

For peer review only - http://bmjopen.bmj.com/site/about/guidelines.xhtml

Therefore, the total effect ($\beta^{TE}_{E\to D}$) of exposure E on outcome D on the scale of logarithm odds ratio was equal to

$$\begin{aligned}\beta^{TE}_{E\to D} &= \log(OR^{TE}_{E\to D})\\ &= \log\left\{\frac{P(D_e=1)/\{1-P(D_e=1)\}}{P(D_{e*}=1)/\{1-P(D_{e*}=1)\}}\right\}\\ &= \text{logit}\{P(D_e=1)\}-\text{logit}\{P(D_{e*}=1)\}\\ &= \text{logit}\{P(D=1\,|\,e=1)\}-\text{logit}\{P(D=1\,|\,e^*=0)\}\\ &= \text{logit}\left\{\sum_m P(D=1\,|\,e=1,m)P(m\,|\,e=1)\right\}-\text{logit}\left\{\sum_m P(D=1\,|\,e^*=0,m)P(m\,|\,e^*=0)\right\}\end{aligned}$$

The effect estimation ($\hat{\beta}_{ED|M}(m)$) of adjusting for mediator M by logistic regression model was equal to

$$\hat{\beta}_{ED|M}(m)=\text{logit}\left\{\hat{P}(D=1\,|\,e=1,m)\right\}-\text{logit}\left\{\hat{P}(D=1\,|\,e^*=0,m)\right\}$$

where $\hat{P}(D=1\,|\,e=1,m)$ denotes the probability of $D=1$ when the exposure E, and mediator M, have been set to level $e=1$, and m, respectively. And $\hat{P}(D=1\,|\,e^*=0,m)$ denotes the probability of $D=1$ when the exposure E, and mediator M, have been set to level $e^*=0$, and m, respectively.

Note that the bias was defined by taking a difference between effect estimation by adjusting for mediator using logistic regression and the total effect of exposure E on outcome D i.e. $bias=E[\hat{\beta}_{ED|M}(m)]-\beta^{TE}_{E\to D}$. We dissected the biases behavior by varying across the effects $E\to M$ and $M\to D$ in mistakenly adjusting for mediator under the framework of logistic regression model.

Simulation

As shown in Figure 1, six scenarios are designed to dissect bias behaviors of mistakenly adjusting for mediators using logistic regression model. We made the following assumptions for the simulation: 1) all variables were binary following a Bernoulli distribution; 2) the effect from parent nodes to their child node were

For peer review only - http://bmjopen.bmj.com/site/about/guidelines.xhtml

positive and log-linearly additive. Taking Figure 1a as an example, we randomly generated the exposure following a Bernoulli distribution (i.e. let $P(e=1)=\pi$), then, $P_M=\exp(\alpha_0+\beta_1 e)/\{1+\exp(\alpha_0+\beta_1 e)\}$ for calculating the distribution probability of child node *M* from its parent node *E*. Similarly, $P_D=\exp(\alpha_1+\beta_0 e+\beta_2 m)/\{1+\exp(\alpha_1+\beta_0 e+\beta_2 m)\}$ generated the distribution probability of *D*, where the parameters α_0 and α_1 denoted the intercept of *M* and *D* respectively, and effect parameter β_0, β_1, β_2 referred to the effects of the parent node on their corresponding child node using log odds ratio scale.

After generating data, we dissected the biases behavior between the effects of *E*→*M* and *M*→*D* in mistakenly adjusting for mediator under logistic regression model. In scenario 1 (Figure 1a), we compared the performances by across varying the effects of *E*→*M* and *M*→*D*. Similarly, in scenario 2 (Figure 1b), the effects of $E \to M_1$, $M_1 \to M_2$ and $M_2 \to D$ were explored. In scenario 3 (Figure 1c), we dissected the effects of $E \to M_1$ ($E \to M_2$) and $M_1 \to D$ ($M_2 \to D$). The comparison of scenario 4 (Figure 1d) was the same as scenario 3 (Figure 1c). In scenario 5 (Figure 1e), the effects of *E*→*M* and *M*→*D* were excavated. The scenario 6 (Figure 1f) was identical to the scenario 3. We explored the biases in adjusting for mediator under logistic regression model and thus identified the sensitivity of bias to the variation of the effects of exposure-mediator and mediator-outcome.

For each of the 6 simulation scenarios, we observed bias performances of varying across distinct effects in adjusting for mediator using logistic regression model with 1000 simulations repetitions. All simulations were conducted using software R from CRAN (http://cran.r-project.org/).

For peer review only - http://bmjopen.bmj.com/site/about/guidelines.xhtml

Results

Scenario 1: one single mediator (Figure 1a)

In Figure 1(a) of the simplest case, E has a direct ($E \rightarrow D$) effect and an indirect ($E \rightarrow M \rightarrow D$) effect on D. Figure 2A depicted that the bias of varying across the effect of $E \rightarrow M$ was obviously greater than the bias of varying across the effect of $M \rightarrow D$. That is, the sensitivity of bias to the variation of the effect $E \rightarrow M$ was greater than the effect of $M \rightarrow D$ in adjusting for the mediator M using logistic regression model. In particular, if the effect of $E \rightarrow M$ was specified to zero in Figure 2B, M became an independent cause of the outcome, and in this case adjusting for M obtained a positive bias. Moreover, Figure 2 indicated that adjusting for mediator M was indeed biased to the total effect of the exposure on the outcome.

The total effect ($\beta^{TE}_{E \rightarrow D}$) of exposure E on outcome D on the scale of logarithm odds ratio was equal to

$$\begin{aligned}
\beta^{TE}_{E \rightarrow D} = \log(OR^{TE}_{E \rightarrow D}) &= \log\left\{\frac{P(D_e = 1)/\{1-P(D_e = 1)\}}{P(D_{e*} = 1)/\{1-P(D_{e*} = 1)\}}\right\} \\
&= \log\left\{\frac{P(D_e = 1)\times\{1-P(D_{e*} = 1)\}}{\{1-P(D_e = 1)\}\times P(D_{e*} = 1)}\right\} \\
&= \log\left\{\frac{P(D=1 \mid e=1)\times P(D=0 \mid e^*=0)}{P(D=0 \mid e=1)\times P(D=1 \mid e^*=0)}\right\} \\
&= \log\left\{\frac{[\sum_m P(D=1 \mid e=1,m)P(m \mid e=1)]\times[\sum_m P(D=0 \mid e^*=0,m)P(m \mid e^*=0)]}{[\sum_m P(D=0 \mid e=1,m)P(m \mid e=1)]\times[\sum_m P(D=1 \mid e^*=0,m)P(m \mid e^*=0)]}\right\}
\end{aligned}$$

The effect ($\beta_{ED|M}(m)$) of adjusting for mediator M by logistic regression model can be given

$$\begin{aligned}
\beta_{ED|M}(m) &= \text{logit}\{P(D=1 \mid e=1,m)\} - \text{logit}\{P(D=1 \mid e^*=0,m)\} \\
&= \log\left\{\frac{P(D=1 \mid e=1,m)\times\{1-P(D=1 \mid e^*=0,m)\}}{\{1-P(D=1 \mid e=1,m)\}\times P(D=1 \mid e^*=0,m)}\right\} \\
&= \beta_0
\end{aligned}$$

For peer review only

For peer review only - http://bmjopen.bmj.com/site/about/guidelines.xhtml

β_0 denotes coefficient of the E adjusting for M using logistic regression model. Furthermore, the effect of adjusting for M was equal to the controlled direct effect.[19] Therefore, the bias of adjusting for mediator using logistic regression model could be obtained i.e. $bias = \beta_{ED|M}(m) - \beta_{E \to D}^{TE}$. We added signs to the edges of the directed acyclic graph to indicate the presence of a particular positive or negative effect in the Figure 3. Therefore, we gained $bias < 0$ under the condition of $\beta_1 * \beta_2 > 0$ (the effect $E \to M$ β_1 and the effect $M \to D$ β_2), indicating that the total effect of E on D was biased in adjusting for M using logistic regression model in Figure 3a, Figure 3b, Figure 3e & Figure 3f. And the bias was less than zero when the effect $E \to M$ (β_1) and the effect $M \to D$ (β_2) share same signs. (i.e. the effects $E \to M$ ($\beta_1 > 0$) and $M \to D$ ($\beta_2 > 0$) were a positive sign or the effects $E \to M$ ($\beta_1 < 0$) and $M \to D$ were a negative sign ($\beta_2 < 0$)). Furthermore, we obtained $bias > 0$, if $\beta_1 * \beta_2 < 0$, suggesting that the total effect of E on D was biased in adjusting for M in Figure 3c, Figure 3d, Figure 3g & Figure 3h. And the bias was greater than zero when the signs of the effects $E \to M$ (β_1) and $M \to D$ (β_2) were the opposite. The results illustrated that the bias was less than zero under the case of the effects of exposure-mediator and mediator-outcome sharing the same sign; the bias was greater than zero under circumstances of the effects of exposure-mediator and mediator-outcome having opposite signs. The more details of theoretical derivation have been put in Appendix.

Scenario 2: two series mediators (Figure 1b)

Figure 1(b) is a depiction through two series mediators, decomposing total effects into direct ($E \to D$) and indirect ($E \to M_1 \to M_2 \to D$) components. The bias of varying across

For peer review only - http://bmjopen.bmj.com/site/about/guidelines.xhtml

the effect of $E \rightarrow M_1$ was greater than the one of varying across the effect of $M_2 \rightarrow D$ under adjustment for M_1, adjustment for M_2 and adjustment for M_1 M_2 in Figure 4, respectively. In this situation, the correlation of series mediators was strong enough to avoid M_2 from becoming an independent cause of the outcome.

Scenario 3: two independent parallel mediators (Figure 1c)

Figure 1c shows that the exposure E independently causes M_1 and M_2 and indirectly influences the outcome D through M_1 and M_2, forming three causal paths $E \rightarrow D$, $E \rightarrow M_1 \rightarrow D$ and $E \rightarrow M_2 \rightarrow D$. We obtained that the bias of varying across the effect of $E \rightarrow M_1$ was considerably greater than the one of varying across the effect of $M_1 \rightarrow D$ under adjustment for M_1 in Figure 5A. However, the bias of varying across the effect of $E \rightarrow M_2$ was nearly equal to the one with varying across the effect of $M_2 \rightarrow D$ under the identical adjustment for M_1 in Figure 5A. Then, an above similar result can be obtained in Figure 5B. In addition, Figure 5C indicated that biases of varying across the effects of $E \rightarrow M_1$ and $E \rightarrow M_2$ were obviously greater than the one of varying across the effects of $M_1 \rightarrow D$ and $M_2 \rightarrow D$ under adjustment for M_1 M_2.

Scenario 4: two correlated parallel mediators (Figure 1d)

In Figure 1d, there exist five paths from E to D: $E \rightarrow D$, $E \rightarrow M_1 \rightarrow D$, $E \rightarrow M_2 \rightarrow D$, $E \rightarrow M_1 \leftarrow M_2 \rightarrow D$ and $E \rightarrow M_2 \rightarrow M_1 \rightarrow D$. In particular, the path $E \rightarrow M_1 \leftarrow M_2 \rightarrow D$ is a blocked path, due to the M_1 being a collider node. Figure 6A indicated that the bias of varying across the effect of $E \rightarrow M_1$ was obviously greater than the one of varying across the effect of $M_1 \rightarrow D$ under the adjustment for M_1. However, the bias of varying across the effect of $E \rightarrow M_2$ was almost equal to the one of varying across the effect of $M_2 \rightarrow D$ under the identical adjustment model. Similarly, Figure 6B showed an

For peer review only - http://bmjopen.bmj.com/site/about/guidelines.xhtml

analogous result of biases behavior. Besides, Figure 6C manifested that biases of varying across the effects of $E \rightarrow M_1$ and $E \rightarrow M_2$ were greater than the ones of varying across the effects of $M_1 \rightarrow D$ and $M_2 \rightarrow D$ in adjusting for M_1 and M_2. Simultaneously, the bias was higher sensitive to the variation of effect of $E \rightarrow M_2$ than effects of $E \rightarrow M_1$ under the identical model, which adjustment for the collider node M_1 would partially open the path $E \rightarrow M_1 \leftarrow M_2 \rightarrow D$.

Scenario 5: a single mediator with an unobserved confounder (Figure 1e)

Figure 1e provides a causal diagram representing the relationship among exposure E, outcome D, mediator M and unobserved confounder U. It revealed that the bias of varying across the effect of $E \rightarrow M$ was lower than the one of varying across the effect of $M \rightarrow D$. Unobserved confounder distorts the association between the exposure and outcome ($E \leftarrow U \rightarrow D$) in Figure 7.

Scenario 6: two parallel mediators with an unobserved confounder (Figure 1f)

As described above, Figure 1f is a depiction of two parallel mediators M_1 and M_2 with an unobserved confounder U. Figure 8A indicated that the bias of varying across the effect of $E \rightarrow M_1$ was obviously less than the one of varying across the effect of $M_1 \rightarrow D$ under the adjustment for M_1. However, the bias of varying across the effect of $E \rightarrow M_2$ was greater than the bias of varying across the effect of $M_2 \rightarrow D$ under the identical model of adjusting for M_1. A similar result can also obtain in Figure 8B. Besides, biases of varying across the effects of $E \rightarrow M_1$ and t $E \rightarrow M_2$ were distinctly less than the ones of varying across the effects of $M_1 \rightarrow D$ and $M_2 \rightarrow D$ under the common model of adjusting for M_1 and M_2 in Figure 8C.

Discussion

For peer review only - http://bmjopen.bmj.com/site/about/guidelines.xhtml

In the paper, we dissected the sensitivity of bias to the variation of the effects of exposure-mediator and mediator-outcome in adjusting for mediators under the framework of logistic regression model. In the following four scenarios: a single mediator (Figure 1a in scenario 1), two series mediators (Figure 1b in scenario 2), two independent parallel (Figure 1c in scenario 3) or two correlated parallel mediators (Figure 1d in scenario 4), the bias of varying across the effect of exposure-mediator was greater than the one of varying across the effect mediator-outcome in adjusting for the mediator (Figure 2, Figure 4, Figure 5 & Figure 6). However, in other two scenarios: a single mediator or two independent parallel mediators in the presence of unobserved confounders (Figure 1e in scenario 5 & Figure 1f in scenario 6), the biases were higher sensitive to the variation of effect of mediator-outcome than effects of exposure-mediator in adjusting for mediator (Figure 7 & Figure 8).

Conditioning on a mediator is of concern in all areas of epidemiologic researches,[11,17,29] it indeed led to bias in estimating the total effect of the exposure on the outcome.[6, 20-21] Mediators and confounders were indistinguishable in terms of statistical association and conceptual grounds.[3] Most of the studies focused on the mediation effect analysis such as the calculation of direct effects and indirect effect.[18-19,30-33] Little effort has been made to learn the biases performances in adjusting for mediator in estimating the total effect of exposure on outcome. Our study results revealed that the biases were higher sensitive to the variation of effect of exposure-mediator than effects of mediator-outcome in adjusting for mediator in the absence of the unobserved confounder in causal diagrams (Figure 1a, Figure 1b, Figure 1c & Figure 1d). Nevertheless, for causal diagrams (Figure 1e & Figure 1f), the biases were higher sensitive to the variation of effect of mediator-outcome than effects of exposure-mediator in adjusting for mediator in the presence of the

For peer review only - http://bmjopen.bmj.com/site/about/guidelines.xhtml

unobserved confounder. Therefore, the biases of varying across different effects depended on the causal diagrams framework whether there existed unobserved confounder.

We need note that, our simulation study was not comprehensive enough to evaluate the bias performances in adjusting for the mediator under logistic regression, because it only considered binary variables, the certain scenarios of effect size and the common type of models. The work in the further ought to reinforce the mechanisms and conceptual frameworks of confounder and mediator form causal diagrams so as to avoid falling into analytic pitfalls.

Conclusion

In conclusion, we showed that the sensitivity of bias to the variation of the effects of exposure-mediator and mediator-outcome was related to whether there is an unobserved confounder in causal diagrams. The biases were higher sensitive to the variation of effects of exposure-mediator than effects of mediator-outcome in adjusting for mediator in the absence of unobserved confounders; while the biases were higher sensitive to the variation of effects of mediator-outcome than effects of exposure-mediator in the presence of unobserved confounder.

Statements

Ethics approval and materials

Not applicable

Competing interests

The authors declare that they have no competing interests.

Funding

This work was supported by grants from the National Natural Science Foundation of

For peer review only - http://bmjopen.bmj.com/site/about/guidelines.xhtml

China (grant number 81573259)

Provenance and peer review

Not commissioned; externally peer reviewed.

Data sharing statement

No additional data are available.

Authors' contributions

TTW and HKL jointly conceived the idea behind the article and designed the study. TTW helped conduct the literature review, performed the simulation and prepared the first draft of the manuscript. PS, YYY, XRS, YL and ZSY participated in the design of the study and the revision of the manuscript. FZX advised on critical revision of the manuscript for important intellectual content. All authors read and approved the final manuscript.

Acknowledgments

We would like to thank the anonymous reviewers and academic editors for providing us with constructive comments and suggestions and also wish to acknowledge our colleagues for their invaluable work.

Open Access

This is an Open Access article distributed in accordance with the Creative Commons Attribution Non Commercial (CC BY-NC 4.0) license, which permits others to distribute, remix, adapt, build upon this work noncommercially, and license their derivative works on different terms, provided the original work is properly cited and the use is non-commercial. See: http:// creativecommons.org/licenses/by-nc/4.0/

References

1 Bulterys M, Morgenstern H. Confounding or intermediate effect? An appraisal of iatrogenic bias in perinatal AIDS research. *Paediatr Perinat Epidemiol* 1993;7:387–394.

2 Herna´n MA, Hernandez-Diaz S, Werler MM, et al. Causal knowledge as a prerequisite for confounding evaluation: an application to birth defects epidemiology. *Am J Epidemiol* 2002;155:176–184.

3 MacKinnon DP, Krull JL, and Lockwood CM. Equivalence of the mediation,

For peer review only - http://bmjopen.bmj.com/site/about/guidelines.xhtml

confounding and suppression effect. *Prevention Science* 2000;1:173–181.
4 Howards PP, Schisterman EF, Poole C, et al. “Toward a clearer definition of confounding” revisited with directed acyclic graphs, *Am J Epidemiol* 2012;176:506–511.
5 Williamson EJ, Aitken Z, Lawrie J, et al. Introduction to causal diagrams for confounder selection. *Respirology*. 2014;19:303-311.
6 Wil cox AJ, Weinberg CR, Basso O. On the pitfalls of adjusting for gestational age at birth. *Am J Epidemiol*. 2011;174:1062–1068.
7 Psaty BM, Koepsell TD, Lin D, et al. Assessment and control for confounding by indication in observational studies. *J Am Geriatr Soc* 1999; 47:749-54.
8 Tilaki K H. Methodological issues of confounding in analytical epidemiology studies. *Caspian J intern Med* 2012;3:488-495.
9 Kamangar F MD. Confounding variable in epidemiology studies: Basics and beyond. *Arch Tran Med*. 2012;15:508-516.
10 Gaskins AJ, Schisterman EF. The effect of lipid adjustment on the analysis of environmental contaminants and the outcome of human health risks. *Methods Mol Biol* 2009;580:371-381.
11 Robins JM, Greenland S. Identifiability and exchangeability for direct and indirect effects. *Epidemiology* 1992;3:143–155.
12 Pearl J. Direct and indirect effects. Seventeenth conference of uncertainy in artificial intelligence, 2001. San Francisco, CA: Morgan Kaufmann, 2001.
13 Huang B, Sivaganesan S, Succop P, et al. Statistical assessment of mediational effects for logistic mediational models. *Statist. Med* 2004;23:2713-2728.
14 Valeri L, Coull B A. Estimating causal contrasts involving intermediate variables in the presence of selection bias [J]. *Statistics in Medicine*, 2016, 35(26): 4779-4793.
15 MacKinnon D P, Pirlott A G. Statistical approaches for enhancing causal interpretation of the M to Y relation in mediation analysis [J]. *Personality and Social Psychology Review*, 2015, 19(1): 30-43.
16 Imai K, Keele L, Tingley D. A general approach to causal mediation analysis. *Psychol Methods*. 2010;15(4):309–334.
17 VanderWeele TJ. Bias formulas for sensitivity analysis for direct and indirect effects. *Epidemiology*. 2010;21(4):540–551.
18 VanderWeele TJ, Vansteelandt S. Odds ratios for mediation analysis for a dichotomous outcome. *Am J Epidemiol*. 2010;172(12):1339–1348.
19 Valeri L, VanderWeele TJ. Mediation analysis allowing for exposure-mediator interactions and causal interpretation: theoretical assumptions and implementation with SAS and SPSS macros [J]. *Psychological methods*, 2013, 18(2): 137.
20 Robins J. The control of confounding by intermediate variable. *Statistics in Medicine*. 1989;8:679-701.
21 Schistermana EF, Coleb SR, Platt RW. Overadjustment Bias and Unnecessary Adjustment in Epidemiologic Studies. *Epidemiology*. 2009;20:488–495.
22 Pearl J. Causal diagrams for empirical research. *Biometrika*. 1995;82:669-688.

For peer review only - http://bmjopen.bmj.com/site/about/guidelines.xhtml

23 Pearl J. Causality: models, reasoning, and inference. *Econometric Theory*. 2003;19: 675-685.

24 Joffe M, Gambhir M, Chadeau-Hyam M, et al. Causal diagrams in systems epidemiology. *Emerg Themes Epidemiol*. 2012; 9:1-18.

25 Pearl, J. Causality: models, reasoning, and inference. Cambridge: Cambridge University Press; 2000.

26 Pearl J. Causal inference in statistics: an overview. *Statistics Surveys*. 2009; 3:96–146.

27 Rubin DB. Should observational studies be designed to allow lack of balance in covariate distributions across treatment groups? *Statistics in Medicine*. 2009;28:1420-1423.

28 Shpitser I. Counterfactual graphical models for longitudinal mediation analysis with unobserved confounding. *Cognitive Science* 2013;37:1011-1035.

29 VanderWeele TJ, Mumford SL, Schisterman EF. Conditioning on intermediates in perinatal epidemiology. *Epidemiology*. 2012;23(1):1–9.

30 Lange T, Rasmussen M, Thygesen LC. Assessing natural direct and indirect effects through multiple pathways. *American Journal of Epidemiology*. 2013;179, 513–518.

31 Shpitser, Ilya and VanderWeele, Tyler J. "A complete graphical criterion for the adjustment formula in mediation analysis," *The International Journal of Biostatistics*.2011;7,1-24.

32 Albert J M, Wang W. Sensitivity analyses for parametric causal mediation effect estimation [J]. *Biostatistics*, 2015, 16(2): 339-351.

33 Wang W, Zhang B. Assessing natural direct and indirect effects for a continuous exposure and a dichotomous outcome[J]. *Journal of Statistical Theory and Practice*, 2016, 10(3): 574-587.

Figure 1: Six causal diagrams were designed for estimating the causal effect of E on D. a) a single mediator M; b) two series mediators M_1 and M_2; c) two independent parallel mediators M_1 and M_2; d) two correlated parallel mediators M_1 and M_2; e) a single mediator with an unobserved confounder U; f) two independent parallel mediators M_1 and M_2 with an unobserved confounder U.

Figure 2: The biases with the effects $E \rightarrow M$ (red) and $M \rightarrow D$ (blue) increasing, respectively. Comparison of the bias of different effects in adjustment mediator. The OR of target effect (e.g. $E \rightarrow M$) from 1 to 10 given other effects fixed ln2 in Figure 2A. The OR of the effect $M \rightarrow D$ from 1 to 10 with the effect $E \rightarrow M$ being equal to zero in Figure 2B (Color figure online).

Figure 3: Illustrating the use of positive and negative signs on edges $E \rightarrow M$, $M \rightarrow D$ and $E \rightarrow D$.

Figure 4: The biases with the effects $E \rightarrow M_1$ (red), $M_1 \rightarrow M_2$ (blue) and $M_2 \rightarrow D$ (black) increasing, respectively. Comparison of the bias of different effects in three

For peer review only - http://bmjopen.bmj.com/site/about/guidelines.xhtml

adjustment models: A) adjustment for M_1, B) adjustment for M_2 and C) adjustment for M_1 and M_2. The OR of target effect (e.g. $E \rightarrow M_1$) from 1 to 10 given the effect $M_1 \rightarrow M_2$ fixed ln8 and other effects fixed ln2 in Figure 4 (Color figure online).

Figure 5: The biases with the effects $E \rightarrow M_1$ (red), $E \rightarrow M_2$ (blue), $M_1 \rightarrow D$ (black) and $M_2 \rightarrow D$ (green) increasing, respectively. Comparison of the bias of different effects in three adjustment models: A) adjustment for M_1, B) adjustment for M_2 and C) adjustment for M_1 and M_2. The OR of target effects (e.g. $E \rightarrow M_1$) from 1 to 10 given other edges effects fixed ln2 in Figure 5 (Color figure online).

Figure 6: The biases with the effects $E \rightarrow M_1$ (red), $E \rightarrow M_2$ (blue), $M_1 \rightarrow D$ (black), $M_2 \rightarrow D$ (green) and the effect $M_2 \rightarrow M_1$ (purple) increasing, respectively. Comparison of the bias of different effects in three adjustment models: A) adjustment for M_1, B) adjustment for M_2 and C) adjustment for M_1 and M_2. The OR of target effects (e.g. $E \rightarrow M_1$) from 1 to 10 given other effects fixed ln2 in Figure 6 (Color figure online).

Figure 7: The biases with the effects $E \rightarrow M$ (red) and $M \rightarrow D$ (blue) respectively. Comparison of the bias of different effects in adjustment mediator M. The OR of target effects (e.g. $E \rightarrow M$) from 1 to 10 given the effects of causal edges fixed ln2 and the effect of confounder edges fixed ln5 in Figure ln8 (Color figure online).

Figure 8: The biases with the effects $E \rightarrow M_1$ (red), $E \rightarrow M_2$ (blue), $M_1 \rightarrow D$ (black) and $M_2 \rightarrow D$ (green) respectively. Comparison of the bias of different effects in three adjustment models: A) adjustment for M_1, B) adjustment for M_2, and C) adjustment for M_1 and M_2. The OR of target effects (e.g. $E \rightarrow M_1$) from 1 to 10 given the effects of causal edges fixed ln2 and the effect of confounder edges fixed ln5 in Figure 8 (Color figure online).

For peer review only - http://bmjopen.bmj.com/site/about/guidelines.xhtml

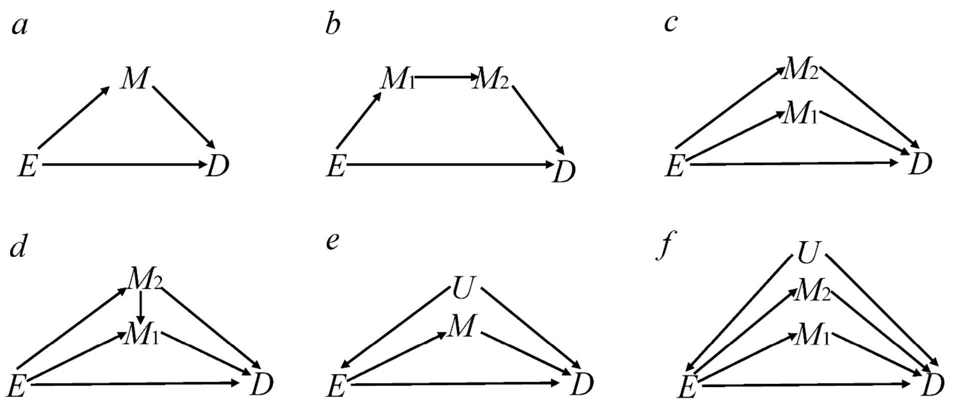

Figure 1: Six causal diagrams were designed for estimating the causal effect of E on‼ ┼ D.

252x110mm (300 x 300 DPI)

er review only

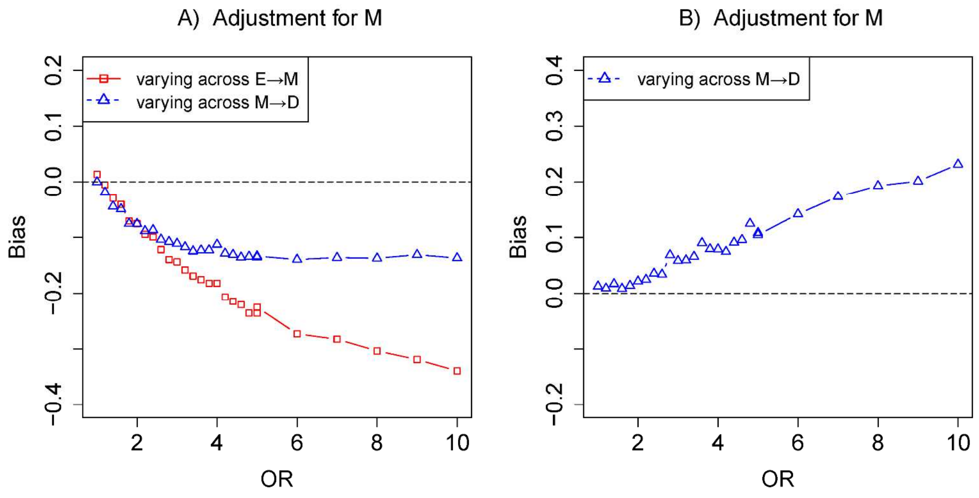

Figure 2 : The biases with the effects E→M (red) and M→D (blue) increasing, respectively.

281x148mm (300 x 300 DPI)

For peer review only

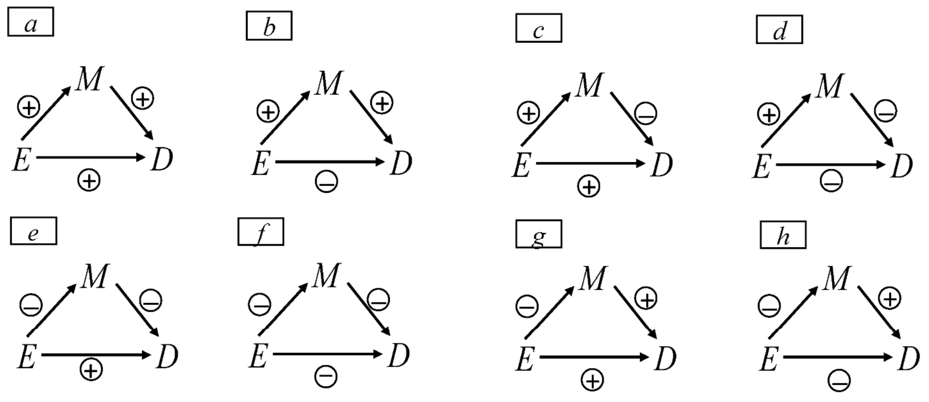

Figure 3: Illustrating the use of positive and negative signs on edges E→M, M→D and E→D.

237x106mm (300 x 300 DPI)

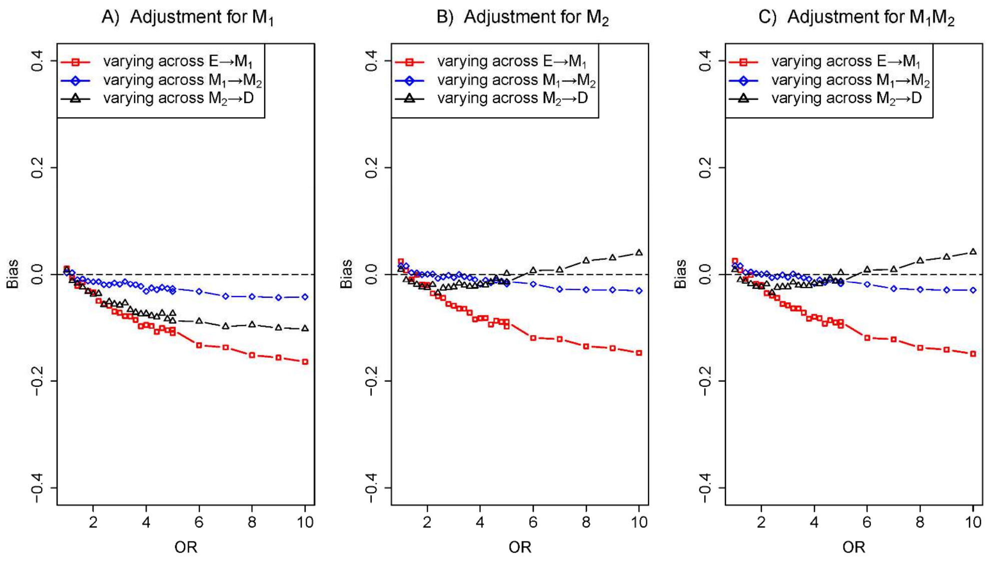

Figure 4: The biases with the effects E→M_1 (red), M_1→M_2 (blue) and M_2→D (black) increasing, respectively.

270x155mm (300 x 300 DPI)

For peer review only

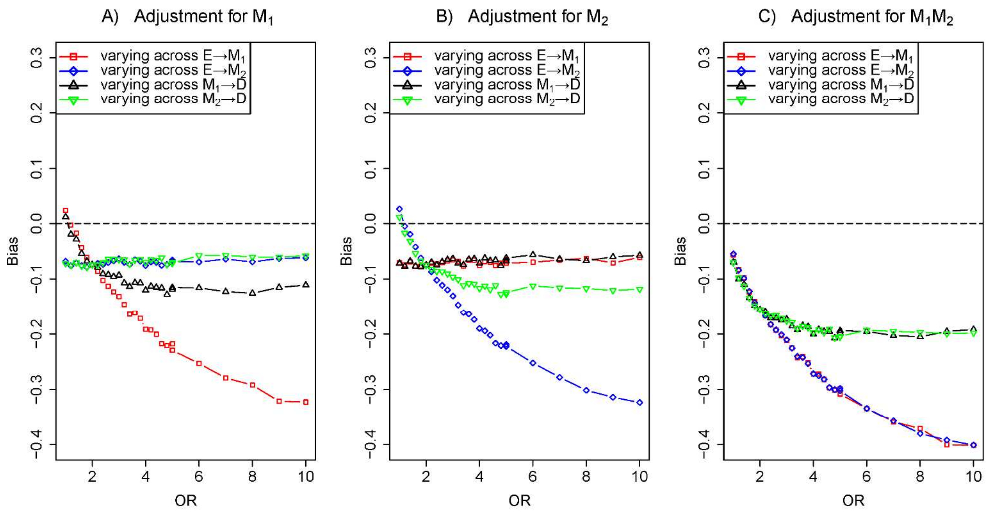

Figure 5 : The biases with the effects E→M_1 (red), E→M_2 (blue), M_1→D (black) and M_2→D (green) increasing, respectively.

279x147mm (300 x 300 DPI)

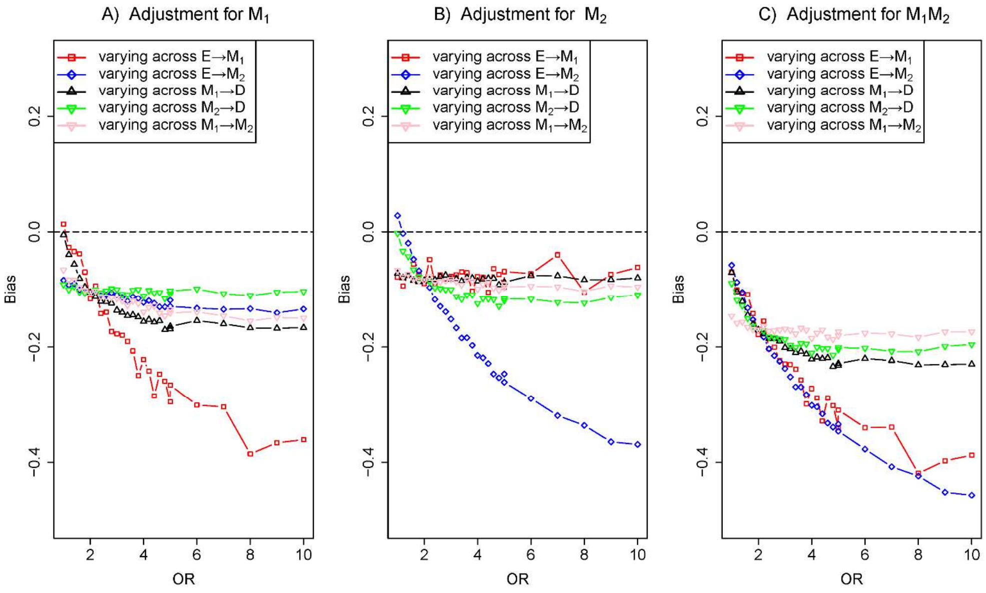

Figure 6: The biases with the effects E→M_1 (red), E→M_2 (blue), M_1 →D (black), M_2 →D (green) and the effect M_2 →M_1 (purple) increasing, respectively.

278x169mm (300 x 300 DPI)

For peer review only

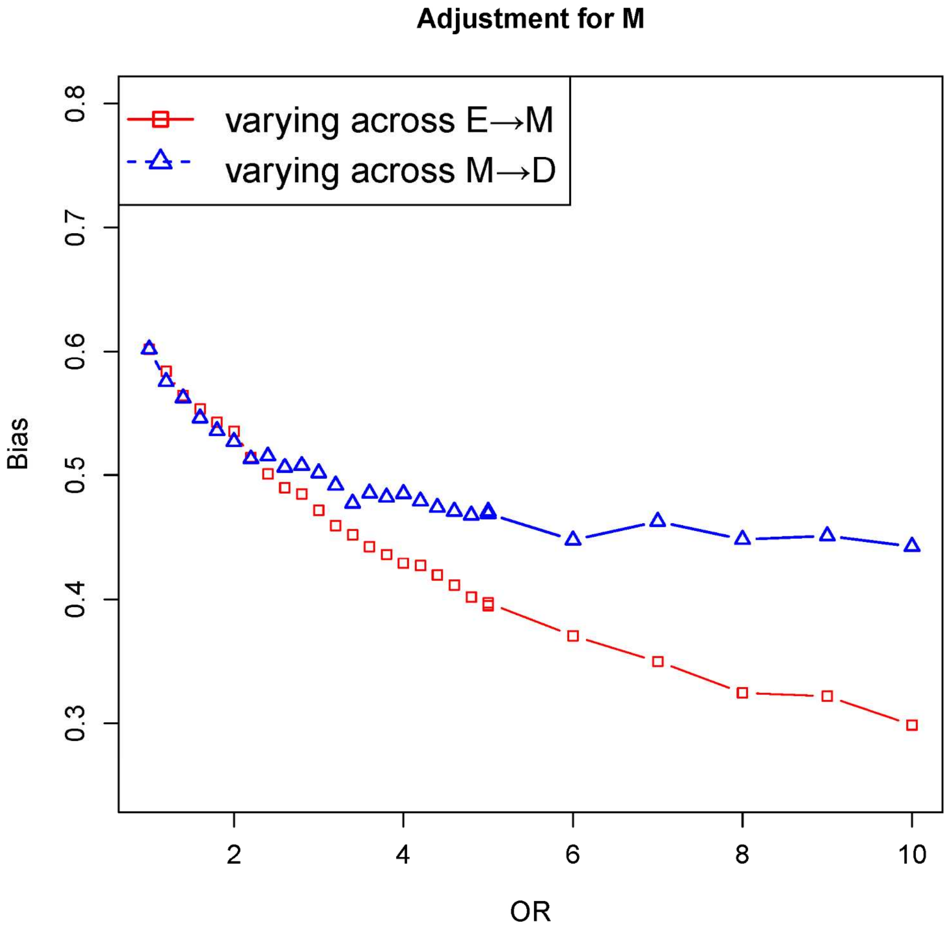

Figure 7: The biases with the effects E→M (red) and M→D (blue) respectively.

177x177mm (300 x 300 DPI)

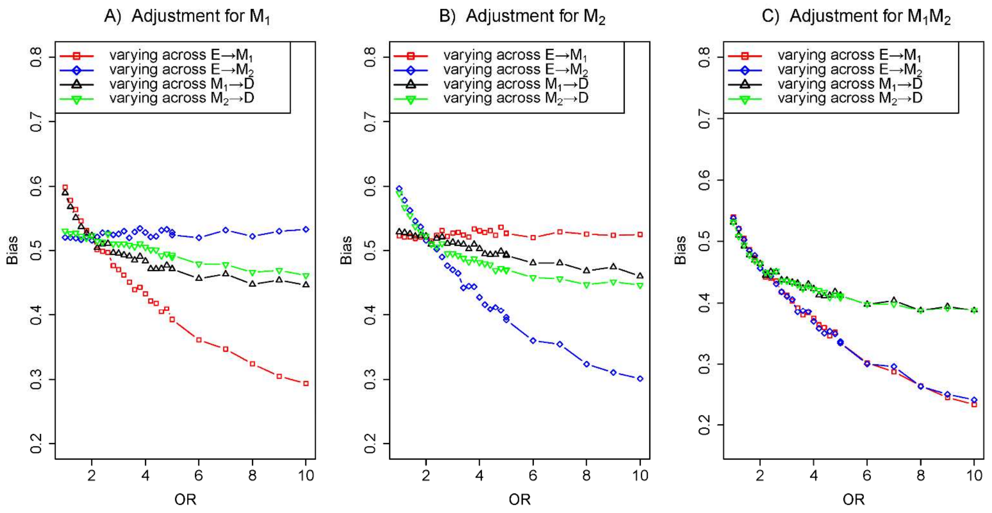

Figure 8 : The biases with the effects E→M_1 (red), E→M_2 (blue), M_1→D (black) and M_2→D (green) respectively.

281x148mm (300 x 300 DPI)

review only

Appendix:

The effect of adjusting for mediator was biased for estimating the total effect of exposure on outcome using logistic regression model. Theoretical derivation of Figure 1a as follow：

Suppose the logistic models among *E*, *M* and *D* are:

$$\text{logit}\{P(D=1|e,m)\} = \alpha_1 + \beta_0 e + \beta_2 m,$$

$$\text{logit}\{P(M=1|e)\} = \alpha_0 + \beta_1 e.$$

The total effect ($\beta^{TE}_{E\rightarrow D}$) of exposure *E* on outcome *D* on the odds ratio ($OR^{TE}_{E\rightarrow D}$) scale was equal to

$$\begin{aligned}\beta^{TE}_{E\rightarrow D} &= \log(OR^{TE}_{E\rightarrow D})\\ &= \log\left\{\frac{P(D_e=1)/\{1-P(D_e=1)\}}{P(D_{e*}=1)/\{1-P(D_{e*}=1)\}}\right\}\\ &= \log\left\{\frac{P(D_e=1)\times\{1-P(D_{e*}=1)\}}{\{1-P(D_e=1)\}\times P(D_{e*}=1)}\right\}\\ &= \log\left\{\frac{P(D=1|e=1)\times P(D=0|e*=0)}{P(D=0|e=1)\times P(D=1|e*=0)}\right\}\\ &= \log\left\{\frac{[\sum_m P(D=1|e=1,m)P(m|e=1)]\times[\sum_m P(D=0|e*=0,m)P(m|e*=0)]}{[\sum_m P(D=0|e=1,m)P(m|e=1)]\times[\sum_m P(D=1|e*=0,m)P(m|e*=0)]}\right\}\end{aligned}$$

The effect ($\beta_{ED|M}(m)$) of adjusting for mediator *M* by logistic regression model is given

$$\begin{aligned}\beta_{ED|M}(m) &= \text{logit}\{P(D=1|e=1,m)\} - \text{logit}\{P(D=1|e*=0,m)\}\\ &= \log\left\{\frac{P(D=1|e=1,m)\times P(D=0|e*=0,m)}{P(D=0|e=1,m)\times P(D=1|e*=0,m)}\right\}\\ &= \beta_0\end{aligned}$$

Therefore,

For peer review only - http://bmjopen.bmj.com/site/about/guidelines.xhtml

$$\text{bias} = \beta_0 - \log(OR_{E\to D}^{TE})$$
$$= \log\left\{\frac{\exp(\beta_0)}{\exp(\beta_0)\dfrac{\exp(\beta_2)\times A_1 + \exp(\beta_2)\times B_1 + C_1 + D_1}{\exp(\beta_2)\times A_1 + B_1 + \exp(\beta_2)\times C_1 + D_1}}\right\}$$
$$= \log\left\{\frac{\exp(\beta_2)\times A_1 + B_1 + \exp(\beta_2)\times C_1 + D_1}{\exp(\beta_2)\times A_1 + \exp(\beta_2)\times B_1 + C_1 + D_1}\right\}$$

where

$$A_1 = \exp(\beta_1 + \alpha_0)\times(1+\exp(\beta_0+\alpha_1))\times\exp(\alpha_0)\times(1+\exp(\alpha_1))$$
$$B_1 = \exp(\beta_1 + \alpha_0)\times(1+\exp(\beta_0+\alpha_1))\times(1+\exp(\beta_2+\alpha_1))$$
$$C_1 = (1+\exp(\beta_0+\beta_2+\alpha_1))\times\exp(\alpha_0)\times(1+\exp(\alpha_1))$$
$$D_1 = (1+\exp(\beta_0+\beta_2+\alpha_1))\times(1+\exp(\beta_2+\alpha_1))$$

Focusing on the difference of between $\exp(\beta_2)\times B_1 + C_1$ and $B_1 + \exp(\beta_2)\times C_1$.

$$T(\beta_1) = \exp(\beta_2)\times B_1 + C_1 - (B_1 + \exp(\beta_2)\times C_1)$$
$$= \exp(\beta_2)\times(B_1 - C_1) - (B_1 - C_1)$$
$$= (\exp(\beta_2)-1)\times(B_1 - C_1)$$
$$= (\exp(\beta_2)-1)\times(\exp(\beta_1+\alpha_0)\times(1+\exp(\beta_0+\alpha_1))\times(1+\exp(\beta_2+\alpha_1))$$
$$-(1+\exp(\beta_0+\beta_2+\alpha_1))\times\exp(\alpha_0)\times(1+\exp(\alpha_1)))$$
$$= (\exp(\beta_2)-1)\times\exp(\alpha_0)\times[\exp(\beta_1)\times(1+\exp(\beta_0+\alpha_1)\times(1+\exp(\beta_2+\alpha_1))$$
$$-(1+\exp(\beta_0+\beta_2+\alpha_1))\times(1+\exp(\alpha_1))]$$

Then, detailed dissection:

1: $\beta_2 = 0$, $bias = 0$.

2: $\beta_2 > 0$,

① $\beta_1 = 0$:(i) $\beta_0 = 0$, $bias = 0$;(ii) $\beta_0 > 0$, $bias > 0$;(iii) $\beta_0 < 0$, $bias < 0$.

② $\beta_1 < 0$:(i) $\beta_0 = 0$, $bias > 0$;(ii) $\beta_0 > 0$, $bias > 0$;(iii) $\beta_0 < 0$, $bias > 0$.

proof (iii)

$$T(\beta_1) = (\exp(\beta_2)-1)\times\exp(\alpha_0)$$
$$\times\{\exp(\beta_1)\times[1+\exp(\beta_0+\alpha_1)+\exp(\beta_2+\alpha_1)+\exp(\beta_0+\beta_2+2\alpha_1)]$$
$$-[1+\exp(\beta_0+\beta_2+\alpha_1)+\exp(\alpha_1)+\exp(\beta_0+\beta_2+2\alpha_1)]\}$$

when $\beta_0 < 0$ and $\beta_2 > 0 \Rightarrow \exp(\beta_0)-1<0 \quad \exp(\beta_2)-1>0$

According to $(a-1)(b-1) = ab - a - b + 1$, when $(a-1)(b-1) < 0 \Rightarrow ab+1 < a+b$

For peer review only - http://bmjopen.bmj.com/site/about/guidelines.xhtml

$$1+\exp(\beta_0+\beta_2+\alpha_1)+\exp(\alpha_1)+\exp(\beta_0+\beta_2+2\alpha_1)$$
$$<1+\exp(\beta_0+\alpha_1)+\exp(\beta_2+\alpha_1)+\exp(\beta_0+\beta_2+2\alpha_1)$$
$$\Rightarrow \exp(\beta_0+\beta_2)+1<\exp(\beta_0)+\exp(\beta_2)$$

when

$$\beta_1<\log\left\{\frac{\exp(\beta_0+\beta_2)+1}{\exp(\beta_0)+\exp(\beta_2)}\right\}<0$$

$$\beta_1<\log\left\{\frac{1+\exp(\beta_0+\beta_2+\alpha_1)+\exp(\alpha_1)+\exp(\beta_0+\beta_2+2\alpha_1)}{1+\exp(\beta_0+\alpha_1)+\exp(\beta_2+\alpha_1)+\exp(\beta_0+\beta_2+2\alpha_1)}\right\}<0$$

$$\Rightarrow \exp(\beta_1)<\frac{1+\exp(\beta_0+\beta_2+\alpha_1)+\exp(\alpha_1)+\exp(\beta_0+\beta_2+2\alpha_1)}{1+\exp(\beta_0+\alpha_1)+\exp(\beta_2+\alpha_1)+\exp(\beta_0+\beta_2+2\alpha_1)}<1$$

$$\begin{aligned}\Rightarrow T(\beta_1)=&(\exp(\beta_2)-1)\times\exp(\alpha_0)\\ &\times\{\exp(\beta_1)\times[1+\exp(\beta_0+\alpha_1)+\exp(\beta_2+\alpha_1)+\exp(\beta_0+\beta_2+2\alpha_1)]\\ &-[1+\exp(\beta_0+\beta_2+\alpha_1)+\exp(\alpha_1)+\exp(\beta_0+\beta_2+2\alpha_1)]\}\\ &<0\end{aligned}$$

Therefore, when $\beta_2>0$, $\beta_1<0$, $\beta_0<0$, then $bias>0$.

③ $\beta_1>0$:(i) $\beta_0=0$, $bias<0$;(ii) $\beta_0<0$, $bias<0$;(iii) $\beta_0>0$, $bias<0$.

proof (iii)

$$\begin{aligned}T(\beta_1)=&(\exp(\beta_2)-1)\times\exp(\alpha_0)\\ &\times\{\exp(\beta_1)\times[1+\exp(\beta_0+\alpha_1)+\exp(\beta_2+\alpha_1)+\exp(\beta_0+\beta_2+2\alpha_1)]\\ &-[1+\exp(\beta_0+\beta_2+\alpha_1)+\exp(\alpha_1)+\exp(\beta_0+\beta_2+2\alpha_1)]\}\end{aligned}$$

when $\beta_0>0$ and $\beta_2>0\Rightarrow \exp(\beta_0)-1>0 \quad \exp(\beta_2)-1>0$

According to $(a-1)(b-1)=ab-a-b+1$, when $ab>0\Rightarrow ab+1>a+b$

$$1+\exp(\beta_0+\beta_2+\alpha_1)+\exp(\alpha_1)+\exp(\beta_0+\beta_2+2\alpha_1)$$
$$>1+\exp(\beta_0+\alpha_1)+\exp(\beta_2+\alpha_1)+\exp(\beta_0+\beta_2+2\alpha_1)$$
$$\Rightarrow \exp(\beta_0+\beta_2)+1>\exp(\beta_0)+\exp(\beta_2)$$

when

$$\beta_1>\log\left\{\frac{\exp(\beta_0+\beta_2)+1}{\exp(\beta_0)+\exp(\beta_2)}\right\}>0$$

$$\beta_1>\log\left\{\frac{1+\exp(\beta_0+\beta_2+\alpha_1)+\exp(\alpha_1)+\exp(\beta_0+\beta_2+2\alpha_1)}{1+\exp(\beta_0+\alpha_1)+\exp(\beta_2+\alpha_1)+\exp(\beta_0+\beta_2+2\alpha_1)}\right\}>0$$

$$\Rightarrow \exp(\beta_1)>\frac{1+\exp(\beta_0+\beta_2+\alpha_1)+\exp(\alpha_1)+\exp(\beta_0+\beta_2+2\alpha_1)}{1+\exp(\beta_0+\alpha_1)+\exp(\beta_2+\alpha_1)+\exp(\beta_0+\beta_2+2\alpha_1)}>1$$

For peer review only - http://bmjopen.bmj.com/site/about/guidelines.xhtml

$$\Rightarrow T(\beta_1) = (\exp(\beta_2) - 1) \times \exp(\alpha_0) \times \{\exp(\beta_1) \times [1 + \exp(\beta_0 + \alpha_1) + \exp(\beta_2 + \alpha_1) + \exp(\beta_0 + \beta_2 + 2\alpha_1)] - [1 + \exp(\beta_0 + \beta_2 + \alpha_1) + \exp(\alpha_1) + \exp(\beta_0 + \beta_2 + 2\alpha_1)]\} > 0$$

Therefore, when $\beta_2 > 0$, $\beta_1 > 0$, $\beta_0 > 0$, then $bias < 0$.

3: $\beta_2 < 0$,

① $\beta_1 = 0$:(i) $\beta_0 = 0$, $bias = 0$;(ii) $\beta_0 > 0$, $bias > 0$;(iii) $\beta_0 < 0$, $bias < 0$.

② $\beta_1 < 0$:(i) $\beta_0 = 0$, $bias < 0$;(ii) $\beta_0 < 0$, $bias < 0$;(iii) $\beta_0 > 0$, $bias < 0$.

proof (iii)

$$T(\beta_1) = (\exp(\beta_2) - 1) \times \exp(\alpha_0) \times \{\exp(\beta_1) \times [1 + \exp(\beta_0 + \alpha_1) + \exp(\beta_2 + \alpha_1) + \exp(\beta_0 + \beta_2 + 2\alpha_1)] - [1 + \exp(\beta_0 + \beta_2 + \alpha_1) + \exp(\alpha_1) + \exp(\beta_0 + \beta_2 + 2\alpha_1)]\}$$

when $\beta_0 > 0$ and $\beta_2 < 0 \Rightarrow \exp(\beta_0) - 1 > 0 \quad \exp(\beta_2) - 1 < 0$

According to $(a-1)(b-1) = ab - a - b + 1$, when $ab < 0 \Rightarrow ab + 1 < a + b$

$$1 + \exp(\beta_0 + \beta_2 + \alpha_1) + \exp(\alpha_1) + \exp(\beta_0 + \beta_2 + 2\alpha_1) < 1 + \exp(\beta_0 + \alpha_1) + \exp(\beta_2 + \alpha_1) + \exp(\beta_0 + \beta_2 + 2\alpha_1) \Rightarrow \exp(\beta_0 + \beta_2) + 1 < \exp(\beta_0) + \exp(\beta_2)$$

when

$$\beta_1 < \log\left\{\frac{\exp(\beta_0 + \beta_2) + 1}{\exp(\beta_0) + \exp(\beta_2)}\right\} < 0$$

$$\beta_1 < \log\left\{\frac{1 + \exp(\beta_0 + \beta_2 + \alpha_1) + \exp(\alpha_1) + \exp(\beta_0 + \beta_2 + 2\alpha_1)}{1 + \exp(\beta_0 + \alpha_1) + \exp(\beta_2 + \alpha_1) + \exp(\beta_0 + \beta_2 + 2\alpha_1)}\right\} < 0$$

$$\Rightarrow \exp(\beta_1) < \frac{1 + \exp(\beta_0 + \beta_2 + \alpha_1) + \exp(\alpha_1) + \exp(\beta_0 + \beta_2 + 2\alpha_1)}{1 + \exp(\beta_0 + \alpha_1) + \exp(\beta_2 + \alpha_1) + \exp(\beta_0 + \beta_2 + 2\alpha_1)} < 1$$

$$\Rightarrow T(\beta_1) = (\exp(\beta_2) - 1) \times \exp(\alpha_0) \times \{\exp(\beta_1) \times [1 + \exp(\beta_0 + \alpha_1) + \exp(\beta_2 + \alpha_1) + \exp(\beta_0 + \beta_2 + 2\alpha_1)] - [1 + \exp(\beta_0 + \beta_2 + \alpha_1) + \exp(\alpha_1) + \exp(\beta_0 + \beta_2 + 2\alpha_1)]\} > 0$$

Therefore, when $\beta_2 < 0$, $\beta_1 < 0$, $\beta_0 > 0$, then $bias < 0$.

③ $\beta_1 > 0$:(i) $\beta_0 = 0$, $bias > 0$;(ii) $\beta_0 > 0$, $bias > 0$;(iii) $\beta_0 < 0$, $bias > 0$.

For peer review only - http://bmjopen.bmj.com/site/about/guidelines.xhtml

proof (iii)

$$T(\beta_1) = (\exp(\beta_2) - 1) \times \exp(\alpha_0)$$
$$\times \{\exp(\beta_1) \times [1 + \exp(\beta_0 + \alpha_1) + \exp(\beta_2 + \alpha_1) + \exp(\beta_0 + \beta_2 + 2\alpha_1)]$$
$$-[1 + \exp(\beta_0 + \beta_2 + \alpha_1) + \exp(\alpha_1) + \exp(\beta_0 + \beta_2 + 2\alpha_1)]\}$$

when $\beta_0 < 0$ and $\beta_2 < 0 \Rightarrow \exp(\beta_0) - 1 < 0 \quad \exp(\beta_2) - 1 < 0$

According to $(a-1)(b-1) = ab - a - b + 1$, when $ab > 0 \Rightarrow ab + 1 > a + b$

$$1 + \exp(\beta_0 + \beta_2 + \alpha_1) + \exp(\alpha_1) + \exp(\beta_0 + \beta_2 + 2\alpha_1)$$
$$> 1 + \exp(\beta_0 + \alpha_1) + \exp(\beta_2 + \alpha_1) + \exp(\beta_0 + \beta_2 + 2\alpha_1)$$
$$\Rightarrow \exp(\beta_0 + \beta_2) + 1 > \exp(\beta_0) + \exp(\beta_2)$$

when

$$\beta_1 > \log\left\{\frac{\exp(\beta_0 + \beta_2) + 1}{\exp(\beta_0) + \exp(\beta_2)}\right\} > 0$$

$$\beta_1 > \log\left\{\frac{1 + \exp(\beta_0 + \beta_2 + \alpha_1) + \exp(\alpha_1) + \exp(\beta_0 + \beta_2 + 2\alpha_1)}{1 + \exp(\beta_0 + \alpha_1) + \exp(\beta_2 + \alpha_1) + \exp(\beta_0 + \beta_2 + 2\alpha_1)}\right\} > 0$$

$$\Rightarrow \exp(\beta_1) > \frac{1 + \exp(\beta_0 + \beta_2 + \alpha_1) + \exp(\alpha_1) + \exp(\beta_0 + \beta_2 + 2\alpha_1)}{1 + \exp(\beta_0 + \alpha_1) + \exp(\beta_2 + \alpha_1) + \exp(\beta_0 + \beta_2 + 2\alpha_1)} > 1$$

$$\Rightarrow T(\beta_1) = (\exp(\beta_2) - 1) \times \exp(\alpha_0)$$
$$\times \{\exp(\beta_1) \times [1 + \exp(\beta_0 + \alpha_1) + \exp(\beta_2 + \alpha_1) + \exp(\beta_0 + \beta_2 + 2\alpha_1)]$$
$$-[1 + \exp(\beta_0 + \beta_2 + \alpha_1) + \exp(\alpha_1) + \exp(\beta_0 + \beta_2 + 2\alpha_1)]\}$$
$$< 0$$

Therefore, when $\beta_2 < 0$, $\beta_1 > 0$, $\beta_0 < 0$, then $bias > 0$.

In conclusion:

1: $\beta_2 = 0$, $bias = 0$.

2: $\beta_2 \neq 0$, $\beta_1 = 0$:(i) $\beta_0 = 0$, $bias = 0$;(ii) $\beta_0 > 0$, $bias > 0$;(iii) $\beta_0 < 0$, $bias < 0$.

3: (i) $\beta_1\beta_2 > 0$, $bias < 0$. (ii) $\beta_1\beta_2 < 0$, $bias > 0$.

For peer review only - http://bmjopen.bmj.com/site/about/guidelines.xhtml

Supplementary A

The theoretical results of others causal diagrams (Figure 1b-Figure 1f) have been shown in the supplementary of manuscript.

(1) Figure 1(b) is a depiction through two series mediators, decomposing total effects into direct ($E \to D$) and indirect ($E \to M_1 \to M_2 \to D$) components.

On the odds ratio ($OR^{TE}_{E\to D}$) scale, the total effect ($\beta^{TE}_{E\to D} = \log(OR^{TE}_{E\to D})$), comparing exposure level e with $e*$, we could obtain the total effect:

$$
\begin{aligned}
\beta^{TE}_{E\to D} &= \log(OR^{TE}_{E\to D}) \\
&= \log\left\{\frac{P(D_e=1)/\{1-P(D_e=1)\}}{P(D_{e*}=1)/\{1-P(D_{e*}=1)\}}\right\} \\
&= \log\left\{\frac{P(D_e=1)\times\{1-P(D_{e*}=1)\}}{\{1-P(D_e=1)\}\times P(D_{e*}=1)}\right\} \\
&= \log\left\{\frac{P(D=1\,|\,e=1)\times P(D=0\,|\,e*=0)}{P(D=0\,|\,e=1)\times P(D=1\,|\,e*=0)}\right\} \\
&= \log\left\{\frac{\xi_1}{\xi_2}\right\}
\end{aligned}
$$

$$\xi_1 = [\sum_{m_1 m_2} P(D=1\,|\,e=1,m_2)P(m_2\,|\,m_1)P(m_1\,|\,e=1)]\times[\sum_{m_1 m_2} P(D=0\,|\,e*=0,m_2)P(m_2\,|\,m_1)P(m_1\,|\,e*=0)]$$

$$\xi_2 = [\sum_{m_1 m_2} P(D=0\,|\,e=1,m_2)P(m_2\,|\,m_1)P(m_1\,|\,e=1)]\times[\sum_{m_1 m_2} P(D=1\,|\,e*=0,m_2)P(m_2\,|\,m_1)P(m_1\,|\,e*=0)]$$

The effect ($\beta_{ED|M_1}(m_1)$) of adjusting for mediator M_1 by logistic regression model can be given

$$
\begin{aligned}
\beta_{ED|M_1}(m_1) &= \text{logit}\{P(D=1\,|\,e=1,m_1)\} - \text{logit}\{P(D=1\,|\,e*=0,m_1)\} \\
&= \log\left\{\frac{P(D=1\,|\,e=1,m_1)P(D=0\,|\,e*=0,m_1)}{P(D=0\,|\,e=1,m_1)P(D=1\,|\,e*=0,m_1)}\right\} \\
&= \log\left\{\frac{[\sum_{m_2} P(D=1\,|\,e=1,m_2)P(m_2\,|\,m_1)]\times[\sum_{m_2} P(D=0\,|\,e*=0,m_2)P(m_2\,|\,m_1)]}{[\sum_{m_2} P(D=0\,|\,e=1,m_2)P(m_2\,|\,m_1)]\times[\sum_{m_2} P(D=1\,|\,e*=0,m_2)P(m_2\,|\,m_1)]}\right\}
\end{aligned}
$$

The effect ($\beta_{ED|M_2}(m_2)$) of adjusting for mediator M_2 by logistic regression model can be given

$$
\begin{aligned}
&\beta_{ED|M_2}(m_2) \\
&= \text{logit}\{P(D=1\,|\,e=1,m_2)\} - \text{logit}\{P(D=1\,|\,e*=0,m_2)\} \\
&= \log\left\{\frac{P(D=1\,|\,e=1,m_2)P(D=0\,|\,e*=0,m_2)}{P(D=0\,|\,e=1,m_2)P(D=1\,|\,e*=0,m_2)}\right\}
\end{aligned}
$$

For peer review only

The effect ($\beta_{ED|M_1,M_2}(m_1,m_2)$) of adjusting for mediator M_1 M_2 by logistic regression model can be given

$$\beta_{ED|M_1,M_2}(m_1,m_2) = \text{logit}\{P(D=1\,|\,e=1,m_1,m_2)\} - \text{logit}\{P(D=1\,|\,e^*=0,m_1,m_2)\}$$
$$= \log\left\{\frac{P(D=1\,|\,e=1,m_1,m_2)P(D=0\,|\,e^*=0,m_1,m_2)}{P(D=0\,|\,e=1,m_1,m_2)P(D=1\,|\,e^*=0,m_1,m_2)}\right\}$$
$$= \log\left\{\frac{P(D=1\,|\,e=1,m_2)P(D=0\,|\,e^*=0,m_2)}{P(D=0\,|\,e=1,m_2)P(D=1\,|\,e^*=0,m_2)}\right\}$$

Therefore, we could evaluate the biases that contains three adjustment models: A) adjustment for M_1, $bias(m_1) = \beta_{ED|M_1}(m_1) - \beta_{E\to D}^{TE}$; B) adjustment for M_2, $bias(m_2) = \beta_{ED|M_2}(m_2) - \beta_{E\to D}^{TE}$ and C) adjustment for M_1 and M_2, $bias(m_1,m_2) = \beta_{ED|M_1,M_2}(m_1,m_2) - \beta_{E\to D}^{TE}$.

(2) Figure 1c shows that the exposure E independently causes M_1 and M_2 and indirectly influences the outcome D through M_1 and M_2, forming three causal paths $E\to D$, $E\to M_1\to D$ and $E\to M_2\to D$.

On the odds ratio ($OR_{E\to D}^{TE}$) scale, the total effect ($\beta_{E\to D}^{TE} = \log(OR_{E\to D}^{TE})$), comparing exposure level e with e^*, we could obtain the total effect:

$$\beta_{E\to D}^{TE} = \log(OR_{E\to D}^{TE})$$
$$= \log\left\{\frac{P(D_e=1)/\{1-P(D_e=1)\}}{P(D_{e^*}=1)/\{1-P(D_{e^*}=1)\}}\right\}$$
$$= \log\left\{\frac{P(D_e=1)\times\{1-P(D_{e^*}=1)\}}{\{1-P(D_e=1)\}\times P(D_{e^*}=1)}\right\}$$
$$= \log\left\{\frac{P(D=1\,|\,e=1)\times P(D=0\,|\,e^*=0)}{P(D=0\,|\,e=1)\times P(D=1\,|\,e^*=0)}\right\}$$
$$= \log\left\{\frac{\xi_1}{\xi_2}\right\}$$

$$\xi_1 = [\sum_{m_1m_2} P(D=1\,|\,e=1,m_1,m_2)P(m_2\,|\,e=1)P(m_1\,|\,e=1)]\times[\sum_{m_1m_2} P(D=0\,|\,e^*=0,m_1,m_2)P(m_2\,|\,e^*=0)P(m_1\,|\,e^*=0)]$$
$$\xi_2 = [\sum_{m_1m_2} P(D=0\,|\,e=1,m_1,m_2)P(m_2\,|\,e=1)P(m_1\,|\,e=1)]\times[\sum_{m_1m_2} P(D=1\,|\,e^*=0,m_1,m_2)P(m_2\,|\,e^*=0)P(m_1\,|\,e^*=0)]$$

The effect ($\beta_{ED|M_1}(m_1)$) of adjusting for mediator M_1 by logistic regression model can be given

For peer review only

$$\beta_{ED|M_1}(m_1) = \text{logit}\{P(D=1|e=1,m_1)\} - \text{logit}\{P(D=1|e^*=0,m_1)\}$$
$$= \log\left\{\frac{P(D=1|e=1,m_1)P(D=0|e^*=0,m_1)}{P(D=0|e=1,m_1)P(D=1|e^*=0,m_1)}\right\}$$
$$= \log\left\{\frac{[\sum_{m_2} P(D=1|e=1,m_1,m_2)P(m_2|e=1)]\times[\sum_{m_2} P(D=0|e^*=0,m_1,m_2)P(m_2|e^*=0)]}{[\sum_{m_2} P(D=0|e=1,m_1,m_2)P(m_2|e=1)]\times[\sum_{m_2} P(D=1|e^*=0,m_1,m_2)P(m_2|e^*=0)]}\right\}$$

The effect ($\beta_{ED|M_2}(m_2)$) of adjusting for mediator M_2 by logistic regression model can be given

$$\beta_{ED|M_2}(m_2) = \text{logit}\{P(D=1|e=1,m_2)\} - \text{logit}\{P(D=1|e^*=0,m_2)\}$$
$$= \log\left\{\frac{P(D=1|e=1,m_2)P(D=0|e^*=0,m_2)}{P(D=0|e=1,m_2)P(D=1|e^*=0,m_2)}\right\}$$
$$= \log\left\{\frac{[\sum_{m_1} P(D=1|e=1,m_1,m_2)P(m_1|e=1)]\times[\sum_{m_1} P(D=0|e^*=0,m_1,m_2)P(m_1|e^*=0)]}{[\sum_{m_1} P(D=0|e=1,m_1,m_2)P(m_1|e=1)]\times[\sum_{m_1} P(D=1|e^*=0,m_1,m_2)P(m_1|e^*=0)]}\right\}$$

The effect ($\beta_{ED|M_1,M_2}(m_1,m_2)$) of adjusting for mediator M_1 M_2 by logistic regression model can be given

$$\beta_{ED|M_1,M_2}(m_1,m_2) = \text{logit}\{P(D=1|e=1,m_1,m_2)\} - \text{logit}\{P(D=1|e^*=0,m_1,m_2)\}$$
$$= \log\left\{\frac{P(D=1|e=1,m_1,m_2)P(D=0|e^*=0,m_1,m_2)}{P(D=0|e=1,m_1,m_2)P(D=1|e^*=0,m_1,m_2)}\right\}$$

Therefore, we could evaluate the biases that contains three adjustment models: A) adjustment for M_1, $bias(m_1) = \beta_{ED|M_1}(m_1) - \beta^{TE}_{E\to D}$; B) adjustment for M_2, $bias(m_2) = \beta_{ED|M_2}(m_2) - \beta^{TE}_{E\to D}$ and C) adjustment for M_1 and M_2, $bias(m_1,m_2) = \beta_{ED|M_1,M_2}(m_1,m_2) - \beta^{TE}_{E\to D}$.

(3) In Figure 1d, there exists five paths from E to D: $E\to D$, $E\to M_1\to D$, $E\to M_2\to D$, $E\to M_1\leftarrow M_2\to D$ and $E\to M_2\to M_1\to D$. In particular, the path $E\to M_1\leftarrow M_2\to D$ is a blocked path, due to the M_1 being a collider node.

On the odds ratio ($OR^{TE}_{E\to D}$) scale, the total effect ($\beta^{TE}_{E\to D} = \log(OR^{TE}_{E\to D})$), comparing exposure level e with e^*, we could obtain the total effect:

For peer review only

$$\beta_{E\to D}^{TE} = \log(OR_{E\to D}^{TE})$$
$$= \log\left\{\frac{P(D_e=1)/\{1-P(D_e=1)\}}{P(D_{e*}=1)/\{1-P(D_{e*}=1)\}}\right\}$$
$$= \log\left\{\frac{P(D_e=1)\times\{1-P(D_{e*}=1)\}}{\{1-P(D_e=1)\}\times P(D_{e*}=1)}\right\}$$
$$= \log\left\{\frac{P(D=1|e=1)\times P(D=0|e*=0)}{P(D=0|e=1)\times P(D=1|e*=0)}\right\}$$
$$= \log\left\{\frac{\xi_1}{\xi_2}\right\}$$

$$\xi_1 = [\sum_{m_1 m_2} P(D=1|e=1,m_1,m_2)P(m_2|e=1)P(m_1|e=1,m_2)]$$
$$\times[\sum_{m_1 m_2} P(D=0|e*=0,m_1,m_2)P(m_2|e*=0)P(m_1|e*=0,m_2)]$$
$$\xi_2 = [\sum_{m_1 m_2} P(D=0|e=1,m_1,m_2)P(m_2|e=1)P(m_1|e=1,m_2)]$$
$$\times[\sum_{m_1 m_2} P(D=1|e*=0,m_1,m_2)P(m_2|e*=0)P(m_1|e*=0,m_2)]$$

The effect ($\beta_{ED|M_1}(m_1)$) of adjusting for mediator M_1 by logistic regression model can be given

$$\beta_{ED|M_1}(m_1) = \text{logit}\{P(D=1|e=1,m_1)\} - \text{logit}\{P(D=1|e*=0,m_1)\}$$
$$= \log\left\{\frac{P(D=1|e=1,m_1)P(D=0|e*=0,m_1)}{P(D=0|e=1,m_1)P(D=1|e*=0,m_1)}\right\}$$
$$= \log\left\{\frac{[\sum_{m_2} P(D=1|e=1,m_1,m_2)P(m_2|e=1,m_1)]\times[\sum_{m_2} P(D=0|e*=0,m_1,m_2)P(m_2|e*=0,m_1)]}{[\sum_{m_2} P(D=0|e=1,m_1,m_2)P(m_2|e=1,m_1)]\times[\sum_{m_2} P(D=1|e*=0,m_1,m_2)P(m_2|e*=0,m_1)]}\right\}$$
$$= \log\left\{\frac{\xi_1}{\xi_2}\right\}$$

$$\xi_1 = [\sum_{m_2} P(D=1|e=1,m_1,m_2)\frac{P(m_1|e=1,m_2)P(m_2|e=1)}{\sum_{m_2} P(m_1|e=1,m_2)P(m_2|e=1)}]$$
$$\times[\sum_{m_2} P(D=0|e*=0,m_1,m_2)\frac{P(m_1|e*=0,m_2)P(m_2|e*=0)}{\sum_{m_2} P(m_1|e*=0,m_2)P(m_2|e*=0)}]$$
$$\xi_2 = [\sum_{m_2} P(D=0|e=1,m_1,m_2)\frac{P(m_1|e=1,m_2)P(m_2|e=1)}{\sum_{m_2} P(m_1|e=1,m_2)P(m_2|e=1)}]$$
$$\times[\sum_{m_2} P(D=1|e*=0,m_1,m_2)\frac{P(m_1|e*=0,m_2)P(m_2|e*=0)}{\sum_{m_2} P(m_1|e*=0,m_2)P(m_2|e*=0)}]$$

The effect ($\beta_{ED|M_2}(m_2)$) of adjusting for mediator M_2 by logistic regression model can be given

$$\beta_{ED|M_2}(m_2) = \text{logit}\{P(D=1|e=1,m_2)\} - \text{logit}\{P(D=1|e^*=0,m_2)\}$$
$$= \log\left\{\frac{P(D=1|e=1,m_2)P(D=0|e^*=0,m_2)}{P(D=0|e=1,m_2)P(D=1|e^*=0,m_2)}\right\}$$
$$= \log\left\{\frac{[\sum_{m_1} P(D=1|e=1,m_1,m_2)P(m_1|e=1,m_2)]\times[\sum_{m_1} P(D=0|e^*=0,m_1,m_2)P(m_1|e^*=0,m_2)]}{[\sum_{m_1} P(D=0|e=1,m_1,m_2)P(m_1|e=1,m_2)]\times[\sum_{m_1} P(D=1|e^*=0,m_1,m_2)P(m_1|e^*=0,m_2)]}\right\}$$

The effect ($\beta_{ED|M_1,M_2}(m_1,m_2)$) of adjusting for mediator M_1 M_2 by logistic regression model can be given

$$\beta_{ED|M_1,M_2}(m_1,m_2) = \text{logit}\{P(D=1|e=1,m_1,m_2)\} - \text{logit}\{P(D=1|e^*=0,m_1,m_2)\}$$
$$= \log\left\{\frac{P(D=1|e=1,m_1,m_2)P(D=0|e^*=0,m_1,m_2)}{P(D=0|e=1,m_1,m_2)P(D=1|e^*=0,m_1,m_2)}\right\}$$

Therefore, we could evaluate the biases that contains three adjustment models: A) adjustment for M_1, $bias(m_1) = \beta_{ED|M_1}(m_1) - \beta_{E\to D}^{TE}$; B) adjustment for M_2, $bias(m_2) = \beta_{ED|M_2}(m_2) - \beta_{E\to D}^{TE}$ and C) adjustment for M_1 and M_2, $bias(m_1,m_2) = \beta_{ED|M_1,M_2}(m_1,m_2) - \beta_{E\to D}^{TE}$.

(4) In Figure 1e, the causal diagrams contained a confounder of exposure-outcome relationship. On the odds ratio ($OR_{E\to D}^{TE}$) scale, the total effect ($\beta_{E\to D}^{TE} = \log(OR_{E\to D}^{TE})$), comparing exposure level *e* with *e**, we could obtain the total effect:

$$\beta_{E\to D}^{TE} = \log(OR_{E\to D}^{TE})$$
$$= \log\left\{\frac{P(D_e=1)/\{1-P(D_e=1)\}}{P(D_{e^*}=1)/\{1-P(D_{e^*}=1)\}}\right\}$$
$$= \log\left\{\frac{P(D_e=1)\times\{1-P(D_{e^*}=1)\}}{\{1-P(D_e=1)\}\times P(D_{e^*}=1)}\right\}$$
$$= \log\left\{\frac{P(D=1|e=1)\times P(D=0|e^*=0)}{P(D=0|e=1)\times P(D=1|e^*=0)}\right\}$$
$$= \log\left\{\frac{[\sum_{mu} P(D=1|e=1,m,u)P(m|e=1)P(u)]\times[\sum_{mu} P(D=0|e^*=0,m,u)P(m|e^*=0)P(u)]}{[\sum_{mu} P(D=0|e=1,m,u)P(m|e=1)P(u)]\times[\sum_{mu} P(D=1|e^*=0,m,u)P(m|e^*=0)P(u)]}\right\}$$

The effect ($\beta_{ED|M}(m)$) of adjusting for mediator M by logistic regression model can be given

For peer review only

$$\beta_{ED|M}(m) = \log it(P(D=1|e=1,m)) - \log it(P(D=1|e^*=0,m))$$
$$= \log\left\{\frac{P(D=1|e=1,m)\times P(D=0|e^*=0,m)}{P(D=0|e=1,m)\times P(D=1|e^*=0,m)}\right\}$$
$$= \log\left\{\frac{[\sum_u P(D=1|e=1,m,u)p(u|e=1,m)]\times[\sum_u P(D=0|e^*=0,m,u)p(u|e^*=0,m)]}{[\sum_u P(D=0|e=1,m,u)p(u|e=1,m)]\times[\sum_u P(D=1|e^*=0,m,u)p(u|e^*=0,m)]}\right\}$$
$$= \log\left\{\frac{[\sum_u P(D=1|e=1,m,u)\frac{p(e=1|u)p(u)}{\sum_u p(e=1|u)p(u)}]\times[\sum_u P(D=0|e^*=0,m,u)\frac{p(e^*=0|u)p(u)}{\sum_u p(e^*=0|u)p(u)}]}{[\sum_u P(D=0|e=1,m,u)\frac{p(e=1|u)p(u)}{\sum_u p(e=1|u)p(u)}]\times[\sum_u P(D=1|e^*=0,m,u)\frac{p(e^*=0|u)p(u)}{\sum_u p(e^*=0|u)p(u)}]}\right\}$$

Therefore, we could evaluate the biases of adjustment models: $bias(m) = \beta_{ED|M}(m) - \beta^{TE}_{E\to D}$.

(5) Figure 1f is a depiction of two parallel mediators M_1 and M_2 with confounder.

On the odds ratio ($OR^{TE}_{E\to D}$) scale, the total effect ($\beta^{TE}_{E\to D} = \log(OR^{TE}_{E\to D})$), comparing exposure level *e* with *e**, we could obtain the total effect:

$$\beta^{TE}_{E\to D} = \log(OR^{TE}_{E\to D})$$
$$= \log\left\{\frac{P(D_e=1)/\{1-P(D_e=1)\}}{P(D_{e^*}=1)/\{1-P(D_{e^*}=1)\}}\right\}$$
$$= \log\left\{\frac{P(D_e=1)\times\{1-P(D_{e^*}=1)\}}{\{1-P(D_e=1)\}\times P(D_{e^*}=1)}\right\}$$
$$= \log\left\{\frac{P(D=1|e=1)\times P(D=0|e^*=0)}{P(D=0|e=1)\times P(D=1|e^*=0)}\right\}$$
$$= \log\left\{\frac{\xi_1}{\xi_2}\right\}$$

$$\xi_1 = [\sum_{m_1m_2u} P(D=1|e=1,m_1,m_2,u)P(m_2|e=1)P(m_1|e=1)P(u)]$$
$$\times[\sum_{m_1m_2u} P(D=0|e^*=0,m_1,m_2,u)P(m_2|e^*=0)P(m_1|e^*=0)P(u)]$$
$$\xi_2 = [\sum_{m_1m_2u} P(D=0|e=1,m_1,m_2,u)P(m_2|e=1)P(m_1|e=1)P(u)]$$
$$\times[\sum_{m_1m_2u} P(D=1|e^*=0,m_1,m_2,u)P(m_2|e^*=0)P(m_1|e^*=0)P(u)]$$

The effect ($\beta_{ED|M_1}(m_1)$) of adjusting for mediator M_1 by logistic regression model can be given

For peer review only

$$\beta_{ED|M_1}(m_1) = \text{logit}\{P(D=1|e=1,m_1)\} - \text{logit}\{P(D=1|e^*=0,m_1)\}$$
$$= \log\left\{\frac{P(D=1|e=1,m_1)P(D=0|e^*=0,m_1)}{P(D=0|e=1,m_1)P(D=1|e^*=0,m_1)}\right\}$$
$$= \log\left\{\frac{\xi_1}{\xi_2}\right\}$$

$$\xi_1 = [\sum_{m_2 u} P(D=1|e=1,m_1,m_2,u)\frac{P(m_2|e=1)P(e=1|u)P(u)}{\sum_u P(e=1|u)P(u)}]$$
$$\times[\sum_{m_2 u} P(D=0|e^*=0,m_1,m_2,u)\frac{P(m_2|e^*=0)P(e^*=0|u)P(u)}{\sum_u P(e^*=0|u)P(u)}]$$
$$\xi_2 = [\sum_{m_2 u} P(D=0|e=1,m_1,m_2,u)\frac{P(m_2|e=1)P(e=1|u)P(u)}{\sum_u P(e=1|u)P(u)}]$$
$$\times[\sum_{m_2 u} P(D=1|e^*=0,m_1,m_2,u)\frac{P(m_2|e^*=0)P(e^*=0|u)P(u)}{\sum_u P(e^*=0|u)P(u)}]$$

The effect ($\beta_{ED|M_2}(m_2)$) of adjusting for mediator M_2 by logistic regression model can be given

$$\beta_{ED|M_2}(m_2) = \text{logit}\{P(D=1|e=1,m_2)\} - \text{logit}\{P(D=1|e^*=0,m_2)\}$$
$$= \log\left\{\frac{P(D=1|e=1,m_2)P(D=0|e^*=0,m_2)}{P(D=0|e=1,m_2)P(D=1|e^*=0,m_2)}\right\}$$
$$= \log\left\{\frac{\xi_1}{\xi_2}\right\}$$

$$\xi_1 = [\sum_{m_1 u} P(D=1|e=1,m_1,m_2,u)\frac{P(m_1|e=1)P(e=1|u)P(u)}{\sum_u P(e=1|u)P(u)}]$$
$$\times[\sum_{m_1 u} P(D=0|e^*=0,m_1,m_2,u)\frac{P(m_1|e^*=0)P(e^*=0|u)P(u)}{\sum_u P(e^*=0|u)P(u)}]$$
$$\xi_2 = [\sum_{m_1 u} P(D=0|e=1,m_1,m_2,u)\frac{P(m_1|e=1)P(e=1|u)P(u)}{\sum_u P(e=1|u)P(u)}]$$
$$\times[\sum_{m_1 u} P(D=1|e^*=0,m_1,m_2,u)\frac{P(m_1|e^*=0)P(e^*=0|u)P(u)}{\sum_u P(e^*=0|u)P(u)}]$$

The effect ($\beta_{ED|M_1,M_2}(m_1,m_2)$) of adjusting for mediator M_1 M_2 by logistic regression model can be given

$$\begin{aligned}
&\beta_{ED|M_1,M_2}(m_1,m_2)\\
&=\mathrm{logit}\{P(D=1|e=1,m_1,m_2)\}-\mathrm{logit}\{P(D=1|e^*=0,m_1,m_2)\}\\
&=\log\left\{\frac{P(D=1|e=1,m_1,m_2)P(D=0|e^*=0,m_1,m_2)}{P(D=0|e=1,m_1,m_2)P(D=1|e^*=0,m_1,m_2)}\right\}\\
&=\log\left\{\frac{[\sum_u P(D=1|e=1,m_1,m_2,u)\frac{P(e=1|u)P(u)}{\sum_u P(e=1|u)P(u)}]\times[\sum_u P(D=0|e^*=0,m_1,m_2,u)\frac{P(e=1|u)P(u)}{\sum_u P(e=1|u)P(u)}]}{[\sum_u P(D=0|e=1,m_1,m_2,u)\frac{P(e=1|u)P(u)}{\sum_u P(e=1|u)P(u)}]\times[\sum_u P(D=1|e^*=0,m_1,m_2,u)\frac{P(e=1|u)P(u)}{\sum_u P(e=1|u)P(u)}]}\right\}
\end{aligned}$$

Therefore, we could evaluate the biases that contains three adjustment models: A) adjustment for M_1, $bias(m_1)=\beta_{ED|M_1}(m_1)-\beta^{TE}_{E\to D}$; B) adjustment for M_2, $bias(m_2)=\beta_{ED|M_2}(m_2)-\beta^{TE}_{E\to D}$ and C) adjustment for M_1 and M_2, $bias(m_1,m_2)=\beta_{ED|M_1,M_2}(m_1,m_2)-\beta^{TE}_{E\to D}$.

Supplementary B

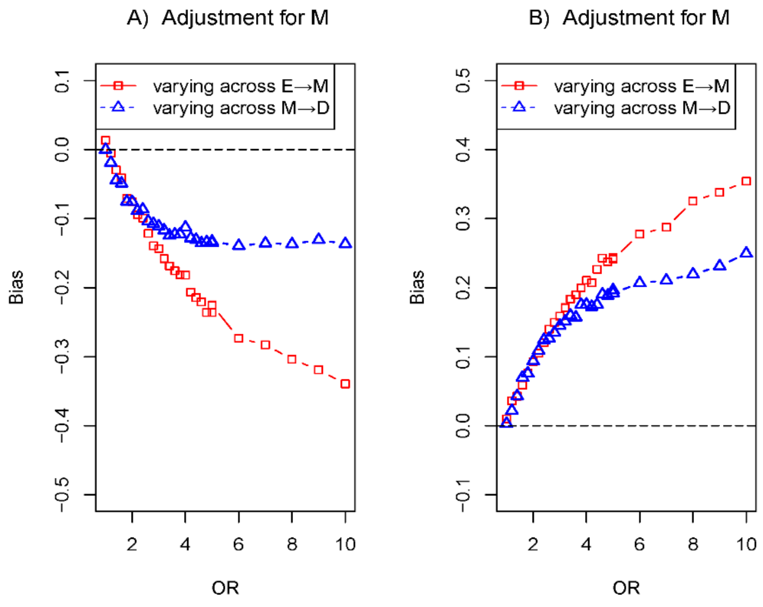

Figure S1: The biases with the effects $E\to M$ (red) and $M\to D$ (blue) increasing, respectively. Comparison of the bias of different effects in adjustment mediator.

The Figure S1-A obtained the result $bias<0$ in Figure 3a with the effects $E\to M$, $M\to D$ and $E\to D$ fixing to ln2. The Figure S1-B gained the result $bias>0$ in Figure 3c with the effects $E\to M$ and $E\to D$ fixing to ln2, effect $M\to D$ fixing to $-\ln 2$. We could obtain the bias performances of varying across the effects of exposure-mediator and mediator-outcome. The effect $E\to M$ of varying across was more sensitive than the effect $M\to D$ of varying across in Figure S1.

STROBE 2007 (v4) checklist of items to be included in reports of observational studies in epidemiology*
Checklist for cohort, case-control, and cross-sectional studies (combined)

Section/Topic	Item #	Recommendation	Reported on page #
**Title and abstract**	1	(*a*) Indicate the study's design with a commonly used term in the title or the abstract	1
		(*b*) Provide in the abstract an informative and balanced summary of what was done and what was found	2
**Introduction**			
Background/rationale	2	Explain the scientific background and rationale for the investigation being reported	3
Objectives	3	State specific objectives, including any pre-specified hypotheses	3-4
**Methods**			
Study design	4	Present key elements of study design early in the paper	4
Setting	5	Describe the setting, locations, and relevant dates, including periods of recruitment, exposure, follow-up, and data collection	5-6
Participants	6	(*a*) *Cohort study*—Give the eligibility criteria, and the sources and methods of selection of participants. Describe methods of follow-up<br>*Case-control study*—Give the eligibility criteria, and the sources and methods of case ascertainment and control selection. Give the rationale for the choice of cases and controls<br>*Cross-sectional study*—Give the eligibility criteria, and the sources and methods of selection of participants	5-6
		(*b*) *Cohort study*—For matched studies, give matching criteria and number of exposed and unexposed<br>*Case-control study*—For matched studies, give matching criteria and the number of controls per case	
Variables	7	Clearly define all outcomes, exposures, predictors, potential confounders, and effect modifiers. Give diagnostic criteria, if applicable	5-6
Data sources/ measurement	8*	For each variable of interest, give sources of data and details of methods of assessment (measurement). Describe comparability of assessment methods if there is more than one group	*5-6*
Bias	9	Describe any efforts to address potential sources of bias	5-6
Study size	10	Explain how the study size was arrived at	5-6
Quantitative variables	11	Explain how quantitative variables were handled in the analyses. If applicable, describe which groupings were chosen and why	Not applicable
Statistical methods	12	(*a*) Describe all statistical methods, including those used to control for confounding	4-6
		(*b*) Describe any methods used to examine subgroups and interactions	Not applicable
		(*c*) Explain how missing data were addressed	Not applicable
		(*d*) *Cohort study*—If applicable, explain how loss to follow-up was addressed<br>*Case-control study*—If applicable, explain how matching of cases and controls was addressed	Not applicable

For peer review only

		Cross-sectional study—If applicable, describe analytical methods taking account of sampling strategy	
		(*e*) Describe any sensitivity analyses	6
**Results**			
Participants	13*	(a) Report numbers of individuals at each stage of study—eg numbers potentially eligible, examined for eligibility, confirmed eligible, included in the study, completing follow-up, and analysed	Not applicable
		(b) Give reasons for non-participation at each stage	Not applicable
		(c) Consider use of a flow diagram	Not applicable
Descriptive data	14*	(a) Give characteristics of study participants (eg demographic, clinical, social) and information on exposures and potential confounders	7-10
		(b) Indicate number of participants with missing data for each variable of interest	Not applicable
		(c) *Cohort study*—Summarise follow-up time (eg, average and total amount)	Not applicable
Outcome data	15*	*Cohort study*—Report numbers of outcome events or summary measures over time	Not applicable
		Case-control study—Report numbers in each exposure category, or summary measures of exposure	Not applicable
		Cross-sectional study—Report numbers of outcome events or summary measures	7-10
Main results	16	(*a*) Give unadjusted estimates and, if applicable, confounder-adjusted estimates and their precision (eg, 95% confidence interval). Make clear which confounders were adjusted for and why they were included	7-10
		(*b*) Report category boundaries when continuous variables were categorized	Not applicable
		(*c*) If relevant, consider translating estimates of relative risk into absolute risk for a meaningful time period	Not applicable
Other analyses	17	Report other analyses done—eg analyses of subgroups and interactions, and sensitivity analyses	7-10
**Discussion**			
Key results	18	Summarise key results with reference to study objectives	11-12
Limitations	19	Discuss limitations of the study, taking into account sources of potential bias or imprecision. Discuss both direction and magnitude of any potential bias	11-12
Interpretation	20	Give a cautious overall interpretation of results considering objectives, limitations, multiplicity of analyses, results from similar studies, and other relevant evidence	11-12
Generalisability	21	Discuss the generalisability (external validity) of the study results	12
**Other information**			
Funding	22	Give the source of funding and the role of the funders for the present study and, if applicable, for the original study on which the present article is based	13

*Give information separately for cases and controls in case-control studies and, if applicable, for exposed and unexposed groups in cohort and cross-sectional studies.
Note: An Explanation and Elaboration article discusses each checklist item and gives methodological background and published examples of transparent reporting. The STROBE checklist is best used in conjunction with this article (freely available on the Web sites of PLoS Medicine at http://www.plosmedicine.org/, Annals of Internal Medicine at http://www.annals.org/, and Epidemiology at http://www.epidem.com/). Information on the STROBE Initiative is available at www.strobe-statement.org.

For peer review only

BMJ Open

Sensitivity analysis for mistakenly adjusting for mediators in estimating total effect: a simulation study

Journal:	*BMJ Open*
Manuscript ID	bmjopen-2016-015640.R2
Article Type:	Research
Date Submitted by the Author:	09-Jun-2017
Complete List of Authors:	Wang, Tingting; Shandong University, Department of Epidemiology and Biostatistics<br>Li, Hongkai; Shandong University, Department of Epidemiology and Biostatistics<br>Su, Ping; Shandong University, Department of Epidemiology and Biostatistics<br>Yu, Yuanyuan; Shandong University, Department of Epidemiology and Biostatistics<br>Sun, Xiaoru; Shandong University, Department of Epidemiology and Biostatistics<br>Liu, Yi; Shandong University, Department of Epidemiology and Biostatistics<br>Yuan, Zhongshang; Shandong University, Department of Epidemiology and Biostatistics<br>Xue, Fuzhong; Shandong University, Department of Epidemiology and Biostatistics
**Primary Subject Heading**:	Epidemiology
Secondary Subject Heading:	Public health
Keywords:	EPIDEMIOLOGY, PUBLIC HEALTH, STATISTICS & RESEARCH METHODS

SCHOLARONE™ Manuscripts

Sensitivity analysis for mistakenly adjusting for mediators in estimating total effect: a simulation study

Tingting Wang†, Hongkai Li†, Ping Su, Yuanyuan Yu, Xiaoru Sun, Yi Liu, Zhongshang Yuan, Fuzhong Xue*

Department of Biostatistics, School of Public Health, Shandong University, Jinan City, Shandong Province, P.R.China

Email addresses:

TTW: wangtingting161780@163.com

HKL: lihongkaiyouxiang@163.com

PS: sdsuping@163.com

YYY: yu_yy_1993@163.com

XRS: sunny150139@126.com

YL: liuyi238@sdu.edu.cn

ZSY: yuanzhongshang@sdu.edu.cn

*Corresponding Author

xuefzh@sdu.edu.cn (FZX)

Word Count: 3647

Abstract

Objectives: In observational studies, epidemiologists often attempt to estimate the total effect of exposure on outcome of interest. However, when the underlying diagram is unknown and only limited knowledge is available, dissecting biases performances are essential to estimate the total effect of exposure on outcome in mistakenly adjusting for mediators under logistic regression. Through simulation, we focus on six causal diagrams concerning different roles of mediators. Sensitivity analysis was conducted to assess the bias performances of varying across the effects of exposure-mediator and mediator-outcome in adjusting for mediator.

Setting: Based on the causal relationships in real world, we compare the biases of varying across the effects of exposure-mediator with the ones of varying across the effects of mediator-outcome under the situation of adjusting for mediator. The

For peer review only - http://bmjopen.bmj.com/site/about/guidelines.xhtml

magnitude of the bias was defined by the difference between the estimated effect using logistic regression and the total effect of the exposure on the outcome.

Results: In the following four scenarios: a single mediator, two series mediators, two independent parallel mediators or two correlated parallel mediators, the biases of varying across the effects of exposure-mediator was greater than the ones of varying across the effects mediator-outcome in adjusting for the mediator. While in other two scenarios: a single mediator or two independent parallel mediators in the presence of unobserved confounders, the biases of varying across the effects of exposure-mediator was less than the ones of varying across the effects mediator-outcome in adjusting for the mediator.

Conclusions: The biases were higher sensitive to the variation of effects of exposure-mediator than effects of mediator-outcome in adjusting for mediator in the absence of unobserved confounders; while the biases were higher sensitive to the variation of effects of mediator-outcome than effects of exposure-mediator in the presence of unobserved confounder.

Strengths and limitations of this study

1) For six different causal diagrams, we compared biases of distinct adjustment strategies with and without adjusting for mediators by conducting simulation studies.

2) Sensitivity analysis was conducted to assess the performances of varying across the effects of exposure-mediator and mediator-outcome.

3) The simulation schemes and parameters were conducted mainly based on real observational studies.

4) Combination of theoretical derivation and simulation studies make the results more credible.

For peer review only - http://bmjopen.bmj.com/site/about/guidelines.xhtml

5) The limitation of simulation studies was under the framework of logistic regression and only focused on binary variables.

Introduction

Estimating the total effect of the exposure (*E*) on the outcome (*D*) is a great challenge in epidemiology studies, because confounders are commonly confused with mediators.[1-3] If confounders and mediators are misclassified, the ability to control confounder in the estimation of the total effect of the exposure on the outcome is hampered. Actually, various strategies are used to eliminate confounding bias in non-randomized controlled studies. The conventional approaches contain multivariate regression, stratification, standardization and inverse-probability weighting, etc.[4-5] Furthermore, causal diagrams provides a formal conceptual framework to identify and select confounders,[6-7] so that it can avoid falling into analytic pitfalls.[8] In practice, even the underlying causal diagrams and the role of covariates (mediator, confounder, collider and instrumental variable) are not all learned, investigators usually adjusted for the covariates that are associated with the outcome and exposure.[9-12] Therefore, our paper focuses on the biases of varying across the effects of exposure-mediator ($E \rightarrow M$) and mediator-outcome ($M \rightarrow D$) in mistakenly adjusting for mediators under logistic regression model.

Several causal inference literatures have made a considerable contribution to mediation analysis by providing definitions for direct and indirect effects that allow for the effect decomposition of a total effect into a direct and an indirect effect.[13-21] Arbitrarily adjusting for a mediator would generally bias the estimate of the total effect of the exposure on the outcome.[8,22-23] Practically, it can mistakenly identify a non-confounding risk factor as a confounder. In the perspective of causal diagrams,

For peer review only - http://bmjopen.bmj.com/site/about/guidelines.xhtml

little attention was paid to the biases in adjusting for mediators under the logistic regression model in estimating the total effect of E on D. Hence, we focused on the sensitivity analysis technique to assess the biases of varying across the effects of $E \rightarrow M$ and $M \rightarrow D$ in adjusting for mediator.

In this paper, six typical causal diagrams corresponding to causal correlation are given in Figure 1: a single mediator (Figure 1a); two series mediators (Figure 1b); two independent parallel mediators (Figure 1c); two correlated parallel mediators (Figure 1d); a single mediator with an unobserved confounder (Figure 1e); two parallel mediators with an unobserved confounder (Figure 1f). The paper aims to explore the sensitivity of biases to the variation of the effects of $E \rightarrow D$ and $M \rightarrow D$ in adjusting for mediator. Hence, both theoretical proofs and quantitative simulations were performed to dissect the bias of varying across the effect of $E \rightarrow M$ and the one of varying across the effect of $M \rightarrow D$ in adjusting for mediators under logistic model.

Methods

A directed acyclic graph (*DAG*) is composed of variables (nodes) and arrows (directed edges) between nodes such that the graph is acyclic. The causal diagrams formalized as directed acyclic graphs (*DAG*s), providing investigators with powerful tools for bias assessment.[24] It provides a device for deducing the statistical associations implied by causal relations. Furthermore, given a set of observed statistical associations, a researcher armed with causal diagrams theory can systematically characterize all causal structures compatible with the observations.[25-26]

The total effect of the exposure on the outcome can be calculated based on the *do-calculus* and *back-door* criterion proposed by Judea Pearl.[27-28] For exposure X and

For peer review only - http://bmjopen.bmj.com/site/about/guidelines.xhtml

outcome Y, a set of variables Z satisfies the backdoor path criterion with respect to (X, Y) if no variable in Z is a descendant of X and Z blocks all back-door paths from X to Y. Then the effect of X on Y is given by the formula,

$$P(y \mid do(x)) = \sum_{Z} P(y \mid x, z) P(z)$$

Note that the expression on the right hand side of the equation is simply a standardized mean. The difference $E(Y \mid do(x')) - E(Y \mid do(x''))$ is taken as the definition of "causal effect", where x' and x'' are two distinct realizations of X.[23] The interventional distribution, such as that corresponding to $Y(x)$, namely $P(y|do(x))$, is not necessarily equal to a conditional distribution $P(y \mid x)$. It stands for the probability of $Y = y$ when the exposure X set to level x. The ignorability assumption $Y(x) \perp X$ states that if we happen to have information on the exposure variable, it does not give us any information about the outcome Y after the intervention $do(x)$ was performed. Besides it can be shown that if ignorability holds for $Y(x)$ and X (alternatively if there are no back-door paths from X to Y in the corresponding causal DAGs), then $P(y \mid do(x)) = P(y \mid x)$.[29-30]

Let D_e and M_e denote respectively the values of the outcome and mediator that would have been observed had the exposure E been set to level e. On the odds ratio ($OR^{TE}_{E \to D}$) scale, the total effect ($\beta^{TE}_{E \to D} = \log(OR^{TE}_{E \to D})$), comparing exposure level e with e*, is given by $OR^{TE}_{E \to D} = \dfrac{P(D_e = 1) / \{1 - P(D_e = 1)\}}{P(D_{e*} = 1) / \{1 - P(D_{e*} = 1)\}}$.[20-21] While the effect ($\beta_{ED|M}(m)$) of adjusting for mediator M by logistic regression model can be given

$$\begin{aligned} \beta_{ED|M}(m) &= \text{logit}\{P(D=1 \mid e=1, m)\} - \text{logit}\{P(D=1 \mid e^*=0, m)\} \\ &= \log\left\{\frac{P(D=1 \mid e=1, m) P(D=0 \mid e^*=0, m)}{P(D=0 \mid e=1, m) P(D=1 \mid e^*=0, m)}\right\} \end{aligned}$$

For peer review only - http://bmjopen.bmj.com/site/about/guidelines.xhtml

where $P(D=1|e,m)$ denotes the probability of $D=1$ when the exposure E, and mediator M, have been set to level e, and m, respectively. Taking Figure 1a as an example, the logistic regression is

$$\text{logit}\{P(D=1|e,m)\}=\alpha_1+\beta_0 e+\beta_2 m\,.$$

Therefore, the total effect ($\beta^{TE}_{E\to D}$) of exposure E on outcome D on the scale of logarithm odds ratio was equal to

$$\begin{aligned}\beta^{TE}_{E\to D}&=\log(OR^{TE}_{E\to D})\\&=\log\left\{\frac{P(D_e=1)/\{1-P(D_e=1)\}}{P(D_{e*}=1)/\{1-P(D_{e*}=1)\}}\right\}\\&=\text{logit}\{P(D_e=1)\}-\text{logit}\{P(D_{e*}=1)\}\\&=\text{logit}\{P(D=1|e=1)\}-\text{logit}\{P(D=1|e*=0)\}\\&=\text{logit}\left\{\sum_m P(D=1|e=1,m)P(m|e=1)\right\}-\text{logit}\left\{\sum_m P(D=1|e*=0,m)P(m|e*=0)\right\}\end{aligned}$$

The effect estimation ($\hat{\beta}_{ED|M}(m)$) of adjusting for mediator M by logistic regression model was equal to

$$\hat{\beta}_{ED|M}(m)=\text{logit}\left\{\hat{P}(D=1|e=1,m)\right\}-\text{logit}\left\{\hat{P}(D=1|e*=0,m)\right\}$$

where $\hat{P}(D=1|e=1,m)$ denotes the probability of $D=1$ when the exposure E, and mediator M, have been set to level $e=1$, and m, respectively. And $\hat{P}(D=1|e*=0,m)$ denotes the probability of $D=1$ when the exposure E, and mediator M, have been set to level $e*=0$, and m, respectively. The theoretical results of other causal diagrams in Figure 1 have been shown in the supplementary A.

Note that the bias was defined by taking a difference between effect estimation by adjusting for mediator using logistic regression and the total effect of exposure E on outcome D i.e. $bias=E[\hat{\beta}_{ED|M}(m)]-\beta^{TE}_{E\to D}$. We dissected the biases behavior by varying across the effects of $E\to M$ and $M\to D$ in mistakenly adjusting for mediator under the framework of logistic regression model.

Simulation

For peer review only - http://bmjopen.bmj.com/site/about/guidelines.xhtml

Six scenarios are designed to dissect the sensitivity of bias to the variation of the effects of exposure-mediator and mediator-outcome in adjusting for mediators under the framework of logistic regression model, these *DAG*s are shown in Figure 1. We made the following assumptions for the simulation: 1) all variables were binary following a Bernoulli distribution; 2) the effect from parent nodes to their child node were positive and log-linearly additive. Taking Figure 1a as an example, we randomly generated the exposure following a Bernoulli distribution (i.e. let $P(e=1)=\pi$), then, $P_M=\exp(\alpha_0+\beta_1 e)/\{1+\exp(\alpha_0+\beta_1 e)\}$ for calculating the distribution probability of child node *M* from its parent node *E*. Similarly, $P_D=\exp(\alpha_1+\beta_0 e+\beta_2 m)/\{1+\exp(\alpha_1+\beta_0 e+\beta_2 m)\}$ generated the distribution probability of *D*, where the parameters α_0 and α_1 denoted the intercept of *M* and *D* respectively, and effect parameter β_0, β_1, β_2 referred to the effects of the parent node on their corresponding child node using log odds ratio scale.

After generating data, we dissected the biases behavior between the effects of $E \rightarrow M$ and $M \rightarrow D$ in mistakenly adjusting for mediator under logistic regression model. In scenario 1 (Figure 1a), we compared the performances by across varying the effects of $E \rightarrow M$ and $M \rightarrow D$. Similarly, in scenario 2 (Figure 1b), the effects of $E \rightarrow M_1$, $M_1 \rightarrow M_2$ and $M_2 \rightarrow D$ were explored. In scenario 3 (Figure 1c), we dissected the effects of $E \rightarrow M_1$ ($E \rightarrow M_2$) and $M_1 \rightarrow D$ ($M_2 \rightarrow D$). The comparison of scenario 4 (Figure 1d) was the same as scenario 3 (Figure 1c). In scenario 5 (Figure 1e), the effects of $E \rightarrow M$ and $M \rightarrow D$ were excavated. The scenario 6 (Figure 1f) was identical to the scenario 3. We explored the biases in adjusting for mediator under logistic regression model and

For peer review only - http://bmjopen.bmj.com/site/about/guidelines.xhtml

thus identified the sensitivity of biases to the variation of the effects of exposure-mediator and mediator-outcome.

For each of the 6 simulation scenarios, we observed bias performances of varying across distinct effects in adjusting for mediator using logistic regression model with 1000 simulations repetitions. All simulations were conducted using software R from CRAN (http://cran.r-project.org/).

Results

Scenario 1: one single mediator (Figure 1a)

In Figure 1(a), *E* has a direct ($E \rightarrow D$) effect and an indirect ($E \rightarrow M \rightarrow D$) effect on *D*. Figure 2A depicted that the bias of varying across the effect of $E \rightarrow M$ was obviously greater than the bias of varying across the effect of $M \rightarrow D$. That is, the sensitivity of bias to the variation of the effect $E \rightarrow M$ was greater than the effect of $M \rightarrow D$ in adjusting for the mediator *M* using logistic regression model. In particular, if the effect of $E \rightarrow M$ was specified to zero in Figure 2B, *M* was associated with *D* conditional on *E* and unconditionally independent with *E*, *M* became an independent risk factor of the outcome, adjusting for *M* obtained a positive "bias". Such bias was a consequence of non-collapsibility of odds ratio, and the M-conditional ORs must be far from 1 than the unconditional ORs.[31-32] Actually, both adjustment and non-adjustment for *M* should yield unbiased causal effect estimates. Certainly, in this case, both marginal OR and conditional OR obtained from standardization and inverse-probability weighting were equals to total effect.[33] Moreover, Figure 2A indicated that adjusting for mediator *M* was indeed biased to the total effect of the exposure on the outcome.

The total effect ($\beta^{TE}_{E \rightarrow D}$) of exposure *E* on outcome *D* on the scale of logarithm odds ratio was equal to

For peer review only - http://bmjopen.bmj.com/site/about/guidelines.xhtml

$$\beta_{E\to D}^{TE} = \log(OR_{E\to D}^{TE}) = \log\left\{\frac{P(D_e=1)/\{1-P(D_e=1)\}}{P(D_{e*}=1)/\{1-P(D_{e*}=1)\}}\right\}$$
$$= \log\left\{\frac{P(D_e=1)\times\{1-P(D_{e*}=1)\}}{\{1-P(D_e=1)\}\times P(D_{e*}=1)}\right\}$$
$$= \log\left\{\frac{P(D=1\mid e=1)\times P(D=0\mid e^*=0)}{P(D=0\mid e=1)\times P(D=1\mid e^*=0)}\right\}$$
$$= \log\left\{\frac{[\sum_m P(D=1\mid e=1,m)P(m\mid e=1)]\times[\sum_m P(D=0\mid e^*=0,m)P(m\mid e^*=0)]}{[\sum_m P(D=0\mid e=1,m)P(m\mid e=1)]\times[\sum_m P(D=1\mid e^*=0,m)P(m\mid e^*=0)]}\right\}$$

The effect ($\beta_{ED|M}(m)$) of adjusting for mediator M by logistic regression model can be given

$$\beta_{ED|M}(m) = \mathrm{logit}\{P(D=1\mid e=1,m)\} - \mathrm{logit}\{P(D=1\mid e^*=0,m)\}$$
$$= \log\left\{\frac{P(D=1\mid e=1,m)\times\{1-P(D=1\mid e^*=0,m)\}}{\{1-P(D=1\mid e=1,m)\}\times P(D=1\mid e^*=0,m)}\right\}$$
$$= \beta_0$$

β_0 denotes coefficient of the E adjusting for M using logistic regression model. Furthermore, the effect of adjusting for M was equal to the controlled direct effect.[19] Therefore, the bias of adjusting for mediator using logistic regression model could be obtained i.e. $bias = \beta_{ED|M}(m) - \beta_{E\to D}^{TE}$. We added signs to the edges of the directed acyclic graph to indicate the presence of a particular positive or negative effect in the Figure 3. Therefore, we gained $bias < 0$ under the condition of $\beta_1 * \beta_2 > 0$ (the effect $E\to M$ β_1 and the effect $M\to D$ β_2), indicating that the total effect of E on D was biased in adjusting for M using logistic regression model in Figure 3a, Figure 3b, Figure 3e & Figure 3f. And the bias was less than zero when the effect $E\to M$ (β_1) and the effect $M\to D$ (β_2) share same signs. (i.e. both the effects $E\to M$ ($\beta_1 > 0$) and $M\to D$($\beta_2 > 0$) were a positive sign or both the effects $E\to M$ ($\beta_1 < 0$) and $M\to D$($\beta_2 < 0$) were a negative sign). Furthermore, we obtained $bias > 0$, if $\beta_1 * \beta_2 < 0$, suggesting that the total effect of E on D was biased in adjusting for M in

For peer review only - http://bmjopen.bmj.com/site/about/guidelines.xhtml

Figure 3c, Figure 3d, Figure 3g & Figure 3h. And the bias was greater than zero when the signs of the effects $E \rightarrow M$ (β_1) and $M \rightarrow D$ (β_2) were the opposite. The results illustrated that the bias was less than zero under the case of the effects of exposure-mediator and mediator-outcome sharing the same sign; the bias was greater than zero under circumstances of the effects of exposure-mediator and mediator-outcome having opposite signs. We also illustrated the case of the Figure 3c with the effects $E \rightarrow M$ and $E \rightarrow D$ greater than zero, effect $M \rightarrow D$ less than zero in supplementary B. More details of theoretical derivation can be found in Appendix.

Scenario 2: two series mediators (Figure 1b)

Figure 1(b) is a depiction through two series mediators, decomposing total effects into direct effect ($E \rightarrow D$) and indirect effect ($E \rightarrow M_1 \rightarrow M_2 \rightarrow D$). The bias of varying across the effect of $E \rightarrow M_1$ was greater than the one of varying across the effect of $M_2 \rightarrow D$ under adjustment for M_1, M_2 and M_1 M_2 together in Figure 4, respectively. In this situation, the correlation of series mediators was strong enough to avoid M_2 from becoming an independent cause of the outcome.

Scenario 3: two independent parallel mediators (Figure 1c)

Figure 1c shows that the exposure E independently causes M_1 and M_2 and indirectly influences the outcome D through M_1 and M_2, forming three causal paths $E \rightarrow D$, $E \rightarrow M_1 \rightarrow D$ and $E \rightarrow M_2 \rightarrow D$. The results obtained that the bias of varying across the effect of $E \rightarrow M_1$ was considerably greater than the one of varying across the effect of $M_1 \rightarrow D$ under adjustment for M_1 in Figure 5A. However, the bias of varying across the effect of $E \rightarrow M_2$ was nearly equal to the one with varying across the effect of $M_2 \rightarrow D$

For peer review only - http://bmjopen.bmj.com/site/about/guidelines.xhtml

under the identical model of adjustment for M_1 in Figure 5A. Then, an above similar result can be obtained in Figure 5B. In addition, Figure 5C indicated that biases of varying across the effects of $E \rightarrow M_1$ and $E \rightarrow M_2$ were obviously greater than the ones of varying across the effects of $M_1 \rightarrow D$ and $M_2 \rightarrow D$ under simultaneously adjusting for M_1 and M_2.

Scenario 4: two correlated parallel mediators (Figure 1d)

In Figure 1d, there exist five paths from E to D: $E \rightarrow D$, $E \rightarrow M_1 \rightarrow D$, $E \rightarrow M_2 \rightarrow D$, $E \rightarrow M_1 \leftarrow M_2 \rightarrow D$ and $E \rightarrow M_2 \rightarrow M_1 \rightarrow D$. In particular, the path $E \rightarrow M_1 \leftarrow M_2 \rightarrow D$ is a blocked path, due to the M_1 being a collider node. Figure 6A indicated that the bias of varying across the effect of $E \rightarrow M_1$ was obviously greater than the one of varying across the effect of $M_1 \rightarrow D$ under adjustment for M_1. However, the bias of varying across the effect of $E \rightarrow M_2$ was almost equal to the one of varying across the effect of $M_2 \rightarrow D$ under the identical adjustment model. Similarly, an analogous result of biases behavior was shown in Figure 6B. Besides, the biases of varying across the effects of $E \rightarrow M_1$ and $E \rightarrow M_2$ were greater than the ones of varying across the effects of $M_1 \rightarrow D$ and $M_2 \rightarrow D$ in adjusting for M_1 and M_2 in Figure 6C. Simultaneously, the bias was higher sensitive to the variation of effect of $E \rightarrow M_2$ than effect of $E \rightarrow M_1$ under adjustment for M_1 and M_2, which adjusting for the collider node M_1 would partially open the path $E \rightarrow M_1 \leftarrow M_2 \rightarrow D$.

Scenario 5: a single mediator with an unobserved confounder (Figure 1e)

Figure 1e provides a causal diagram representing the relationship among exposure E, outcome D, mediator M and unobserved confounder U. It revealed that the bias of varying across the effect of $E \rightarrow M$ was lower than the one of varying across the effect of $M \rightarrow D$. Unobserved confounder distorts the association between the exposure and

For peer review only - http://bmjopen.bmj.com/site/about/guidelines.xhtml

outcome ($E \leftarrow U \rightarrow D$) in Figure 7.

Scenario 6: two parallel mediators with an unobserved confounder (Figure 1f)

As described above, Figure 1f is a depiction of two parallel mediators M_1 and M_2 with an unobserved confounder U. The bias of varying across the effect of $E \rightarrow M_1$ was obviously less than the one of varying across the effect of $M_1 \rightarrow D$ under the adjustment for M_1 in Figure 8A. However, the bias of varying across the effect of $E \rightarrow M_2$ was greater than the one of varying across the effect of $M_2 \rightarrow D$ under the identical model of adjusting for M_1. A similar result can also obtain in Figure 8B. Besides, biases of varying across the effects of $E \rightarrow M_1$ and t $E \rightarrow M_2$ were distinctly less than the ones of varying across the effects of $M_1 \rightarrow D$ and $M_2 \rightarrow D$ under the common model of adjusting for M_1 and M_2 in Figure 8C.

Application

In this analysis, we evaluated two statistical models (unadjusted and M-adjusted) to assess the effect of diabetes on cardiovascular diseases under the scenario 1. The information of 22900 people were collected from the Health Management Center of Shandong Provincial Hospital (HMCSPH). All individuals were Urban Han Chinese with the age above 20 years old and they took the physical examination in 2013. Many studies focused on the associations diabetes with metabolic syndrome,[34] metabolic syndrome with cardiovascular disease,[35] respectively.

The exposure indicator E takes the value 1 if people suffer from diabetes, and zero otherwise. The outcome D (cardiovascular diseases) takes the value 1 if the people diagnosed with cardiovascular diseases, and takes the value 0 otherwise. The mediator

For peer review only - http://bmjopen.bmj.com/site/about/guidelines.xhtml

M (metabolic syndrome) takes the value 1 if people were the metabolic syndrome and takes the value 0 otherwise. When adjustment for age and gender by using the logistic regression model can obtain the total effect of diabetes E on cardiovascular diseases D being equal to $\beta = 0.598$ (95% confidence interval (*CI*), 0.307~0.877). Then the effect of adjusting for metabolic syndrome M was equal to $\beta_M = 0.429$ (95% confidence interval (*CI*), 0.113~0.736). Therefore, the bias was $\beta_M - \beta = -0.169 < 0$, suggesting that the effect of E on D was underestimated under adjusting for mediator M. This bias can have negative implication on the interpretation of effect of diabetes on cardiovascular. The adjustment for mediator produced biased estimates, and thus adjustment is inappropriate and should be avoided. A particular example was adjustment for time-varying confounders which are also mediators using methods including standardization, inverse-probability weighting, and G-estimation.[36] That is to say, investigators should remember to consider biology and clinical information when specifying a statistical model.

Discussion

In the paper, we dissected the sensitivity of bias to the variation of the effects of exposure-mediator and mediator-outcome in adjusting for mediators under the framework of logistic regression model. In the following four scenarios: a single mediator (Figure 1a in scenario 1), two series mediators (Figure 1b in scenario 2), two independent parallel (Figure 1c in scenario 3) or two correlated parallel mediators (Figure 1d in scenario 4), the bias of varying across the effect of exposure-mediator was greater than the one of varying across the effect mediator-outcome in adjusting for the mediator (Figure 2, Figure 4, Figure 5 & Figure 6). However, in other two

For peer review only - http://bmjopen.bmj.com/site/about/guidelines.xhtml

scenarios: a single mediator or two independent parallel mediators in the presence of unobserved confounders (Figure 1e in scenario 5 & Figure 1f in scenario 6), the biases were higher sensitive to the variation of effect of mediator-outcome than effect of exposure-mediator in adjusting for mediator (Figure 7 & Figure 8).

Conditioning on a mediator is of concern in all areas of epidemiologic researches,[13,19,37] it indeed lead to bias in estimating the total effect of the exposure on the outcome.[8,22-23] Mediators and confounders are indistinguishable in terms of statistical association and conceptual grounds.[3] Most of the studies focus on the mediation effect analysis such as the calculation of direct effect and indirect effect.[20-21,38-41] Recently some authors used causal diagrams described how appropriate handling of the matching variables. And they have proved that matching on mediator *M* renders *M* and *D* independent (by design) in the matched study. Matching on variable that are affected by the exposure and the outcome, or mediators between the exposure and the outcome, would ordinary produce irremediable bias. Furthermore, matching on mediator *M* blocks the causal path $E \rightarrow M \rightarrow D$ and thus produces unfaithfulness for estimating the total effect *E* on *D*.[31,42] Little effort has been made to learn the biases performances in adjusting for mediator in estimating the total effect of exposure on outcome. Our study results revealed that the biases were higher sensitive to the variation of effects of exposure-mediator than effects of mediator-outcome in adjusting for mediator in the absence of the unobserved confounder in causal diagrams (Figure 1a, Figure 1b, Figure 1c & Figure 1d). Nevertheless, for causal diagrams (Figure 1e & Figure 1f), the biases were higher sensitive to the variation of effects of mediator-outcome than effects of exposure-mediator in adjusting for mediator in the presence of the unobserved

For peer review only - http://bmjopen.bmj.com/site/about/guidelines.xhtml

confounder. Therefore, the biases of varying across different effects depended on the causal diagrams framework whether there existed unobserved confounder.

The causal diagrams depicted in Figure 1 are indeed very simplistic and concise, as they all exclude confounding factors of *E* and *M* as well as *M* and *D*. In practical application, there exist some confounders in each pair of *E*, *M*, and *D*. Besides our simulation study was not comprehensive enough to evaluate the bias performances in adjusting for the mediator under logistic regression, because it only considered binary variables, the certain scenarios of effect size and the common type of models. In medical research, regression modeling is commonly used to adjust for covariates associated with both the outcome and exposure. In this paper, the biases are defined by the difference between M-adjusted and unadjusted ORs, some of which is attributable to the non-collapsibility of OR. In the field of causal inference, standardization and inverse-probability weighting may obtain the different bias comparing with the regression modeling, and they may be better alternatives to calculate bias[4-5]. Therefore, in future research, the methods of standardization and inverse-probability weighting could be used to calculate the biases of this paper definition. The work in the further ought to reinforce the mechanisms and conceptual frameworks of confounder and mediator form causal diagrams so as to avoid falling into analytic pitfalls.

Conclusion

In conclusion, the sensitivity of biases to the variation of the effects of exposure-mediator and mediator-outcome were related to whether there is an unobserved confounder in causal diagrams. The biases were higher sensitive to the

For peer review only - http://bmjopen.bmj.com/site/about/guidelines.xhtml

variation of effects of exposure-mediator than effects of mediator-outcome in adjusting for mediator in the absence of unobserved confounders; while the biases were higher sensitive to the variation of effects of mediator-outcome than effects of exposure-mediator in the presence of unobserved confounder.

Statements

Acknowledgments

We would like to thank the anonymous reviewers and academic editors for providing us with constructive comments and suggestions and also wish to acknowledge our colleagues for their invaluable work.

Authors' contributions

TTW and HKL jointly conceived the idea behind the article and designed the study. TTW helped conduct the literature review, performed the simulation and prepared the first draft of the manuscript. PS, YYY, XRS, YL and ZSY participated in the design of the study and the revision of the manuscript. FZX advised on critical revision of the manuscript for important intellectual content. All authors read and approved the final manuscript.

Funding

This work was supported by grants from the National Natural Science Foundation of China (grant number 81573259)

Competing interests

The authors declare that they have no competing interests.

Ethics approval and materials

Ethics Committee of the School of Public Health (20140322), Shandong University.

Written informed consent was obtained from all participants.

Provenance and peer review

Not commissioned; externally peer reviewed.

For peer review only - http://bmjopen.bmj.com/site/about/guidelines.xhtml

Data sharing statement

No additional data are available.

Open Access

This is an Open Access article distributed in accordance with the Creative Commons Attribution Non Commercial (CC BY-NC 4.0) license, which permits others to distribute, remix, adapt, build upon this work noncommercially, and license their derivative works on different terms, provided the original work is properly cited and the use is non-commercial. See: http:// creativecommons.org/licenses/by-nc/4.0/

References

1 Bulterys M, Morgenstern H. Confounding or intermediate effect? An appraisal of iatrogenic bias in perinatal AIDS research. *Paediatr Perinat Epidemiol* 1993;7:387–394.
2 Herna´n MA, Hernandez-Diaz S, Werler MM, et al. Causal knowledge as a prerequisite for confounding evaluation: an application to birth defects epidemiology. *Am J Epidemiol* 2002;155:176–184.
3 MacKinnon DP, Krull JL, and Lockwood CM. Equivalence of the mediation, confounding and suppression effect. *Prevention Science* 2000;1:173–181.
4 Mansournia MA, Altman DG. Inverse probability weighting[J]. *Bmj*, 2016, 352:i189.
5 Gharibzadeh S, Mohammad K, Rahimiforoushani A, Amouzegar A, Mansournia MA. Standardization as a tool for causal inference in medical research. *Arch Iran Med*. 2016;19:666-70.
6 Howards PP, Schisterman EF, Poole C, et al. "Toward a clearer definition of confounding" revisited with directed acyclic graphs, *Am J Epidemiol* 2012;176:506–511.
7 Williamson EJ, Aitken Z, Lawrie J, et al. Introduction to causal diagrams for confounder selection. *Respirology*. 2014;19:303-311.
8 Wil cox AJ, Weinberg CR, Basso O. On the pitfalls of adjusting for gestational age at birth. *Am J Epidemiol*. 2011;174:1062–1068.
9 Psaty BM, Koepsell TD, Lin D, et al. Assessment and control for confounding by indication in observational studies. *J Am Geriatr Soc* 1999; 47:749-54.
10 Tilaki K H. Methodological issues of confounding in analytical epidemiology studies. *Caspian J intern Med* 2012;3:488-495.
11 Kamangar F MD. Confounding variable in epidemiology studies: Basics and beyond. *Arch Tran Med*. 2012;15:508-516.
12 Gaskins AJ, Schisterman EF. The effect of lipid adjustment on the analysis of environmental contaminants and the outcome of human health risks. *Methods Mol Biol* 2009;580:371-381.

For peer review only - http://bmjopen.bmj.com/site/about/guidelines.xhtml

13 Robins JM, Greenland S. Identifiability and exchangeability for direct and indirect effects. *Epidemiology* 1992;3:143–155.
14 Pearl J. Direct and indirect effects. Seventeenth conference of uncertainy in artificial intelligence, 2001. San Francisco, CA: Morgan Kaufmann, 2001.
15 Huang B, Sivaganesan S, Succop P, et al. Statistical assessment of mediational effects for logistic mediational models. *Statist. Med* 2004;23:2713-2728.
16 Valeri L, Coull B A. Estimating causal contrasts involving intermediate variables in the presence of selection bias [J]. *Statistics in Medicine*, 2016, 35(26): 4779-4793.
17 MacKinnon D P, Pirlott A G. Statistical approaches for enhancing causal interpretation of the M to Y relation in mediation analysis [J]. *Personality and Social Psychology Review*, 2015, 19(1): 30-43.
18 Imai K, Keele L, Tingley D. A general approach to causal mediation analysis. *Psychol Methods*. 2010;15(4):309–334.
19 VanderWeele TJ. Bias formulas for sensitivity analysis for direct and indirect effects. *Epidemiology*. 2010;21(4):540–551.
20 VanderWeele TJ, Vansteelandt S. Odds ratios for mediation analysis for a dichotomous outcome. *Am J Epidemiol*. 2010;172(12):1339–1348.
21 Valeri L, VanderWeele TJ. Mediation analysis allowing for exposure-mediator interactions and causal interpretation: theoretical assumptions and implementation with SAS and SPSS macros [J]. *Psychological methods*, 2013, 18(2): 137.
22 Robins J. The control of confounding by intermediate variable. *Statistics in Medicine*. 1989;8:679-701.
23 Schistermana EF, Coleb SR, Platt RW. Overadjustment Bias and Unnecessary Adjustment in Epidemiologic Studies. *Epidemiology*. 2009;20:488–495.
24 Pearl J. Causal diagrams for empirical research. *Biometrika*. 1995;82:669-688.
25 Pearl J. Causality: models, reasoning, and inference. *Econometric Theory*. 2003;19: 675-685.
26 Joffe M, Gambhir M, Chadeau-Hyam M, et al. Causal diagrams in systems epidemiology. *Emerg Themes Epidemiol*. 2012; 9:1-18.
27 Pearl, J. Causality: models, reasoning, and inference. Cambridge: Cambridge University Press; 2000.
28 Pearl J. Causal inference in statistics: an overview. *Statistics Surveys*. 2009; 3:96–146.
29 Rubin DB. Should observational studies be designed to allow lack of balance in covariate distributions across treatment groups? *Statistics in Medicine*. 2009;28:1420-1423.
30 Shpitser I. Counterfactual graphical models for longitudinal mediation analysis with unobserved confounding. *Cognitive Science* 2013;37:1011-1035.
31 Mansournia MA, Hernán MA, Greenland S. Matched designs and causal diagrams. *Int J Epidemiol*. 2013;42:860-869.
32 Greenland S, Robins JM, Pearl J. Confounding and collapsibility in causal inference. *Stat Sci* 1999;14:29–46.

For peer review only - http://bmjopen.bmj.com/site/about/guidelines.xhtml

33 Mansournia MA, Greenland S. The relation of collapsibility and confounding to faithfulness and stability. *Epidemiology*. 2015;26:466-72.

34 Clausen TD, Mathiesen ER, Hansen T, et al. Overweight and the metabolic syndrome in adult offspring of women with diet-treated gestational diabetes mellitus or type 1 diabetes.[J]. *J Clin Endocrinol Metab*, 2013, 94:2464-70.

35 Cheng JY, Wong VW, Tse YK, et al. Metabolic syndrome increases cardiovascular events but not hepatic events and death in patients with chronic hepatitis B[J]. *Hepatology*, 2016, 64:1507.

36 Mansournia MA, Danaei G, Forouzanfar MH, Mahmoudi M, Jamali M, Mansournia N, Mohammad K. Effect of physical activity on functional performance and knee pain in patients with osteoarthritis: analysis with marginal structural models. *Epidemiology*. 2012;23:631-40.

37 VanderWeele TJ, Mumford SL, Schisterman EF. Conditioning on intermediates in perinatal epidemiology. *Epidemiology*. 2012;23(1):1–9.

38 Lange T, Rasmussen M, Thygesen LC. Assessing natural direct and indirect effects through multiple pathways. *American Journal of Epidemiology*. 2013;179, 513–518.

39 Shpitser, Ilya and VanderWeele, Tyler J. "A complete graphical criterion for the adjustment formula in mediation analysis," *The International Journal of Biostatistics*.2011;7,1-24.

40 Albert J M, Wang W. Sensitivity analyses for parametric causal mediation effect estimation [J]. *Biostatistics*, 2015, 16(2): 339-351.

41 Wang W, Zhang B. Assessing natural direct and indirect effects for a continuous exposure and a dichotomous outcome[J]. *Journal of Statistical Theory and Practice*, 2016, 10(3): 574-587.

42 Li H, Yuan Z, Su P, et al. A simulation study on matched case-control designs in the perspective of causal diagrams[J]. *BMC Med Res Methodol*, 2016,16,102 .

Figure 1： Six causal diagrams were designed for estimating the causal effect of *E* on *D*. a) a single mediator *M*; b) two series mediators M_1 and M_2; c) two independent parallel mediators M_1 and M_2; d) two correlated parallel mediators M_1 and M_2; e) a single mediator with an unobserved confounder *U*; f) two independent parallel mediators M_1 and M_2 with an unobserved confounder *U*.

Figure 2： The biases with the effects of $E \rightarrow M$ (red) and $M \rightarrow D$ (blue) increasing, respectively. Comparison of the bias of different effects in adjustment mediator. The OR of target effect (e.g. $E \rightarrow M$) from 1 to 10 given other effects fixed ln2 in Figure 2A. The OR of the effect of $M \rightarrow D$ from 1 to 10 with the effect of $E \rightarrow M$ being equal to zero in Figure 2B (Color figure online).

Figure 3: Illustrating the use of positive and negative signs on edges $E \rightarrow M$, $M \rightarrow D$ and $E \rightarrow D$.

Figure 4: The biases with the effects of $E \rightarrow M_1$ (red), $M_1 \rightarrow M_2$ (blue) and $M_2 \rightarrow D$

For peer review only - http://bmjopen.bmj.com/site/about/guidelines.xhtml

(black) increasing, respectively. Comparison of the bias of different effects in three adjustment models: A) adjustment for M_1, B) adjustment for M_2 and C) adjustment for M_1 and M_2. The OR of target effect (e.g. $E \rightarrow M_1$) from 1 to 10 given the effect of $M_1 \rightarrow M_2$ fixed ln8 and other effects fixed ln2 in Figure 4 (Color figure online).

Figure 5: The biases with the effects of $E \rightarrow M_1$ (red), $E \rightarrow M_2$ (blue), $M_1 \rightarrow D$ (black) and $M_2 \rightarrow D$ (green) increasing, respectively. Comparison of the bias of different effects in three adjustment models: A) adjustment for M_1, B) adjustment for M_2 and C) adjustment for M_1 and M_2. The OR of target effects (e.g. E$\rightarrow M_1$) from 1 to 10 given other edges effects fixed ln2 in Figure 5 (Color figure online).

Figure 6: The biases with the effects of $E \rightarrow M_1$ (red), $E \rightarrow M_2$ (blue), $M_1 \rightarrow D$ (black), $M_2 \rightarrow D$ (green) and the effect of $M_2 \rightarrow M_1$ (purple) increasing, respectively. Comparison of the bias of different effects in three adjustment models: A) adjustment for M_1, B) adjustment for M_2 and C) adjustment for M_1 and M_2. The OR of target effects (e.g. $E \rightarrow M_1$) from 1 to 10 given other effects fixed ln2 in Figure 6 (Color figure online).

Figure 7: The biases with the effects of $E \rightarrow M$ (red) and $M \rightarrow D$ (blue) respectively. Comparison of the bias of different effects in adjustment mediator M. The OR of target effects (e.g. $E \rightarrow M$) from 1 to 10 given the effects of causal edges fixed ln2 and the effect of confounder edges fixed ln5 in Figure ln8 (Color figure online).

Figure 8: The biases with the effects of $E \rightarrow M_1$ (red), $E \rightarrow M_2$ (blue), $M_1 \rightarrow D$ (black) and $M_2 \rightarrow D$ (green) respectively. Comparison of the bias of different effects in three adjustment models: A) adjustment for M_1, B) adjustment for M_2, and C) adjustment for M_1 and M_2. The OR of target effects (e.g. $E \rightarrow M_1$) from 1 to 10 given the effects of causal edges fixed ln2 and the effect of confounder edges fixed ln5 in Figure 8 (Color figure online).

For peer review only - http://bmjopen.bmj.com/site/about/guidelines.xhtml

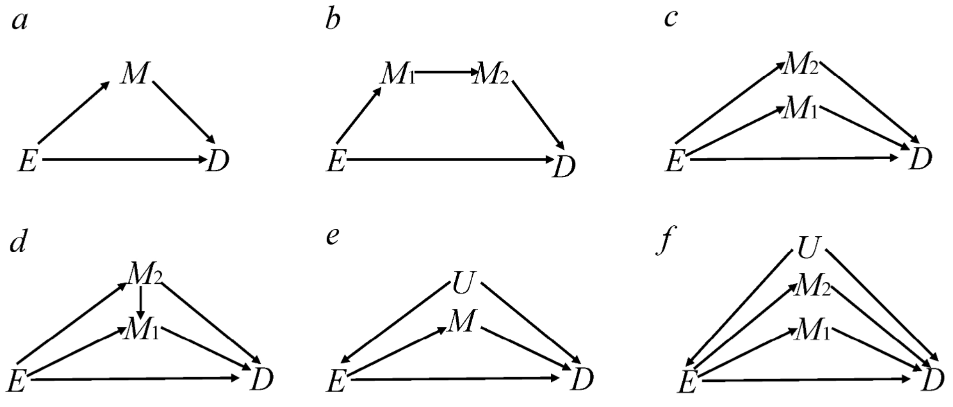

Figure 1: Six causal diagrams were designed for estimating the causal effect of E on‼ ┼ D.

252x110mm (300 x 300 DPI)

For peer review only

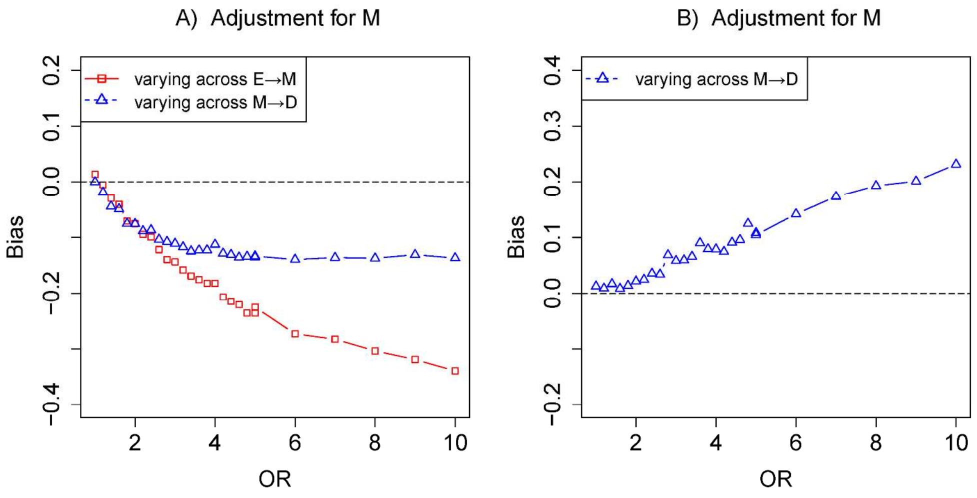

Figure 2 : The biases with the effects E→M (red) and M→D (blue) increasing, respectively.

281x148mm (300 x 300 DPI)

review only

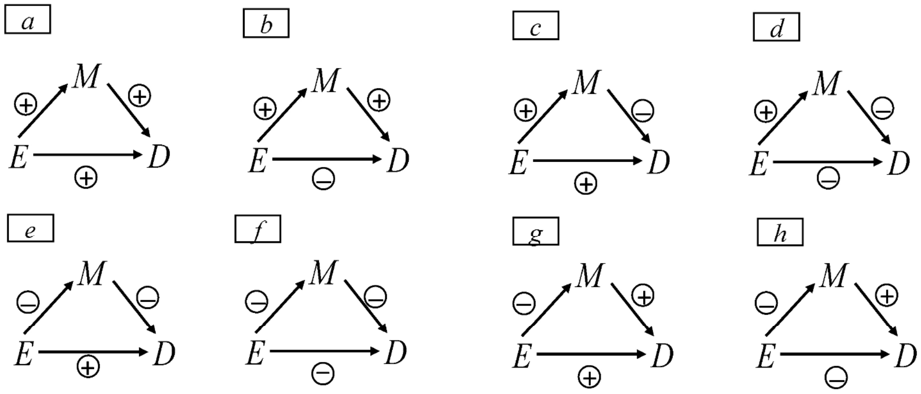

Figure 3: Illustrating the use of positive and negative signs on edges E→M, M→D and E→D.

237x106mm (300 x 300 DPI)

For peer review only

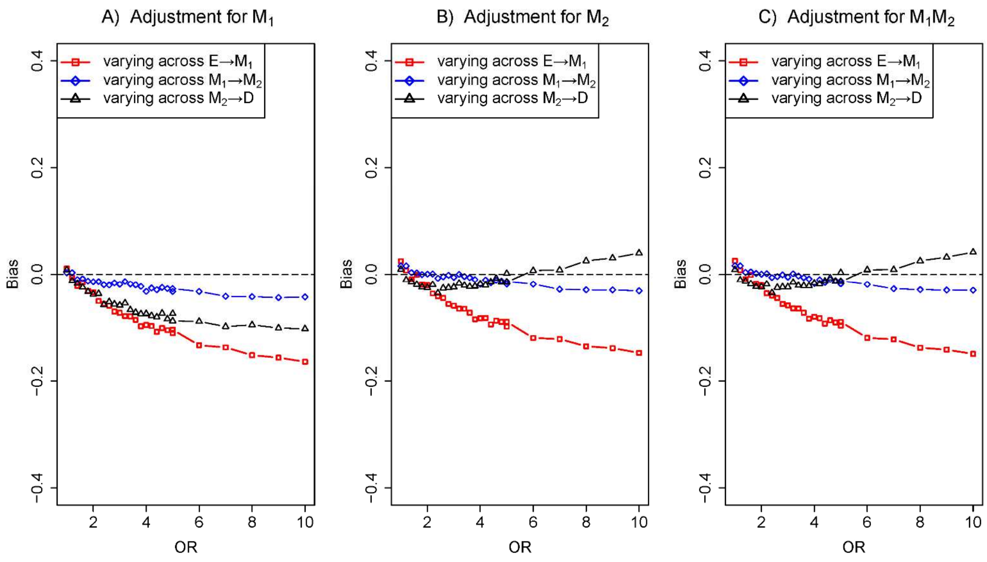

Figure 4: The biases with the effects E→M_1 (red), M_1→M_2 (blue) and M_2→D (black) increasing, respectively.

270x155mm (300 x 300 DPI)

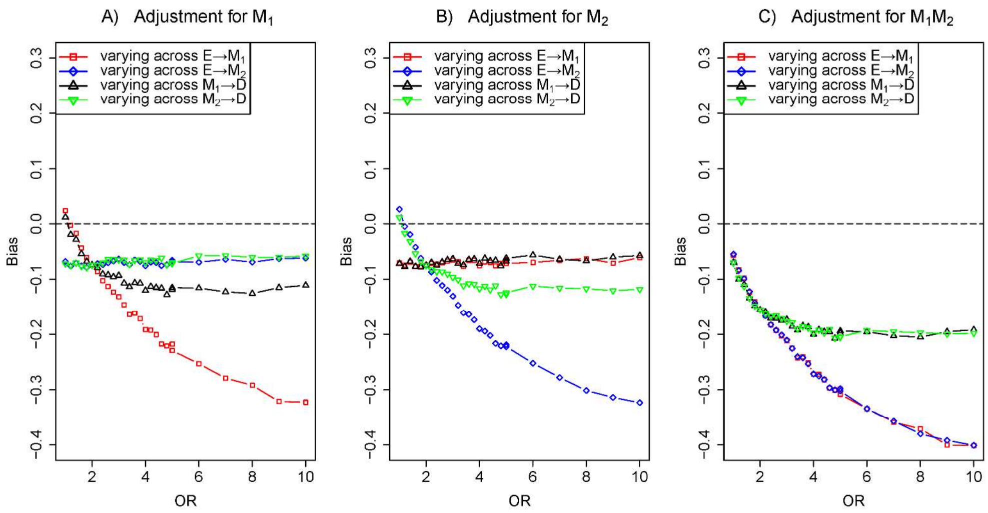

Figure 5 : The biases with the effects E→M_1 (red), E→M_2 (blue), M_1→D (black) and M_2→D (green) increasing, respectively.

279x147mm (300 x 300 DPI)

review only

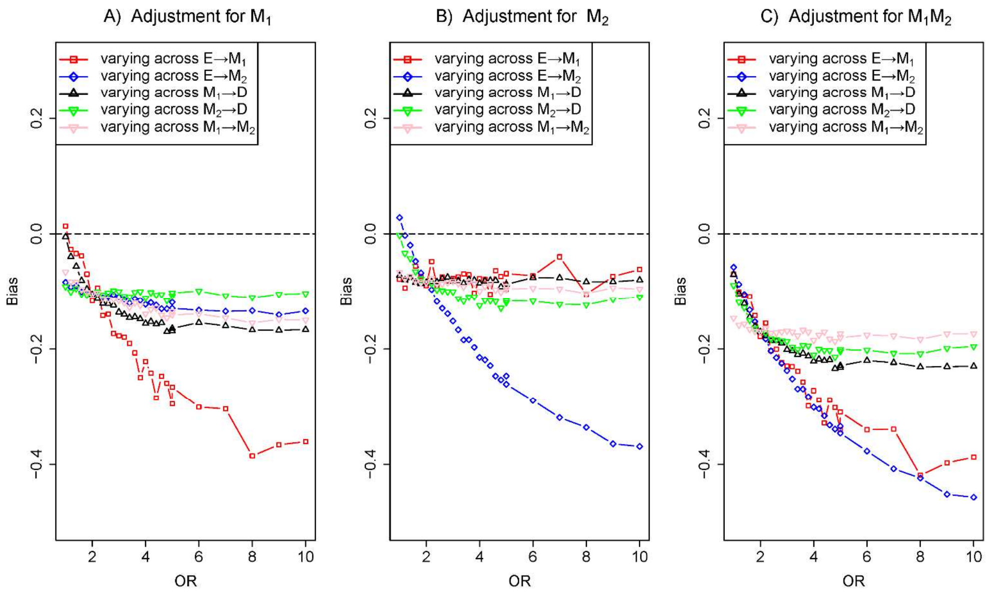

Figure 6: The biases with the effects E→M_1 (red), E→M_2 (blue), M_1 →D (black), M_2 →D (green) and the effect M_2 →M_1 (purple) increasing, respectively.

278x169mm (300 x 300 DPI)

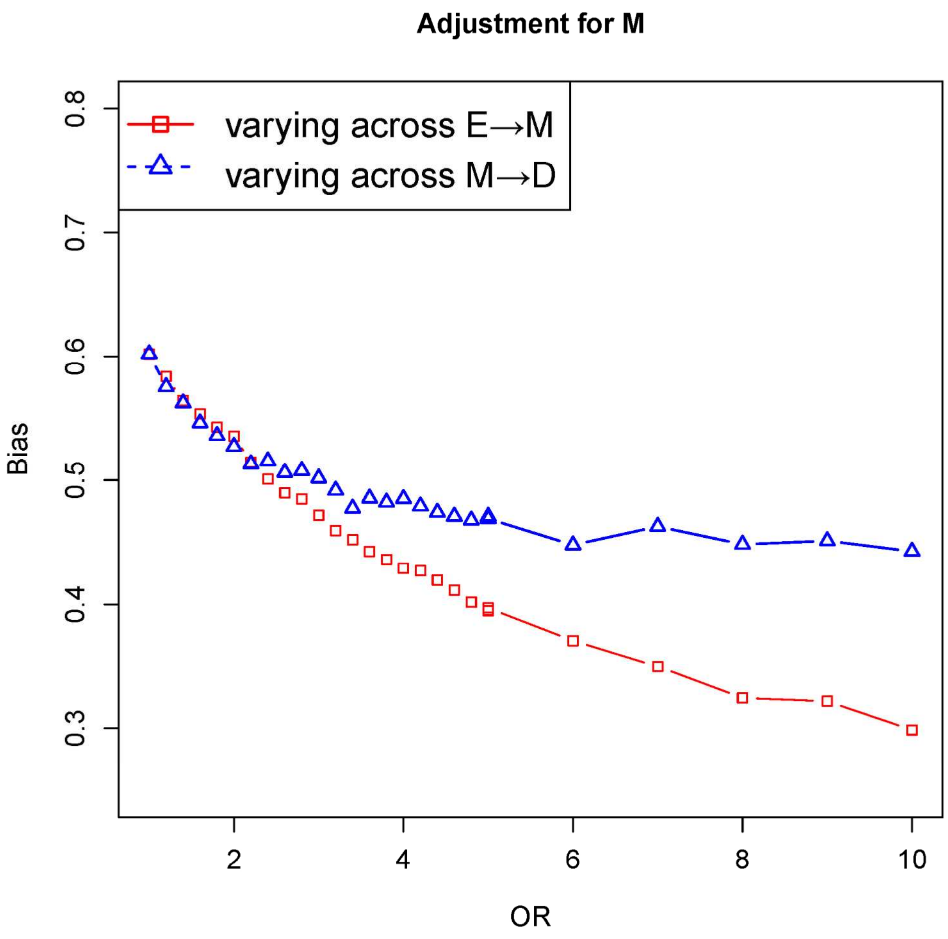

Figure 7: The biases with the effects E→M (red) and M→D (blue) respectively.

177x177mm (300 x 300 DPI)

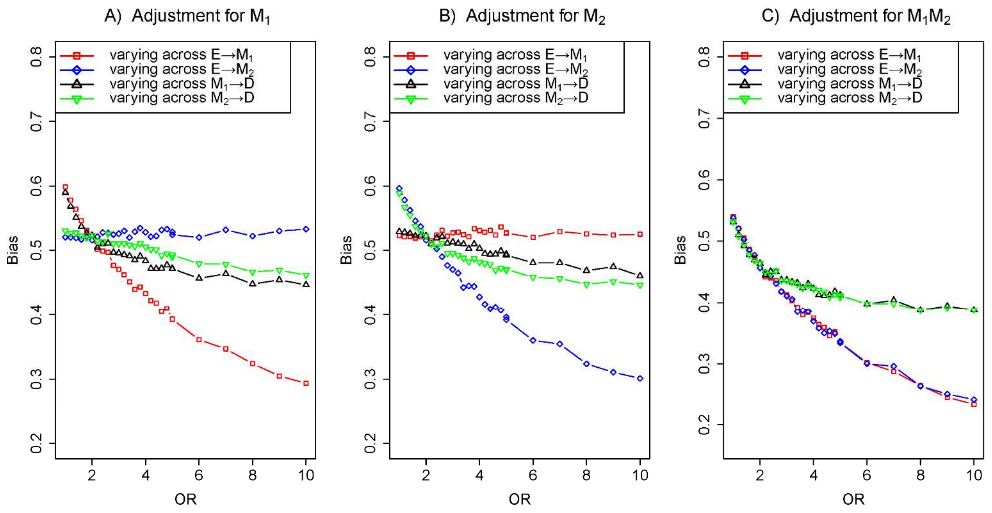

Figure 8 : The biases with the effects E→M_1 (red), E→M_2 (blue), M_1→D (black) and M_2→D (green) respectively.

281x148mm (300 x 300 DPI)

review only

Appendix:

The effect of adjusting for mediator was biased for estimating the total effect of exposure on outcome using logistic regression model. Theoretical derivation of Figure 1a as follow：

Suppose the logistic models among *E*, *M* and *D* are:

$$\text{logit}\{P(D=1|e,m)\}=\alpha_1+\beta_0 e+\beta_2 m,$$

$$\text{logit}\{P(M=1|e)\}=\alpha_0+\beta_1 e.$$

The total effect ($\beta^{TE}_{E\to D}$) of exposure *E* on outcome *D* on the odds ratio ($OR^{TE}_{E\to D}$) scale was equal to

$$\begin{aligned}\beta^{TE}_{E\to D} &= \log(OR^{TE}_{E\to D})\\ &= \log\left\{\frac{P(D_e=1)/\{1-P(D_e=1)\}}{P(D_{e^*}=1)/\{1-P(D_{e^*}=1)\}}\right\}\\ &= \log\left\{\frac{P(D_e=1)\times\{1-P(D_{e^*}=1)\}}{\{1-P(D_e=1)\}\times P(D_{e^*}=1)}\right\}\\ &= \log\left\{\frac{P(D=1|e=1)\times P(D=0|e^*=0)}{P(D=0|e=1)\times P(D=1|e^*=0)}\right\}\\ &= \log\left\{\frac{[\sum_m P(D=1|e=1,m)P(m|e=1)]\times[\sum_m P(D=0|e^*=0,m)P(m|e^*=0)]}{[\sum_m P(D=0|e=1,m)P(m|e=1)]\times[\sum_m P(D=1|e^*=0,m)P(m|e^*=0)]}\right\}\end{aligned}$$

The effect ($\beta_{ED|M}(m)$) of adjusting for mediator *M* by logistic regression model is given

$$\begin{aligned}\beta_{ED|M}(m) &= \text{logit}\{P(D=1|e=1,m)\}-\text{logit}\{P(D=1|e^*=0,m)\}\\ &= \log\left\{\frac{P(D=1|e=1,m)\times P(D=0|e^*=0,m)}{P(D=0|e=1,m)\times P(D=1|e^*=0,m)}\right\}\\ &= \beta_0\end{aligned}$$

Therefore,

For peer review only - http://bmjopen.bmj.com/site/about/guidelines.xhtml

$$
\begin{aligned}
\text{bias} &= \beta_0 - \log(OR_{E\to D}^{TE}) \\
&= \log\left\{\frac{\exp(\beta_0)}{\exp(\beta_0)\dfrac{\exp(\beta_2)\times A_1+\exp(\beta_2)\times B_1+C_1+D_1}{\exp(\beta_2)\times A_1+B_1+\exp(\beta_2)\times C_1+D_1}}\right\} \\
&= \log\left\{\frac{\exp(\beta_2)\times A_1+B_1+\exp(\beta_2)\times C_1+D_1}{\exp(\beta_2)\times A_1+\exp(\beta_2)\times B_1+C_1+D_1}\right\}
\end{aligned}
$$

where

$$
\begin{aligned}
A_1 &= \exp(\beta_1+\alpha_0)\times(1+\exp(\beta_0+\alpha_1))\times\exp(\alpha_0)\times(1+\exp(\alpha_1)) \\
B_1 &= \exp(\beta_1+\alpha_0)\times(1+\exp(\beta_0+\alpha_1))\times(1+\exp(\beta_2+\alpha_1)) \\
C_1 &= (1+\exp(\beta_0+\beta_2+\alpha_1))\times\exp(\alpha_0)\times(1+\exp(\alpha_1)) \\
D_1 &= (1+\exp(\beta_0+\beta_2+\alpha_1))\times(1+\exp(\beta_2+\alpha_1))
\end{aligned}
$$

Focusing on the difference of between $\exp(\beta_2)\times B_1+C_1$ and $B_1+\exp(\beta_2)\times C_1$.

$$
\begin{aligned}
T(\beta_1) &= \exp(\beta_2)\times B_1+C_1-(B_1+\exp(\beta_2)\times C_1) \\
&= \exp(\beta_2)\times(B_1-C_1)-(B_1-C_1) \\
&= (\exp(\beta_2)-1)\times(B_1-C_1) \\
&= (\exp(\beta_2)-1)\times(\exp(\beta_1+\alpha_0)\times(1+\exp(\beta_0+\alpha_1))\times(1+\exp(\beta_2+\alpha_1)) \\
&\quad -(1+\exp(\beta_0+\beta_2+\alpha_1))\times\exp(\alpha_0)\times(1+\exp(\alpha_1))) \\
&= (\exp(\beta_2)-1)\times\exp(\alpha_0)\times[\exp(\beta_1)\times(1+\exp(\beta_0+\alpha_1)\times(1+\exp(\beta_2+\alpha_1)) \\
&\quad -(1+\exp(\beta_0+\beta_2+\alpha_1))\times(1+\exp(\alpha_1))]
\end{aligned}
$$

Then, detailed dissection:

1: $\beta_2=0$, $bias=0$.

2: $\beta_2>0$,

① $\beta_1=0$:(i) $\beta_0=0$, $bias=0$;(ii) $\beta_0>0$, $bias>0$;(iii) $\beta_0<0$, $bias<0$.

② $\beta_1<0$:(i) $\beta_0=0$, $bias>0$;(ii) $\beta_0>0$, $bias>0$;(iii) $\beta_0<0$, $bias>0$.

proof (iii)

$$
\begin{aligned}
T(\beta_1) &= (\exp(\beta_2)-1)\times\exp(\alpha_0) \\
&\quad \times\{\exp(\beta_1)\times[1+\exp(\beta_0+\alpha_1)+\exp(\beta_2+\alpha_1)+\exp(\beta_0+\beta_2+2\alpha_1)] \\
&\quad -[1+\exp(\beta_0+\beta_2+\alpha_1)+\exp(\alpha_1)+\exp(\beta_0+\beta_2+2\alpha_1)]\}
\end{aligned}
$$

when $\beta_0<0$ and $\beta_2>0 \Rightarrow \exp(\beta_0)-1<0 \quad \exp(\beta_2)-1>0$

According to $(a-1)(b-1)=ab-a-b+1$, when $(a-1)(b-1)<0 \Rightarrow ab+1<a+b$

For peer review only - http://bmjopen.bmj.com/site/about/guidelines.xhtml

$$1+\exp(\beta_0+\beta_2+\alpha_1)+\exp(\alpha_1)+\exp(\beta_0+\beta_2+2\alpha_1)$$
$$<1+\exp(\beta_0+\alpha_1)+\exp(\beta_2+\alpha_1)+\exp(\beta_0+\beta_2+2\alpha_1)$$
$$\Rightarrow \exp(\beta_0+\beta_2)+1<\exp(\beta_0)+\exp(\beta_2)$$

when

$$\beta_1<\log\left\{\frac{\exp(\beta_0+\beta_2)+1}{\exp(\beta_0)+\exp(\beta_2)}\right\}<0$$

$$\beta_1<\log\left\{\frac{1+\exp(\beta_0+\beta_2+\alpha_1)+\exp(\alpha_1)+\exp(\beta_0+\beta_2+2\alpha_1)}{1+\exp(\beta_0+\alpha_1)+\exp(\beta_2+\alpha_1)+\exp(\beta_0+\beta_2+2\alpha_1)}\right\}<0$$

$$\Rightarrow \exp(\beta_1)<\frac{1+\exp(\beta_0+\beta_2+\alpha_1)+\exp(\alpha_1)+\exp(\beta_0+\beta_2+2\alpha_1)}{1+\exp(\beta_0+\alpha_1)+\exp(\beta_2+\alpha_1)+\exp(\beta_0+\beta_2+2\alpha_1)}<1$$

$$\begin{aligned}\Rightarrow T(\beta_1)=&(\exp(\beta_2)-1)\times\exp(\alpha_0)\\ &\times\{\exp(\beta_1)\times[1+\exp(\beta_0+\alpha_1)+\exp(\beta_2+\alpha_1)+\exp(\beta_0+\beta_2+2\alpha_1)]\\ &-[1+\exp(\beta_0+\beta_2+\alpha_1)+\exp(\alpha_1)+\exp(\beta_0+\beta_2+2\alpha_1)]\}\\ &<0\end{aligned}$$

Therefore, when $\beta_2>0$, $\beta_1<0$, $\beta_0<0$, then $bias>0$.

③ $\beta_1>0$:(i) $\beta_0=0$, $bias<0$;(ii) $\beta_0<0$, $bias<0$;(iii) $\beta_0>0$, $bias<0$.

proof (iii)

$$\begin{aligned}T(\beta_1)=&(\exp(\beta_2)-1)\times\exp(\alpha_0)\\ &\times\{\exp(\beta_1)\times[1+\exp(\beta_0+\alpha_1)+\exp(\beta_2+\alpha_1)+\exp(\beta_0+\beta_2+2\alpha_1)]\\ &-[1+\exp(\beta_0+\beta_2+\alpha_1)+\exp(\alpha_1)+\exp(\beta_0+\beta_2+2\alpha_1)]\}\end{aligned}$$

when $\beta_0>0$ and $\beta_2>0 \Rightarrow \exp(\beta_0)-1>0 \quad \exp(\beta_2)-1>0$

According to $(a-1)(b-1)=ab-a-b+1$, when $ab>0 \Rightarrow ab+1>a+b$

$$1+\exp(\beta_0+\beta_2+\alpha_1)+\exp(\alpha_1)+\exp(\beta_0+\beta_2+2\alpha_1)$$
$$>1+\exp(\beta_0+\alpha_1)+\exp(\beta_2+\alpha_1)+\exp(\beta_0+\beta_2+2\alpha_1)$$
$$\Rightarrow \exp(\beta_0+\beta_2)+1>\exp(\beta_0)+\exp(\beta_2)$$

when

$$\beta_1>\log\left\{\frac{\exp(\beta_0+\beta_2)+1}{\exp(\beta_0)+\exp(\beta_2)}\right\}>0$$

$$\beta_1>\log\left\{\frac{1+\exp(\beta_0+\beta_2+\alpha_1)+\exp(\alpha_1)+\exp(\beta_0+\beta_2+2\alpha_1)}{1+\exp(\beta_0+\alpha_1)+\exp(\beta_2+\alpha_1)+\exp(\beta_0+\beta_2+2\alpha_1)}\right\}>0$$

$$\Rightarrow \exp(\beta_1)>\frac{1+\exp(\beta_0+\beta_2+\alpha_1)+\exp(\alpha_1)+\exp(\beta_0+\beta_2+2\alpha_1)}{1+\exp(\beta_0+\alpha_1)+\exp(\beta_2+\alpha_1)+\exp(\beta_0+\beta_2+2\alpha_1)}>1$$

For peer review only - http://bmjopen.bmj.com/site/about/guidelines.xhtml

$$\Rightarrow T(\beta_1) = (\exp(\beta_2) - 1) \times \exp(\alpha_0)$$
$$\times \{\exp(\beta_1) \times [1 + \exp(\beta_0 + \alpha_1) + \exp(\beta_2 + \alpha_1) + \exp(\beta_0 + \beta_2 + 2\alpha_1)]$$
$$-[1 + \exp(\beta_0 + \beta_2 + \alpha_1) + \exp(\alpha_1) + \exp(\beta_0 + \beta_2 + 2\alpha_1)]\}$$
$$> 0$$

Therefore, when $\beta_2 > 0$, $\beta_1 > 0$, $\beta_0 > 0$, then $bias < 0$.

3: $\beta_2 < 0$,

① $\beta_1 = 0$:(i) $\beta_0 = 0$, $bias = 0$;(ii) $\beta_0 > 0$, $bias > 0$;(iii) $\beta_0 < 0$, $bias < 0$.

② $\beta_1 < 0$:(i) $\beta_0 = 0$, $bias < 0$;(ii) $\beta_0 < 0$, $bias < 0$;(iii) $\beta_0 > 0$, $bias < 0$.

proof (iii)

$$T(\beta_1) = (\exp(\beta_2) - 1) \times \exp(\alpha_0)$$
$$\times \{\exp(\beta_1) \times [1 + \exp(\beta_0 + \alpha_1) + \exp(\beta_2 + \alpha_1) + \exp(\beta_0 + \beta_2 + 2\alpha_1)]$$
$$-[1 + \exp(\beta_0 + \beta_2 + \alpha_1) + \exp(\alpha_1) + \exp(\beta_0 + \beta_2 + 2\alpha_1)]\}$$

when $\beta_0 > 0$ and $\beta_2 < 0 \Rightarrow \exp(\beta_0) - 1 > 0 \qquad \exp(\beta_2) - 1 < 0$

According to $(a-1)(b-1) = ab - a - b + 1$, when $ab < 0 \Rightarrow ab + 1 < a + b$

$$1 + \exp(\beta_0 + \beta_2 + \alpha_1) + \exp(\alpha_1) + \exp(\beta_0 + \beta_2 + 2\alpha_1)$$
$$< 1 + \exp(\beta_0 + \alpha_1) + \exp(\beta_2 + \alpha_1) + \exp(\beta_0 + \beta_2 + 2\alpha_1)$$
$$\Rightarrow \exp(\beta_0 + \beta_2) + 1 < \exp(\beta_0) + \exp(\beta_2)$$

when

$$\beta_1 < \log\left\{\frac{\exp(\beta_0 + \beta_2) + 1}{\exp(\beta_0) + \exp(\beta_2)}\right\} < 0$$

$$\beta_1 < \log\left\{\frac{1 + \exp(\beta_0 + \beta_2 + \alpha_1) + \exp(\alpha_1) + \exp(\beta_0 + \beta_2 + 2\alpha_1)}{1 + \exp(\beta_0 + \alpha_1) + \exp(\beta_2 + \alpha_1) + \exp(\beta_0 + \beta_2 + 2\alpha_1)}\right\} < 0$$

$$\Rightarrow \exp(\beta_1) < \frac{1 + \exp(\beta_0 + \beta_2 + \alpha_1) + \exp(\alpha_1) + \exp(\beta_0 + \beta_2 + 2\alpha_1)}{1 + \exp(\beta_0 + \alpha_1) + \exp(\beta_2 + \alpha_1) + \exp(\beta_0 + \beta_2 + 2\alpha_1)} < 1$$

$$\Rightarrow T(\beta_1) = (\exp(\beta_2) - 1) \times \exp(\alpha_0)$$
$$\times \{\exp(\beta_1) \times [1 + \exp(\beta_0 + \alpha_1) + \exp(\beta_2 + \alpha_1) + \exp(\beta_0 + \beta_2 + 2\alpha_1)]$$
$$-[1 + \exp(\beta_0 + \beta_2 + \alpha_1) + \exp(\alpha_1) + \exp(\beta_0 + \beta_2 + 2\alpha_1)]\}$$
$$> 0$$

Therefore, when $\beta_2 < 0$, $\beta_1 < 0$, $\beta_0 > 0$, then $bias < 0$.

③ $\beta_1 > 0$:(i) $\beta_0 = 0$, $bias > 0$;(ii) $\beta_0 > 0$, $bias > 0$;(iii) $\beta_0 < 0$, $bias > 0$.

For peer review only

proof (iii)

$$T(\beta_1) = (\exp(\beta_2) - 1) \times \exp(\alpha_0)$$
$$\times \{\exp(\beta_1) \times [1 + \exp(\beta_0 + \alpha_1) + \exp(\beta_2 + \alpha_1) + \exp(\beta_0 + \beta_2 + 2\alpha_1)]$$
$$-[1 + \exp(\beta_0 + \beta_2 + \alpha_1) + \exp(\alpha_1) + \exp(\beta_0 + \beta_2 + 2\alpha_1)]\}$$

when $\beta_0 < 0$ and $\beta_2 < 0 \Rightarrow \exp(\beta_0) - 1 < 0 \quad \exp(\beta_2) - 1 < 0$

According to $(a-1)(b-1) = ab - a - b + 1$, when $ab > 0 \Rightarrow ab + 1 > a + b$

$$1 + \exp(\beta_0 + \beta_2 + \alpha_1) + \exp(\alpha_1) + \exp(\beta_0 + \beta_2 + 2\alpha_1)$$
$$> 1 + \exp(\beta_0 + \alpha_1) + \exp(\beta_2 + \alpha_1) + \exp(\beta_0 + \beta_2 + 2\alpha_1)$$
$$\Rightarrow \exp(\beta_0 + \beta_2) + 1 > \exp(\beta_0) + \exp(\beta_2)$$

when

$$\beta_1 > \log\left\{\frac{\exp(\beta_0 + \beta_2) + 1}{\exp(\beta_0) + \exp(\beta_2)}\right\} > 0$$

$$\beta_1 > \log\left\{\frac{1 + \exp(\beta_0 + \beta_2 + \alpha_1) + \exp(\alpha_1) + \exp(\beta_0 + \beta_2 + 2\alpha_1)}{1 + \exp(\beta_0 + \alpha_1) + \exp(\beta_2 + \alpha_1) + \exp(\beta_0 + \beta_2 + 2\alpha_1)}\right\} > 0$$

$$\Rightarrow \exp(\beta_1) > \frac{1 + \exp(\beta_0 + \beta_2 + \alpha_1) + \exp(\alpha_1) + \exp(\beta_0 + \beta_2 + 2\alpha_1)}{1 + \exp(\beta_0 + \alpha_1) + \exp(\beta_2 + \alpha_1) + \exp(\beta_0 + \beta_2 + 2\alpha_1)} > 1$$

$$\Rightarrow T(\beta_1) = (\exp(\beta_2) - 1) \times \exp(\alpha_0)$$
$$\times \{\exp(\beta_1) \times [1 + \exp(\beta_0 + \alpha_1) + \exp(\beta_2 + \alpha_1) + \exp(\beta_0 + \beta_2 + 2\alpha_1)]$$
$$-[1 + \exp(\beta_0 + \beta_2 + \alpha_1) + \exp(\alpha_1) + \exp(\beta_0 + \beta_2 + 2\alpha_1)]\}$$
$$< 0$$

Therefore, when $\beta_2 < 0$, $\beta_1 > 0$, $\beta_0 < 0$, then $bias > 0$.

In conclusion:

1: $\beta_2 = 0$, $bias = 0$.

2: $\beta_2 \neq 0$, $\beta_1 = 0$:(i) $\beta_0 = 0$, $bias = 0$;(ii) $\beta_0 > 0$, $bias > 0$;(iii) $\beta_0 < 0$, $bias < 0$.

3: (i) $\beta_1\beta_2 > 0$, $bias < 0$. (ii) $\beta_1\beta_2 < 0$, $bias > 0$.

For peer review only - http://bmjopen.bmj.com/site/about/guidelines.xhtml

Supplementary A

The theoretical results of others causal diagrams (Figure 1b-Figure 1f) have been shown in the supplementary of manuscript.

(1) Figure 1(b) is a depiction through two series mediators, decomposing total effects into direct ($E \rightarrow D$) and indirect ($E \rightarrow M_1 \rightarrow M_2 \rightarrow D$) components.

On the odds ratio ($OR^{TE}_{E \rightarrow D}$) scale, the total effect ($\beta^{TE}_{E \rightarrow D} = \log(OR^{TE}_{E \rightarrow D})$), comparing exposure level e with $e*$, we could obtain the total effect:

$$\begin{aligned}\beta^{TE}_{E \rightarrow D} &= \log(OR^{TE}_{E \rightarrow D}) \\ &= \log\left\{\frac{P(D_e = 1)/\{1 - P(D_e = 1)\}}{P(D_{e*} = 1)/\{1 - P(D_{e*} = 1)\}}\right\} \\ &= \log\left\{\frac{P(D_e = 1) \times \{1 - P(D_{e*} = 1)\}}{\{1 - P(D_e = 1)\} \times P(D_{e*} = 1)}\right\} \\ &= \log\left\{\frac{P(D = 1 \mid e = 1) \times P(D = 0 \mid e* = 0)}{P(D = 0 \mid e = 1) \times P(D = 1 \mid e* = 0)}\right\} \\ &= \log\left\{\frac{\xi_1}{\xi_2}\right\}\end{aligned}$$

$$\xi_1 = [\sum_{m_1 m_2} P(D = 1 \mid e = 1, m_2) P(m_2 \mid m_1) P(m_1 \mid e = 1)] \times [\sum_{m_1 m_2} P(D = 0 \mid e* = 0, m_2) P(m_2 \mid m_1) P(m_1 \mid e* = 0)]$$

$$\xi_2 = [\sum_{m_1 m_2} P(D = 0 \mid e = 1, m_2) P(m_2 \mid m_1) P(m_1 \mid e = 1)] \times [\sum_{m_1 m_2} P(D = 1 \mid e* = 0, m_2) P(m_2 \mid m_1) P(m_1 \mid e* = 0)]$$

The effect ($\beta_{ED|M_1}(m_1)$) of adjusting for mediator M_1 by logistic regression model can be given

$$\begin{aligned}\beta_{ED|M_1}(m_1) &= \text{logit}\{P(D = 1 \mid e = 1, m_1)\} - \text{logit}\{P(D = 1 \mid e* = 0, m_1)\} \\ &= \log\left\{\frac{P(D = 1 \mid e = 1, m_1) P(D = 0 \mid e* = 0, m_1)}{P(D = 0 \mid e = 1, m_1) P(D = 1 \mid e* = 0, m_1)}\right\} \\ &= \log\left\{\frac{[\sum_{m_2} P(D = 1 \mid e = 1, m_2) P(m_2 \mid m_1)] \times [\sum_{m_2} P(D = 0 \mid e* = 0, m_2) P(m_2 \mid m_1)]}{[\sum_{m_2} P(D = 0 \mid e = 1, m_2) P(m_2 \mid m_1)] \times [\sum_{m_2} P(D = 1 \mid e* = 0, m_2) P(m_2 \mid m_1)]}\right\}\end{aligned}$$

The effect ($\beta_{ED|M_2}(m_2)$) of adjusting for mediator M_2 by logistic regression model can be given

$$\begin{aligned}&\beta_{ED|M_2}(m_2) \\ &= \text{logit}\{P(D = 1 \mid e = 1, m_2)\} - \text{logit}\{P(D = 1 \mid e* = 0, m_2)\} \\ &= \log\left\{\frac{P(D = 1 \mid e = 1, m_2) P(D = 0 \mid e* = 0, m_2)}{P(D = 0 \mid e = 1, m_2) P(D = 1 \mid e* = 0, m_2)}\right\}\end{aligned}$$

For peer review only

The effect ($\beta_{ED|M_1,M_2}(m_1,m_2)$) of adjusting for mediator M_1 M_2 by logistic regression model can be given

$$\beta_{ED|M_1,M_2}(m_1,m_2) = \text{logit}\{P(D=1|e=1,m_1,m_2)\} - \text{logit}\{P(D=1|e^*=0,m_1,m_2)\}$$
$$= \log\left\{\frac{P(D=1|e=1,m_1,m_2)P(D=0|e^*=0,m_1,m_2)}{P(D=0|e=1,m_1,m_2)P(D=1|e^*=0,m_1,m_2)}\right\}$$
$$= \log\left\{\frac{P(D=1|e=1,m_2)P(D=0|e^*=0,m_2)}{P(D=0|e=1,m_2)P(D=1|e^*=0,m_2)}\right\}$$

Therefore, we could evaluate the biases that contains three adjustment models: A) adjustment for M_1, $bias(m_1) = \beta_{ED|M_1}(m_1) - \beta^{TE}_{E\to D}$; B) adjustment for M_2, $bias(m_2) = \beta_{ED|M_2}(m_2) - \beta^{TE}_{E\to D}$ and C) adjustment for M_1 and M_2, $bias(m_1,m_2) = \beta_{ED|M_1,M_2}(m_1,m_2) - \beta^{TE}_{E\to D}$.

(2) Figure 1c shows that the exposure E independently causes M_1 and M_2 and indirectly influences the outcome D through M_1 and M_2, forming three causal paths $E\to D$, $E\to M_1\to D$ and $E\to M_2\to D$.

On the odds ratio ($OR^{TE}_{E\to D}$) scale, the total effect ($\beta^{TE}_{E\to D} = \log(OR^{TE}_{E\to D})$), comparing exposure level e with e^*, we could obtain the total effect:

$$\beta^{TE}_{E\to D} = \log(OR^{TE}_{E\to D})$$
$$= \log\left\{\frac{P(D_e=1)/\{1-P(D_e=1)\}}{P(D_{e^*}=1)/\{1-P(D_{e^*}=1)\}}\right\}$$
$$= \log\left\{\frac{P(D_e=1)\times\{1-P(D_{e^*}=1)\}}{\{1-P(D_e=1)\}\times P(D_{e^*}=1)}\right\}$$
$$= \log\left\{\frac{P(D=1|e=1)\times P(D=0|e^*=0)}{P(D=0|e=1)\times P(D=1|e^*=0)}\right\}$$
$$= \log\left\{\frac{\xi_1}{\xi_2}\right\}$$

$$\xi_1 = [\sum_{m_1m_2} P(D=1|e=1,m_1,m_2)P(m_2|e=1)P(m_1|e=1)]\times[\sum_{m_1m_2} P(D=0|e^*=0,m_1,m_2)P(m_2|e^*=0)P(m_1|e^*=0)]$$
$$\xi_2 = [\sum_{m_1m_2} P(D=0|e=1,m_1,m_2)P(m_2|e=1)P(m_1|e=1)]\times[\sum_{m_1m_2} P(D=1|e^*=0,m_1,m_2)P(m_2|e^*=0)P(m_1|e^*=0)]$$

The effect ($\beta_{ED|M_1}(m_1)$) of adjusting for mediator M_1 by logistic regression model can be given

For peer review only

$$\beta_{ED|M_1}(m_1) = \text{logit}\{P(D=1|e=1,m_1)\} - \text{logit}\{P(D=1|e^*=0,m_1)\}$$

$$= \log\left\{\frac{P(D=1|e=1,m_1)P(D=0|e^*=0,m_1)}{P(D=0|e=1,m_1)P(D=1|e^*=0,m_1)}\right\}$$

$$= \log\left\{\frac{[\sum_{m_2} P(D=1|e=1,m_1,m_2)P(m_2|e=1)]\times[\sum_{m_2} P(D=0|e^*=0,m_1,m_2)P(m_2|e^*=0)]}{[\sum_{m_2} P(D=0|e=1,m_1,m_2)P(m_2|e=1)]\times[\sum_{m_2} P(D=1|e^*=0,m_1,m_2)P(m_2|e^*=0)]}\right\}$$

The effect ($\beta_{ED|M_2}(m_2)$) of adjusting for mediator M_2 by logistic regression model can be given

$$\beta_{ED|M_2}(m_2) = \text{logit}\{P(D=1|e=1,m_2)\} - \text{logit}\{P(D=1|e^*=0,m_2)\}$$

$$= \log\left\{\frac{P(D=1|e=1,m_2)P(D=0|e^*=0,m_2)}{P(D=0|e=1,m_2)P(D=1|e^*=0,m_2)}\right\}$$

$$= \log\left\{\frac{[\sum_{m_1} P(D=1|e=1,m_1,m_2)P(m_1|e=1)]\times[\sum_{m_1} P(D=0|e^*=0,m_1,m_2)P(m_1|e^*=0)]}{[\sum_{m_1} P(D=0|e=1,m_1,m_2)P(m_1|e=1)]\times[\sum_{m_1} P(D=1|e^*=0,m_1,m_2)P(m_1|e^*=0)]}\right\}$$

The effect ($\beta_{ED|M_1,M_2}(m_1,m_2)$) of adjusting for mediator M_1 M_2 by logistic regression model can be given

$$\beta_{ED|M_1,M_2}(m_1,m_2) = \text{logit}\{P(D=1|e=1,m_1,m_2)\} - \text{logit}\{P(D=1|e^*=0,m_1,m_2)\}$$

$$= \log\left\{\frac{P(D=1|e=1,m_1,m_2)P(D=0|e^*=0,m_1,m_2)}{P(D=0|e=1,m_1,m_2)P(D=1|e^*=0,m_1,m_2)}\right\}$$

Therefore, we could evaluate the biases that contains three adjustment models: A) adjustment for M_1, $bias(m_1) = \beta_{ED|M_1}(m_1) - \beta^{TE}_{E\to D}$; B) adjustment for M_2, $bias(m_2) = \beta_{ED|M_2}(m_2) - \beta^{TE}_{E\to D}$ and C) adjustment for M_1 and M_2, $bias(m_1,m_2) = \beta_{ED|M_1,M_2}(m_1,m_2) - \beta^{TE}_{E\to D}$.

(3) In Figure 1d, there exists five paths from E to D: $E\to D$, $E\to M_1\to D$, $E\to M_2\to D$, $E\to M_1\leftarrow M_2\to D$ and $E\to M_2\to M_1\to D$. In particular, the path $E\to M_1\leftarrow M_2\to D$ is a blocked path, due to the M_1 being a collider node.

On the odds ratio ($OR^{TE}_{E\to D}$) scale, the total effect ($\beta^{TE}_{E\to D} = \log(OR^{TE}_{E\to D})$), comparing exposure level e with e^*, we could obtain the total effect:

For peer review only

$$\beta^{TE}_{E\to D} = \log(OR^{TE}_{E\to D})$$
$$= \log\left\{\frac{P(D_e=1)/\{1-P(D_e=1)\}}{P(D_{e*}=1)/\{1-P(D_{e*}=1)\}}\right\}$$
$$= \log\left\{\frac{P(D_e=1)\times\{1-P(D_{e*}=1)\}}{\{1-P(D_e=1)\}\times P(D_{e*}=1)}\right\}$$
$$= \log\left\{\frac{P(D=1\mid e=1)\times P(D=0\mid e*=0)}{P(D=0\mid e=1)\times P(D=1\mid e*=0)}\right\}$$
$$= \log\left\{\frac{\xi_1}{\xi_2}\right\}$$

$$\xi_1 = [\sum_{m_1 m_2} P(D=1\mid e=1,m_1,m_2)P(m_2\mid e=1)P(m_1\mid e=1,m_2)]$$
$$\times[\sum_{m_1 m_2} P(D=0\mid e*=0,m_1,m_2)P(m_2\mid e*=0)P(m_1\mid e*=0,m_2)]$$
$$\xi_2 = [\sum_{m_1 m_2} P(D=0\mid e=1,m_1,m_2)P(m_2\mid e=1)P(m_1\mid e=1,m_2)]$$
$$\times[\sum_{m_1 m_2} P(D=1\mid e*=0,m_1,m_2)P(m_2\mid e*=0)P(m_1\mid e*=0,m_2)]$$

The effect ($\beta_{ED|M_1}(m_1)$) of adjusting for mediator M_1 by logistic regression model can be given

$$\beta_{ED|M_1}(m_1) = \text{logit}\{P(D=1\mid e=1,m_1)\} - \text{logit}\{P(D=1\mid e*=0,m_1)\}$$
$$= \log\left\{\frac{P(D=1\mid e=1,m_1)P(D=0\mid e*=0,m_1)}{P(D=0\mid e=1,m_1)P(D=1\mid e*=0,m_1)}\right\}$$
$$= \log\left\{\frac{[\sum_{m_2} P(D=1\mid e=1,m_1,m_2)P(m_2\mid e=1,m_1)]\times[\sum_{m_2} P(D=0\mid e*=0,m_1,m_2)P(m_2\mid e*=0,m_1)]}{[\sum_{m_2} P(D=0\mid e=1,m_1,m_2)P(m_2\mid e=1,m_1)]\times[\sum_{m_2} P(D=1\mid e*=0,m_1,m_2)P(m_2\mid e*=0,m_1)]}\right\}$$
$$= \log\left\{\frac{\xi_1}{\xi_2}\right\}$$

$$\xi_1 = [\sum_{m_2} P(D=1\mid e=1,m_1,m_2)\frac{P(m_1\mid e=1,m_2)P(m_2\mid e=1)}{\sum_{m_2} P(m_1\mid e=1,m_2)P(m_2\mid e=1)}]$$
$$\times[\sum_{m_2} P(D=0\mid e*=0,m_1,m_2)\frac{P(m_1\mid e*=0,m_2)P(m_2\mid e*=0)}{\sum_{m_2} P(m_1\mid e*=0,m_2)P(m_2\mid e*=0)}]$$
$$\xi_2 = [\sum_{m_2} P(D=0\mid e=1,m_1,m_2)\frac{P(m_1\mid e=1,m_2)P(m_2\mid e=1)}{\sum_{m_2} P(m_1\mid e=1,m_2)P(m_2\mid e=1)}]$$
$$\times[\sum_{m_2} P(D=1\mid e*=0,m_1,m_2)\frac{P(m_1\mid e*=0,m_2)P(m_2\mid e*=0)}{\sum_{m_2} P(m_1\mid e*=0,m_2)P(m_2\mid e*=0)}]$$

The effect ($\beta_{ED|M_2}(m_2)$) of adjusting for mediator M_2 by logistic regression model can be given

For peer review only

$$\beta_{ED|M_2}(m_2) = \text{logit}\{P(D=1|e=1,m_2)\} - \text{logit}\{P(D=1|e^*=0,m_2)\}$$
$$= \log\left\{\frac{P(D=1|e=1,m_2)P(D=0|e^*=0,m_2)}{P(D=0|e=1,m_2)P(D=1|e^*=0,m_2)}\right\}$$
$$= \log\left\{\frac{[\sum_{m_1} P(D=1|e=1,m_1,m_2)P(m_1|e=1,m_2)]\times[\sum_{m_1} P(D=0|e^*=0,m_1,m_2)P(m_1|e^*=0,m_2)]}{[\sum_{m_1} P(D=0|e=1,m_1,m_2)P(m_1|e=1,m_2)]\times[\sum_{m_1} P(D=1|e^*=0,m_1,m_2)P(m_1|e^*=0,m_2)]}\right\}$$

The effect ($\beta_{ED|M_1,M_2}(m_1,m_2)$) of adjusting for mediator M_1 M_2 by logistic regression model can be given

$$\beta_{ED|M_1,M_2}(m_1,m_2) = \text{logit}\{P(D=1|e=1,m_1,m_2)\} - \text{logit}\{P(D=1|e^*=0,m_1,m_2)\}$$
$$= \log\left\{\frac{P(D=1|e=1,m_1,m_2)P(D=0|e^*=0,m_1,m_2)}{P(D=0|e=1,m_1,m_2)P(D=1|e^*=0,m_1,m_2)}\right\}$$

Therefore, we could evaluate the biases that contains three adjustment models: A) adjustment for M_1, $bias(m_1) = \beta_{ED|M_1}(m_1) - \beta^{TE}_{E\to D}$; B) adjustment for M_2, $bias(m_2) = \beta_{ED|M_2}(m_2) - \beta^{TE}_{E\to D}$ and C) adjustment for M_1 and M_2, $bias(m_1,m_2) = \beta_{ED|M_1,M_2}(m_1,m_2) - \beta^{TE}_{E\to D}$.

(4) In Figure 1e, the causal diagrams contained a confounder of exposure-outcome relationship. On the odds ratio ($OR^{TE}_{E\to D}$) scale, the total effect ($\beta^{TE}_{E\to D} = \log(OR^{TE}_{E\to D})$), comparing exposure level *e* with *e**, we could obtain the total effect:

$$\beta^{TE}_{E\to D} = \log(OR^{TE}_{E\to D})$$
$$= \log\left\{\frac{P(D_e=1)/\{1-P(D_e=1)\}}{P(D_{e^*}=1)/\{1-P(D_{e^*}=1)\}}\right\}$$
$$= \log\left\{\frac{P(D_e=1)\times\{1-P(D_{e^*}=1)\}}{\{1-P(D_e=1)\}\times P(D_{e^*}=1)}\right\}$$
$$= \log\left\{\frac{P(D=1|e=1)\times P(D=0|e^*=0)}{P(D=0|e=1)\times P(D=1|e^*=0)}\right\}$$
$$= \log\left\{\frac{[\sum_{mu} P(D=1|e=1,m,u)P(m|e=1)P(u)]\times[\sum_{mu} P(D=0|e^*=0,m,u)P(m|e^*=0)P(u)]}{[\sum_{mu} P(D=0|e=1,m,u)P(m|e=1)P(u)]\times[\sum_{mu} P(D=1|e^*=0,m,u)P(m|e^*=0)P(u)]}\right\}$$

The effect ($\beta_{ED|M}(m)$) of adjusting for mediator M by logistic regression model can be given

For peer review only

$$\beta_{ED|M}(m) = \log it(P(D=1|e=1,m)) - \log it(P(D=1|e^*=0,m))$$

$$= \log\left\{\frac{P(D=1|e=1,m)\times P(D=0|e^*=0,m)}{P(D=0|e=1,m)\times P(D=1|e^*=0,m)}\right\}$$

$$= \log\left\{\frac{[\sum_u P(D=1|e=1,m,u)p(u|e=1,m)]\times[\sum_u P(D=0|e^*=0,m,u)p(u|e^*=0,m)]}{[\sum_u P(D=0|e=1,m,u)p(u|e=1,m)]\times[\sum_u P(D=1|e^*=0,m,u)p(u|e^*=0,m)]}\right\}$$

$$= \log\left\{\frac{[\sum_u P(D=1|e=1,m,u)\frac{p(e=1|u)p(u)}{\sum_u p(e=1|u)p(u)}]\times[\sum_u P(D=0|e^*=0,m,u)\frac{p(e^*=0|u)p(u)}{\sum_u p(e^*=0|u)p(u)}]}{[\sum_u P(D=0|e=1,m,u)\frac{p(e=1|u)p(u)}{\sum_u p(e=1|u)p(u)}]\times[\sum_u P(D=1|e^*=0,m,u)\frac{p(e^*=0|u)p(u)}{\sum_u p(e^*=0|u)p(u)}]}\right\}$$

Therefore, we could evaluate the biases of adjustment models: $bias(m) = \beta_{ED|M}(m) - \beta_{E\to D}^{TE}$.

(5) Figure 1f is a depiction of two parallel mediators M_1 and M_2 with confounder.

On the odds ratio ($OR_{E\to D}^{TE}$) scale, the total effect ($\beta_{E\to D}^{TE} = \log(OR_{E\to D}^{TE})$), comparing exposure level *e* with *e**, we could obtain the total effect:

$$\beta_{E\to D}^{TE} = \log(OR_{E\to D}^{TE})$$

$$= \log\left\{\frac{P(D_e=1)/\{1-P(D_e=1)\}}{P(D_{e^*}=1)/\{1-P(D_{e^*}=1)\}}\right\}$$

$$= \log\left\{\frac{P(D_e=1)\times\{1-P(D_{e^*}=1)\}}{\{1-P(D_e=1)\}\times P(D_{e^*}=1)}\right\}$$

$$= \log\left\{\frac{P(D=1|e=1)\times P(D=0|e^*=0)}{P(D=0|e=1)\times P(D=1|e^*=0)}\right\}$$

$$= \log\left\{\frac{\xi_1}{\xi_2}\right\}$$

$$\xi_1 = [\sum_{m_1 m_2 u} P(D=1|e=1,m_1,m_2,u)P(m_2|e=1)P(m_1|e=1)P(u)]$$
$$\times[\sum_{m_1 m_2 u} P(D=0|e^*=0,m_1,m_2,u)P(m_2|e^*=0)P(m_1|e^*=0)P(u)]$$

$$\xi_2 = [\sum_{m_1 m_2 u} P(D=0|e=1,m_1,m_2,u)P(m_2|e=1)P(m_1|e=1)P(u)]$$
$$\times[\sum_{m_1 m_2 u} P(D=1|e^*=0,m_1,m_2,u)P(m_2|e^*=0)P(m_1|e^*=0)P(u)]$$

The effect ($\beta_{ED|M_1}(m_1)$) of adjusting for mediator M_1 by logistic regression model can be given

For peer review only

$$\beta_{ED|M_1}(m_1) = \text{logit}\{P(D=1|e=1,m_1)\} - \text{logit}\{P(D=1|e^*=0,m_1)\}$$
$$= \log\left\{\frac{P(D=1|e=1,m_1)P(D=0|e^*=0,m_1)}{P(D=0|e=1,m_1)P(D=1|e^*=0,m_1)}\right\}$$
$$= \log\left\{\frac{\xi_1}{\xi_2}\right\}$$

$$\xi_1 = [\sum_{m_2 u} P(D=1|e=1,m_1,m_2,u)\frac{P(m_2|e=1)P(e=1|u)P(u)}{\sum_u P(e=1|u)P(u)}]$$
$$\times[\sum_{m_2 u} P(D=0|e^*=0,m_1,m_2,u)\frac{P(m_2|e^*=0)P(e^*=0|u)P(u)}{\sum_u P(e^*=0|u)P(u)}]$$
$$\xi_2 = [\sum_{m_2 u} P(D=0|e=1,m_1,m_2,u)\frac{P(m_2|e=1)P(e=1|u)P(u)}{\sum_u P(e=1|u)P(u)}]$$
$$\times[\sum_{m_2 u} P(D=1|e^*=0,m_1,m_2,u)\frac{P(m_2|e^*=0)P(e^*=0|u)P(u)}{\sum_u P(e^*=0|u)P(u)}]$$

The effect ($\beta_{ED|M_2}(m_2)$) of adjusting for mediator M_2 by logistic regression model can be given

$$\beta_{ED|M_2}(m_2) = \text{logit}\{P(D=1|e=1,m_2)\} - \text{logit}\{P(D=1|e^*=0,m_2)\}$$
$$= \log\left\{\frac{P(D=1|e=1,m_2)P(D=0|e^*=0,m_2)}{P(D=0|e=1,m_2)P(D=1|e^*=0,m_2)}\right\}$$
$$= \log\left\{\frac{\xi_1}{\xi_2}\right\}$$

$$\xi_1 = [\sum_{m_1 u} P(D=1|e=1,m_1,m_2,u)\frac{P(m_1|e=1)P(e=1|u)P(u)}{\sum_u P(e=1|u)P(u)}]$$
$$\times[\sum_{m_1 u} P(D=0|e^*=0,m_1,m_2,u)\frac{P(m_1|e^*=0)P(e^*=0|u)P(u)}{\sum_u P(e^*=0|u)P(u)}]$$
$$\xi_2 = [\sum_{m_1 u} P(D=0|e=1,m_1,m_2,u)\frac{P(m_1|e=1)P(e=1|u)P(u)}{\sum_u P(e=1|u)P(u)}]$$
$$\times[\sum_{m_1 u} P(D=1|e^*=0,m_1,m_2,u)\frac{P(m_1|e^*=0)P(e^*=0|u)P(u)}{\sum_u P(e^*=0|u)P(u)}]$$

The effect ($\beta_{ED|M_1,M_2}(m_1,m_2)$) of adjusting for mediator M_1 M_2 by logistic regression model can be given

For peer review only

$$
\begin{aligned}
&\beta_{ED|M_1,M_2}(m_1,m_2)\\
&=\text{logit}\{P(D=1|e=1,m_1,m_2)\}-\text{logit}\{P(D=1|e^*=0,m_1,m_2)\}\\
&=\log\left\{\frac{P(D=1|e=1,m_1,m_2)P(D=0|e^*=0,m_1,m_2)}{P(D=0|e=1,m_1,m_2)P(D=1|e^*=0,m_1,m_2)}\right\}\\
&=\log\left\{\frac{[\sum_u P(D=1|e=1,m_1,m_2,u)\frac{P(e=1|u)P(u)}{\sum_u P(e=1|u)P(u)}]\times[\sum_u P(D=0|e^*=0,m_1,m_2,u)\frac{P(e=1|u)P(u)}{\sum_u P(e=1|u)P(u)}]}{[\sum_u P(D=0|e=1,m_1,m_2,u)\frac{P(e=1|u)P(u)}{\sum_u P(e=1|u)P(u)}]\times[\sum_u P(D=1|e^*=0,m_1,m_2,u)\frac{P(e=1|u)P(u)}{\sum_u P(e=1|u)P(u)}]}\right\}
\end{aligned}
$$

Therefore, we could evaluate the biases that contains three adjustment models: A) adjustment for M_1, $bias(m_1)=\beta_{ED|M_1}(m_1)-\beta^{TE}_{E\to D}$; B) adjustment for M_2, $bias(m_2)=\beta_{ED|M_2}(m_2)-\beta^{TE}_{E\to D}$ and C) adjustment for M_1 and M_2, $bias(m_1,m_2)=\beta_{ED|M_1,M_2}(m_1,m_2)-\beta^{TE}_{E\to D}$.

Supplementary B

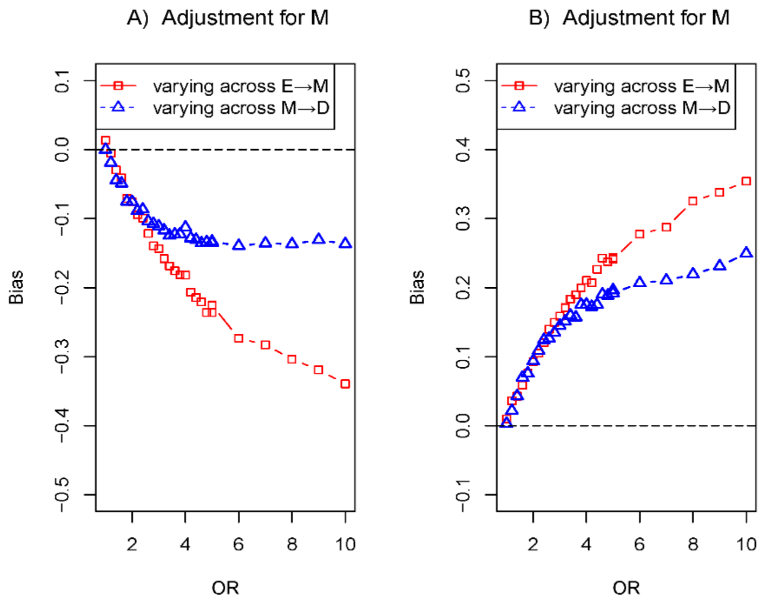

Figure S1: The biases with the effects $E\to M$ (red) and $M\to D$ (blue) increasing, respectively. Comparison of the bias of different effects in adjustment mediator.

The Figure S1-A obtained the result $bias<0$ in Figure 3a with the effects $E\to M$, $M\to D$ and $E\to D$ fixing to ln2. The Figure S1-B gained the result $bias>0$ in Figure 3c with the effects $E\to M$ and $E\to D$ fixing to ln2, effect $M\to D$ fixing to $-\ln 2$. We could obtain the bias performances of varying across the effects of exposure-mediator and mediator-outcome. The effect $E\to M$ of varying across was more sensitive than the effect $M\to D$ of varying across in Figure S1.

STROBE 2007 (v4) checklist of items to be included in reports of observational studies in epidemiology*
Checklist for cohort, case-control, and cross-sectional studies (combined)

Section/Topic	Item #	Recommendation	Reported on page #
**Title and abstract**	1	(*a*) Indicate the study's design with a commonly used term in the title or the abstract	1
		(*b*) Provide in the abstract an informative and balanced summary of what was done and what was found	2
**Introduction**			
Background/rationale	2	Explain the scientific background and rationale for the investigation being reported	3
Objectives	3	State specific objectives, including any pre-specified hypotheses	3-4
**Methods**			
Study design	4	Present key elements of study design early in the paper	4
Setting	5	Describe the setting, locations, and relevant dates, including periods of recruitment, exposure, follow-up, and data collection	5-6
Participants	6	(*a*) *Cohort study*—Give the eligibility criteria, and the sources and methods of selection of participants. Describe methods of follow-up<br>*Case-control study*—Give the eligibility criteria, and the sources and methods of case ascertainment and control selection. Give the rationale for the choice of cases and controls<br>*Cross-sectional study*—Give the eligibility criteria, and the sources and methods of selection of participants	5-6
		(*b*) *Cohort study*—For matched studies, give matching criteria and number of exposed and unexposed<br>*Case-control study*—For matched studies, give matching criteria and the number of controls per case	
Variables	7	Clearly define all outcomes, exposures, predictors, potential confounders, and effect modifiers. Give diagnostic criteria, if applicable	5-6
Data sources/ measurement	8*	For each variable of interest, give sources of data and details of methods of assessment (measurement). Describe comparability of assessment methods if there is more than one group	*5-6*
Bias	9	Describe any efforts to address potential sources of bias	5-6
Study size	10	Explain how the study size was arrived at	5-6
Quantitative variables	11	Explain how quantitative variables were handled in the analyses. If applicable, describe which groupings were chosen and why	Not applicable
Statistical methods	12	(*a*) Describe all statistical methods, including those used to control for confounding	4-6
		(*b*) Describe any methods used to examine subgroups and interactions	Not applicable
		(*c*) Explain how missing data were addressed	Not applicable
		(*d*) *Cohort study*—If applicable, explain how loss to follow-up was addressed<br>*Case-control study*—If applicable, explain how matching of cases and controls was addressed	Not applicable

For peer review only

		Cross-sectional study—If applicable, describe analytical methods taking account of sampling strategy	
		(*e*) Describe any sensitivity analyses	6
**Results**			
Participants	13*	(a) Report numbers of individuals at each stage of study—eg numbers potentially eligible, examined for eligibility, confirmed eligible, included in the study, completing follow-up, and analysed	Not applicable
		(b) Give reasons for non-participation at each stage	Not applicable
		(c) Consider use of a flow diagram	Not applicable
Descriptive data	14*	(a) Give characteristics of study participants (eg demographic, clinical, social) and information on exposures and potential confounders	7-13
		(b) Indicate number of participants with missing data for each variable of interest	Not applicable
		(c) *Cohort study*—Summarise follow-up time (eg, average and total amount)	Not applicable
Outcome data	15*	*Cohort study*—Report numbers of outcome events or summary measures over time	Not applicable
		Case-control study—Report numbers in each exposure category, or summary measures of exposure	Not applicable
		Cross-sectional study—Report numbers of outcome events or summary measures	7-13
Main results	16	(*a*) Give unadjusted estimates and, if applicable, confounder-adjusted estimates and their precision (eg, 95% confidence interval). Make clear which confounders were adjusted for and why they were included	7-13
		(*b*) Report category boundaries when continuous variables were categorized	Not applicable
		(*c*) If relevant, consider translating estimates of relative risk into absolute risk for a meaningful time period	Not applicable
Other analyses	17	Report other analyses done—eg analyses of subgroups and interactions, and sensitivity analyses	7-13
**Discussion**			
Key results	18	Summarise key results with reference to study objectives	13-15
Limitations	19	Discuss limitations of the study, taking into account sources of potential bias or imprecision. Discuss both direction and magnitude of any potential bias	13-15
Interpretation	20	Give a cautious overall interpretation of results considering objectives, limitations, multiplicity of analyses, results from similar studies, and other relevant evidence	13-15
Generalisability	21	Discuss the generalisability (external validity) of the study results	14
**Other information**			
Funding	22	Give the source of funding and the role of the funders for the present study and, if applicable, for the original study on which the present article is based	16

*Give information separately for cases and controls in case-control studies and, if applicable, for exposed and unexposed groups in cohort and cross-sectional studies.
Note: An Explanation and Elaboration article discusses each checklist item and gives methodological background and published examples of transparent reporting. The STROBE checklist is best used in conjunction with this article (freely available on the Web sites of PLoS Medicine at http://www.plosmedicine.org/, Annals of Internal Medicine at http://www.annals.org/, and Epidemiology at http://www.epidem.com/). Information on the STROBE Initiative is available at www.strobe-statement.org.

For peer review only

BMJ Open

Sensitivity analysis for mistakenly adjusting for mediators in estimating total effect in observational studies

Journal:	*BMJ Open*
Manuscript ID	bmjopen-2016-015640.R3
Article Type:	Research
Date Submitted by the Author:	04-Jul-2017
Complete List of Authors:	Wang, Tingting; Shandong University, Department of Epidemiology and Biostatistics<br>Li, Hongkai; Shandong University, Department of Epidemiology and Biostatistics<br>Su, Ping; Shandong University, Department of Epidemiology and Biostatistics<br>Yu, Yuanyuan; Shandong University, Department of Epidemiology and Biostatistics<br>Sun, Xiaoru; Shandong University, Department of Epidemiology and Biostatistics<br>Liu, Yi; Shandong University, Department of Epidemiology and Biostatistics<br>Yuan, Zhongshang; Shandong University, Department of Epidemiology and Biostatistics<br>Xue, Fuzhong; Shandong University, Department of Epidemiology and Biostatistics
**Primary Subject Heading**:	Epidemiology
Secondary Subject Heading:	Public health
Keywords:	EPIDEMIOLOGY, PUBLIC HEALTH, STATISTICS & RESEARCH METHODS

SCHOLARONE™
Manuscripts

Sensitivity analysis for mistakenly adjusting for mediators in estimating total effect in observational studies

Tingting Wang†, Hongkai Li†, Ping Su, Yuanyuan Yu, Xiaoru Sun, Yi Liu, Zhongshang Yuan, Fuzhong Xue*

Department of Biostatistics, School of Public Health, Shandong University, Jinan City, Shandong Province, P.R.China

Email addresses:

TTW: wangtingting161780@163.com

HKL: lihongkaiyouxiang@163.com

PS: sdsuping@163.com

YYY: yu_yy_1993@163.com

XRS: sunny150139@126.com

YL: liuyi238@sdu.edu.cn

ZSY: yuanzhongshang@sdu.edu.cn

*Corresponding Author

xuefzh@sdu.edu.cn (FZX)

Word Count: 3726

Abstract

Objectives: In observational studies, epidemiologists often attempt to estimate the total effect of an exposure on an outcome of interest. However, when the underlying diagram is unknown and only limited knowledge is available, dissecting bias performances is essential to estimating the total effect of an exposure on an outcome when mistakenly adjusting for mediators under logistic regression. Through simulation, we focus on six causal diagrams concerning different roles of mediators. Sensitivity analysis was conducted to assess the bias performances of varying across exposure-mediator effects and mediator-outcome effects when adjusting for the mediator.

Setting: Based on the causal relationships in the real world, we compare the biases of varying across the effects of exposure-mediator with those of varying across the effects of mediator-outcome when adjusting for the mediator. The magnitude of the

For peer review only

bias was defined by the difference between the estimated effect (using logistic regression) and the total effect of the exposure on the outcome.

Results: In four scenarios (a single mediator, two series mediators, two independent parallel mediators or two correlated parallel mediators), the biases of varying across the effects of exposure-mediator were greater than those of varying across the effects of mediator-outcome when adjusting for the mediator. In contrast, in two other scenarios (a single mediator or two independent parallel mediators in the presence of unobserved confounders), the biases of varying across the effects of exposure-mediator were less than those of varying across the effects of mediator-outcome when adjusting for the mediator.

Conclusions: The biases were more sensitive to the variation of effects of exposure-mediator than the effects of mediator-outcome when adjusting for the mediator in the absence of unobserved confounders, while the biases were more sensitive to the variation of effects of mediator-outcome than those of exposure-mediator in the presence of an unobserved confounder.

Strengths and limitations of this study

1) For six different causal diagrams, we compared biases of distinct adjustment strategies with and without adjusting for mediators by conducting simulation studies.

2) Sensitivity analysis was conducted to assess the performances of varying across the effects of exposure-mediator and mediator-outcome.

3) The simulation schemes and parameters were conducted mainly based on real observational studies.

4) The combination of theoretical derivation and simulation studies make the results more credible.

For peer review only - http://bmjopen.bmj.com/site/about/guidelines.xhtml

For peer review only

5) The limitation of these simulation studies was that they operated under the framework of logistic regression and therefore focused on only binary variables.

Introduction

Estimating the total effect of the exposure (E) on the outcome (D) is a great challenge in epidemiology studies because confounders are commonly confused with mediators.[1-3] If confounders and mediators are misclassified, the ability to control confounders in the estimation of the total effect of the exposure on the outcome is hampered. In fact, various strategies are used to eliminate confounding bias in observational studies. The conventional approaches include multivariate regression, stratification, standardization and inverse-probability weighting.[4-5] Furthermore, causal diagrams provide a formal conceptual framework for identifying and selecting confounders,[6-7] so that analysis can avoid falling into analytic pitfalls.[8] In practice, even the underlying causal diagrams and the role of covariates (mediator, confounder, collider and instrumental variable) are not completely understood, as investigators usually adjust for the covariates that are associated with the outcome and exposure.[9-12] Therefore, our paper focuses on the biases of varying across the effects of exposure-mediator ($E \rightarrow M$) and mediator-outcome ($M \rightarrow D$) when mistakenly adjusting for mediators under the logistic regression model.

Several causal inference studies have made considerable contributions to mediation analysis by providing definitions for direct and indirect effects that allow for the decomposition of a total effect into a direct and an indirect effect.[13-21] Arbitrarily adjusting for a mediator would generally bias the estimate of the total effect of the exposure on the outcome.[8,22-23] Practically, it can mistakenly identify a non-confounding risk factor as a confounder. In the perspective of causal diagrams,

For peer review only - http://bmjopen.bmj.com/site/about/guidelines.xhtml

little attention has been paid to the biases when adjusting for mediators under the logistic regression model in estimating the total effect of *E* on *D*. Hence, we focused on the sensitivity analysis technique to assess the biases of varying across the effects of *E*→*M* and *M*→*D* when adjusting for the mediator.

In this paper, six typical causal diagrams corresponding to causal correlation are given in Figure 1: a single mediator (Figure 1a); two series mediators (Figure 1b); two independent parallel mediators (Figure 1c); two correlated parallel mediators (Figure 1d); a single mediator with an unobserved confounder (Figure 1e); and two parallel mediators with an unobserved confounder (Figure 1f). The paper aims to explore the sensitivity of biases to the variation of the effects of *E*→*D* and *M*→*D* when adjusting for the mediator. Hence, both theoretical proofs and quantitative simulations were performed to dissect the bias of varying across the effect of *E*→*M* and varying across the effect of *M*→*D* when adjusting for mediators under the logistic model.

Methods

A directed acyclic graph (*DAG*) is composed of variables (nodes) and arrows (directed edges) between nodes such that the graph is acyclic. The causal diagrams are formalized as directed acyclic graphs (*DAG*s), providing investigators with powerful tools for bias assessment.[24] It provides a device for deducing the statistical associations implied by causal relations. Furthermore, given a set of observed statistical associations, a researcher knowledgeable about causal diagrams theory can systematically characterize all causal structures compatible with the observations.[25-26]

The total effect of the exposure on the outcome can be calculated based on the *do-calculus* and *back-door* criterion proposed by Judea Pearl.[27-28] For exposure *X* and

For peer review only - http://bmjopen.bmj.com/site/about/guidelines.xhtml

outcome Y, a set of variables Z satisfies the backdoor path criterion with respect to (X, Y) if no variable in Z is a descendant of X and Z blocks all back-door paths from X to Y. Then, the effect of X on Y is given by the following formula:

$$P(y \mid do(x)) = \sum_{Z} P(y \mid x, z) P(z)$$

Note that the expression on the right hand side of the equation is simply a standardized mean. The difference $E(Y \mid do(x')) - E(Y \mid do(x''))$ is taken as the definition of "causal effect", where x' and x'' are two distinct realizations of X.[23] The interventional distribution, such as that corresponding to $Y(x)$, namely $P(y|do(x))$, is not necessarily equal to a conditional distribution $P(y \mid x)$. It stands for the probability of $Y = y$ when the exposure X is set to level x. The ignorability assumption $Y(x) \perp X$ states that if we happen to have information on the exposure variable, it does not give us any information about the outcome Y after the intervention $do(x)$ was performed. In addition, it can be shown that if ignorability holds for $Y(x)$ and X (alternatively if there are no back-door paths from X to Y in the corresponding causal DAGs), then $P(y \mid do(x)) = P(y \mid x)$.[29-30]

Let D_e and M_e denote the values of the outcome and mediator that would have been observed had the exposure E been set to level e, respectively. On the odds ratio ($OR^{TE}_{E \to D}$) scale, the total effect ($\beta^{TE}_{E \to D} = \log(OR^{TE}_{E \to D})$), comparing exposure level e with e*, is given as the following: $OR^{TE}_{E \to D} = \dfrac{P(D_e = 1) / \{1 - P(D_e = 1)\}}{P(D_{e*} = 1) / \{1 - P(D_{e*} = 1)\}}$.[20-21] While the effect ($\beta_{ED|M}(m)$) of adjusting for mediator M by the logistic regression model can be given as the following:

For peer review only - http://bmjopen.bmj.com/site/about/guidelines.xhtml

$$\beta_{ED|M}(m) = \text{logit}\left\{P(D=1|e=1,m)\right\} - \text{logit}\left\{P(D=1|e^*=0,m)\right\}$$
$$= \log\left\{\frac{P(D=1|e=1,m)P(D=0|e^*=0,m)}{P(D=0|e=1,m)P(D=1|e^*=0,m)}\right\}$$

where $P(D=1|e,m)$ denotes the probability of $D=1$ when the exposure E and mediator M have been set to level e and m, respectively. Taking Figure 1a as an example, the logistic regression is as follows:

$$\text{logit}\{P(D=1|e,m)\} = \alpha_1 + \beta_0 e + \beta_2 m\,.$$

Therefore, the total effect ($\beta^{TE}_{E\to D}$) of exposure E on outcome D on the scale of logarithm odds ratio was equal to

$$\beta^{TE}_{E\to D} = \log(OR^{TE}_{E\to D})$$
$$= \log\left\{\frac{P(D_e=1)/\{1-P(D_e=1)\}}{P(D_{e^*}=1)/\{1-P(D_{e^*}=1)\}}\right\}$$
$$= \text{logit}\left\{P(D_e=1)\right\} - \text{logit}\left\{P(D_{e^*}=1)\right\}$$
$$= \text{logit}\left\{P(D=1|e=1)\right\} - \text{logit}\left\{P(D=1|e^*=0)\right\}$$
$$= \text{logit}\left\{\sum_m P(D=1|e=1,m)P(m|e=1)\right\} - \text{logit}\left\{\sum_m P(D=1|e^*=0,m)P(m|e^*=0)\right\}$$

The effect estimation ($\hat{\beta}_{ED|M}(m)$) of adjusting for mediator M by the logistic regression model was equal to

$$\hat{\beta}_{ED|M}(m) = \text{logit}\left\{\hat{P}(D=1|e=1,m)\right\} - \text{logit}\left\{\hat{P}(D=1|e^*=0,m)\right\}$$

where $\hat{P}(D=1|e=1,m)$ denotes the probability of $D=1$ when the exposure E and mediator M have been set to level $e=1$ and m, respectively. Additionally, $\hat{P}(D=1|e^*=0,m)$ denotes the probability of $D=1$ when the exposure E and mediator M have been set to level $e^*=0$ and m, respectively. The theoretical results of other causal diagrams in Figure 1 have been shown in the supplementary A.

Note that the bias was defined by taking a difference between effect estimation by adjusting for the mediator using logistic regression and the total effect of exposure E on outcome D i.e., $bias = E[\hat{\beta}_{ED|M}(m)] - \beta^{TE}_{E\to D}$. We dissected the behavior of the biases by varying across the effects of $E\to M$ and $M\to D$ when mistakenly adjusting

For peer review only - http://bmjopen.bmj.com/site/about/guidelines.xhtml

for the mediator under the framework of the logistic regression model.

Simulation

Six scenarios are designed to dissect the sensitivity of bias to the variation of the effects of exposure-mediator and mediator-outcome when adjusting for mediators under the framework of the logistic regression model; these *DAG*s are shown in Figure 1. We made the following assumptions for the simulation: 1) all variables were binary, following a Bernoulli distribution; and 2) the effects from parent nodes to their child node were positive and log-linearly additive. Taking Figure 1a as an example, we randomly generated the exposure following a Bernoulli distribution (i.e. let $P(e=1)=\pi$). Then, we used $P_M = \exp(\alpha_0 + \beta_1 e) / \{1+\exp(\alpha_0 + \beta_1 e)\}$ to calculate the distribution probability of child node *M* from its parent node *E*. Similarly, $P_D = \exp(\alpha_1 + \beta_0 e + \beta_2 m) / \{1+\exp(\alpha_1 + \beta_0 e + \beta_2 m)\}$ generated the distribution probability of *D*, where the parameters α_0 and α_1 denoted the intercept of *M* and *D* respectively, and effect parameters β_0, β_1, β_2 referred to the effects of the parent node on their corresponding child node using a log odds ratio scale.

After generating data, we dissected the behavior of the biases between the effects of $E \rightarrow M$ and $M \rightarrow D$ when mistakenly adjusting for mediators under the logistic regression model. In scenario 1 (Figure 1a), we compared performances by varying across the effects of $E \rightarrow M$ and $M \rightarrow D$. Similarly, in scenario 2 (Figure 1b), the effects of $E \rightarrow M_1$, $M_1 \rightarrow M_2$ and $M_2 \rightarrow D$ were explored. In scenario 3 (Figure 1c), we dissected the effects of $E \rightarrow M_1$ ($E \rightarrow M_2$) and $M_1 \rightarrow D$ ($M_2 \rightarrow D$). The comparison of scenario 4 (Figure 1d) was the same as scenario 3 (Figure 1c). In scenario 5 (Figure 1e), the

For peer review only - http://bmjopen.bmj.com/site/about/guidelines.xhtml

effects of $E \rightarrow M$ and $M \rightarrow D$ were excavated. Scenario 6 (Figure 1f) was identical to the scenario 3. We explored the biases when adjusting for mediators under the logistic regression model and thus identified the sensitivity of biases to the variation of the effects of exposure-mediator and mediator-outcome.

For each of the 6 simulation scenarios, we observed the biases of varying across distinct effects when adjusting for mediators using the logistic regression model with 1000 simulation repetitions. All simulations were conducted using software R from CRAN (http://cran.r-project.org/).

Results

Scenario 1: one single mediator (Figure 1a)

In Figure 1a, *E* has a direct ($E \rightarrow D$) effect and an indirect ($E \rightarrow M \rightarrow D$) effect on *D*. In Figure 2, Figure 2A depicted that the bias of varying across the effect of $E \rightarrow M$ was clearly greater than the bias of varying across the effect of $M \rightarrow D$. That is, the sensitivity of bias to the variation of the effect $E \rightarrow M$ was greater than that of the effect of $M \rightarrow D$ when adjusting for the mediator *M* using the logistic regression model. In particular, if the effect of $E \rightarrow M$ was specified to zero in Figure 2B, *M* would be associated with *D* conditional on *E* and unconditionally independent with *E*, and *M* would become an independent risk factor of the outcome, as adjusting for *M* would obtain a positive "bias". Such bias was a consequence of the non-collapsibility of the odds ratio, and the M-conditional ORs must be farther from 1 than the unconditional ORs.[31-32] In fact, both adjustment and non-adjustment for *M* should yield unbiased causal effect estimates. Certainly, in this case, both the marginal OR and conditional OR obtained from standardization and inverse-probability weighting were equal to the total effect.[33] Moreover, Figure 2A indicated that adjusting for mediator *M* was indeed biased to the total effect of the exposure on the outcome.

For peer review only - http://bmjopen.bmj.com/site/about/guidelines.xhtml

The total effect ($\beta^{TE}_{E\to D}$) of exposure E on outcome D on the log odds ratio scale was equal to

$$\begin{aligned}\beta^{TE}_{E\to D} = \log(OR^{TE}_{E\to D}) &= \log\left\{\frac{P(D_e=1)/\{1-P(D_e=1)\}}{P(D_{e*}=1)/\{1-P(D_{e*}=1)\}}\right\}\\ &= \log\left\{\frac{P(D_e=1)\times\{1-P(D_{e*}=1)\}}{\{1-P(D_e=1)\}\times P(D_{e*}=1)}\right\}\\ &= \log\left\{\frac{P(D=1\mid e=1)\times P(D=0\mid e*=0)}{P(D=0\mid e=1)\times P(D=1\mid e*=0)}\right\}\\ &= \log\left\{\frac{[\sum_m P(D=1\mid e=1,m)P(m\mid e=1)]\times[\sum_m P(D=0\mid e*=0,m)P(m\mid e*=0)]}{[\sum_m P(D=0\mid e=1,m)P(m\mid e=1)]\times[\sum_m P(D=1\mid e*=0,m)P(m\mid e*=0)]}\right\}\end{aligned}$$

The effect ($\beta_{ED|M}(m)$) of adjusting for mediator M by the logistic regression model can be given as follows:

$$\begin{aligned}\beta_{ED|M}(m) &= \operatorname{logit}\{P(D=1\mid e=1,m)\} - \operatorname{logit}\{P(D=1\mid e*=0,m)\}\\ &= \log\left\{\frac{P(D=1\mid e=1,m)\times\{1-P(D=1\mid e*=0,m)\}}{\{1-P(D=1\mid e=1,m)\}\times P(D=1\mid e*=0,m)}\right\}\\ &= \beta_0\end{aligned}$$

β_0 denotes coefficient of E adjusting for M using the logistic regression model. Furthermore, the effect of adjusting for M was equal to the controlled direct effect.[19] Therefore, the bias of adjusting for the mediator using the logistic regression model could be obtained i.e., $bias = \beta_{ED|M}(m) - \beta^{TE}_{E\to D}$. We added signs to the edges of the directed acyclic graph to indicate the presence of a particular positive or negative effect in Figure 3. Therefore, we gained $bias < 0$ under the condition of $\beta_1 * \beta_2 > 0$ (the effect $E\to M$ β_1 and the effect $M\to D$ β_2), indicating that the total effect of E on D was biased when adjusting for M using the logistic regression model in Figure 3a, Figure 3b, Figure 3e & Figure 3f. In addition, the bias was less than zero when the effect $E\to M$ (β_1) and the effect $M\to D$ (β_2) shared same signs. (i.e., both the effects

For peer review only

For peer review only - http://bmjopen.bmj.com/site/about/guidelines.xhtml

$E \to M$ ($\beta_1 > 0$) and $M \to D$ ($\beta_2 > 0$) were a positive sign or both the effects $E \to M$ ($\beta_1 < 0$) and $M \to D$($\beta_2 < 0$) were a negative sign). Furthermore, we obtained $bias > 0$, if $\beta_1 * \beta_2 < 0$, suggesting that the total effect of E on D was biased when adjusting for M in Figure 3c, Figure 3d, Figure 3g & Figure 3h. In addition, the bias was greater than zero when the signs of the effects $E \to M$ (β_1) and $M \to D$ (β_2) were the opposite. The results illustrated that the bias was less than zero in the case in which the effects of exposure-mediator and mediator-outcome shared the same sign; the bias was greater than zero under the circumstance in which the effects of exposure-mediator and mediator-outcome had opposite signs. We also illustrated the case of Figure 3c with the effects $E \to M$ and $E \to D$ as greater than zero, and the effect $M \to D$ as less than zero in supplementary B. More details of theoretical derivation can be found in Appendix.

Scenario 2: two series mediators (Figure 1b)

Figure 1b is a depiction through two series mediators, decomposing total effects into direct effect ($E \to D$) and indirect effect ($E \to M_1 \to M_2 \to D$). The bias of varying across the effect of $E \to M_1$ was greater than that of varying across the effect of $M_2 \to D$ under adjustment for M_1, M_2 and M_1 M_2 together in Figure 4, respectively. In this situation, the correlation of series mediators was strong enough to prevent M_2 from becoming an independent cause of the outcome.

Scenario 3: two independent parallel mediators (Figure 1c)

Figure 1c shows that the exposure E independently causes M_1 and M_2 and indirectly influences the outcome D through M_1 and M_2, forming three causal paths $E \to D$,

For peer review only - http://bmjopen.bmj.com/site/about/guidelines.xhtml

$E \to M_1 \to D$ and $E \to M_2 \to D$. For Figure 5, the results indicated that the bias of varying across the effect of $E \to M_1$ was considerably greater than that of varying across the effect of $M_1 \to D$ under adjustment for M_1 in Figure 5A. However, the bias of varying across the effect of $E \to M_2$ was nearly equal to that of varying across the effect of $M_2 \to D$ under the identical model of adjustment for M_1 in Figure 5A. Then, a result similar to the one above can be obtained in Figure 5B. In addition, Figure 5C indicated that biases of varying across the effects of $E \to M_1$ and $E \to M_2$ were obviously greater than those of varying across the effects of $M_1 \to D$ and $M_2 \to D$ while simultaneously adjusting for M_1 and M_2.

Scenario 4: two correlated parallel mediators (Figure 1d)

In Figure 1d, there exist five paths from E to D: $E \to D$, $E \to M_1 \to D$, $E \to M_2 \to D$, $E \to M_1 \leftarrow M_2 \to D$ and $E \to M_2 \to M_1 \to D$. In particular, the path $E \to M_1 \leftarrow M_2 \to D$ is a blocked path, due to M_1 being a collider node. In Figure 6, Figure 6A indicated that the bias of varying across the effect of $E \to M_1$ was clearly greater than that of varying across the effect of $M_1 \to D$ under adjustment for M_1. However, the bias of varying across the effect of $E \to M_2$ was almost equal to that of varying across the effect of $M_2 \to D$ under the identical adjustment model. Similarly, an analogous result of the behavior of the biases is shown in Figure 6B. In addition, the biases of varying across the effects of $E \to M_1$ and $E \to M_2$ were greater than those of varying across the effects of $M_1 \to D$ and $M_2 \to D$ when adjusting for M_1 and M_2 in Figure 6C. Simultaneously, the bias was more sensitive to the variation of the effect of $E \to M_2$ than the effect of $E \to M_1$ under adjustment for M_1 and M_2, while adjusting for the collider node M_1 would partially open the path $E \to M_1 \leftarrow M_2 \to D$.

Scenario 5: a single mediator with an unobserved confounder (Figure 1e)

For peer review only - http://bmjopen.bmj.com/site/about/guidelines.xhtml

Figure 1e provides a causal diagram representing the relationship among exposure E, outcome D, mediator M and unobserved confounder U. It revealed that the bias of varying across the effect of $E \rightarrow M$ was lower than that of varying across the effect of $M \rightarrow D$. An unobserved confounder distorts the association between the exposure and outcome ($E \leftarrow U \rightarrow D$) in Figure 7.

Scenario 6: two parallel mediators with an unobserved confounder (Figure 1f)

As described above, Figure 1f is a depiction of two parallel mediators M_1 and M_2 with an unobserved confounder U. For Figure 8, the bias of varying across the effect of $E \rightarrow M_1$ was clearly less than that of varying across the effect of $M_1 \rightarrow D$ under the adjustment for M_1 in Figure 8A. However, the bias of varying across the effect of $E \rightarrow M_2$ was greater than that of varying across the effect of $M_2 \rightarrow D$ under the identical model adjusting for M_1. A similar result can also be obtained in Figure 8B. In addition, biases of varying across the effects of $E \rightarrow M_1$ and t $E \rightarrow M_2$ were distinctly less than those of varying across the effects of $M_1 \rightarrow D$ and $M_2 \rightarrow D$ under the common model of adjusting for M_1 and M_2 in Figure 8C.

Application

In this analysis, we evaluated two statistical models (unadjusted and M-adjusted) to assess the effect of diabetes on cardiovascular diseases under scenario 1. Information from 22,900 individuals were collected from the Health Management Center of Shandong Provincial Hospital (HMCSPH). All individuals were Urban Han Chinese and more than 20 years of age and they underwent a physical examination in 2013. Many studies focused on the associations between diabetes and metabolic

For peer review only - http://bmjopen.bmj.com/site/about/guidelines.xhtml

syndrome,[34] and between metabolic syndrome and cardiovascular disease.[35]

The exposure indicator E takes a value of 1 if individuals suffer from diabetes and takes a value of zero otherwise. The outcome D (cardiovascular diseases) takes a value of 1 if individuals are diagnosed with cardiovascular diseases and takes a value of 0 otherwise. The mediator M (metabolic syndrome) takes a value the value of 1 if individuals diagnosed with metabolic syndrome and takes a value of 0 otherwise. After adjusting for age and gender, using the logistic regression model obtained the total effect of diabetes E on cardiovascular diseases D equal to $\beta = 0.598$ (95% confidence interval (CI), 0.307~0.877). Then, the effect of adjusting for metabolic syndrome M was equal to $\beta_M = 0.429$ (95% confidence interval (CI), 0.113~0.736). Therefore, the bias was $\beta_M - \beta = -0.169 < 0$, suggesting that the effect of E on D was underestimated when adjusting for the mediator M. This bias can have negative implications on the interpretation of the effects of diabetes on cardiovascular diseases. The adjustment for the mediator produced biased estimates, and adjustment was thus inappropriate and should have been avoided. A specific example was the adjustment for time-varying confounders that are also mediators using methods including standardization, inverse-probability weighting, and G-estimation.[36] That is, investigators should remember to consider biological and clinical information when specifying a statistical model.

Discussion

In the paper, we dissected the sensitivity of bias to the variation of the effects of exposure-mediator and mediator-outcome when adjusting for mediators under the framework of the logistic regression model. In four scenarios (a single mediator in

For peer review only - http://bmjopen.bmj.com/site/about/guidelines.xhtml

Figure 1a of scenario 1, two series mediators in Figure 1b of scenario 2, two independent parallel mediators in Figure 1c of scenario 3 or two correlated parallel mediators in Figure 1d of scenario 4), the bias of varying across the effect of exposure-mediator was greater than that of varying across the effect of mediator-outcome when adjusting for the mediator (Figure 2, Figure 4, Figure 5 & Figure 6). However, in two other scenarios (a single mediator or two independent parallel mediators in the presence of unobserved confounders in Figure 1e of scenario 5 & Figure 1f of scenario 6), the biases were more sensitive to the variation of the effect of mediator-outcome than the effect of exposure-mediator when adjusting for the mediator (Figure 7 & Figure 8).

Conditioning on a mediator is of concern in all areas of epidemiologic studies,[13,19,37] it indeed lead to bias in estimating the total effect of the exposure on the outcome.[8,22-23] Mediators and confounders are indistinguishable in terms of statistical association and conceptual grounds.[3] Most of the studies focus on the mediation effect analysis such as the calculation of direct effect and indirect effect.[20-21,38-41] Recently, some authors have used causal diagrams to describe how to appropriately handle matching variables. In addition, they have proven that matching on mediator M renders M and D independent (by design) in the matched study. Matching on variables that are affected by the exposure and the outcome, i.e., mediators between the exposure and the outcome, would ordinary produce irremediable bias. Furthermore, matching on mediator M blocks the causal path $E \rightarrow M \rightarrow D$ and thus produces unfaithfulness in estimating the total effect E on D.[31,42] Little effort has been made to learn the performances of biases when adjusting for a mediator in estimating the total effect of an exposure on an outcome. Our study results revealed that the biases were more sensitive to the variation of the effects of exposure-mediator than effects of

For peer review only - http://bmjopen.bmj.com/site/about/guidelines.xhtml

mediator-outcome when adjusting for the mediator in the absence of the unobserved confounder in causal diagrams (Figure 1a, Figure 1b, Figure 1c & Figure 1d). Nevertheless, for causal diagrams (Figure 1e & Figure 1f), the biases were more sensitive to the variation of effects of mediator-outcome than the effects of exposure-mediator when adjusting for a mediator in the presence of the unobserved confounder. Therefore, the biases of varying across different effects depended on the causal diagrams framework and whether an unobserved confounder existed.

The causal diagrams depicted in Figure 1 are indeed very simplistic and concise, as they exclude the confounding factors of *E* and *M* as well as *M* and *D*. In practical applications, there exist some confounders in each pair of relationships among *E*, *M*, and *D*. In addition, our simulation study was not comprehensive enough to evaluate the bias performances when adjusting for the mediator under logistic regression because it considered only binary variables, certain scenarios of effect size and common types of models. In medical research, regression modeling is commonly used to adjust for covariates associated with both the outcome and exposure. In this paper, the biases are defined by the difference between M-adjusted and unadjusted ORs, some of which is attributable to the non-collapsibility of the OR. In the field of causal inference, standardization and inverse-probability weighting may obtain a different bias from that of regression modeling, and they may be better alternatives to calculate bias[4-5]. Therefore, in future research, the methods of standardization and inverse-probability weighting could be used to calculate the biases of this paper definition. Future research should further reinforce the mechanisms and conceptual frameworks of confounders and mediators from causal diagrams to avoid falling into

For peer review only - http://bmjopen.bmj.com/site/about/guidelines.xhtml

analytic pitfalls.

Conclusion

In conclusion, the sensitivity of biases to the variation of the effects of exposure-mediator and mediator-outcome were related to whether there was an unobserved confounder in causal diagrams. The biases were more sensitive to the variation of the effects of exposure-mediator than the effects of mediator-outcome when adjusting for the mediator in the absence of unobserved confounders, while the biases were more sensitive to the variation of the effects of mediator-outcome than the effects of exposure-mediator in the presence of unobserved confounders.

Statements

Acknowledgments

We would like to thank the anonymous reviewers and academic editors for providing us with constructive comments and suggestions and also wish to acknowledge our colleagues for their invaluable work.

Authors' contributions

TTW and HKL jointly conceived the idea behind the article and designed the study. TTW helped conduct the literature review, performed the simulation and prepared the draft of the manuscript. PS, YYY, XRS, YL and ZSY participated in the design of the study and the revision of the manuscript. FZX advised on critical revision of the manuscript for important intellectual content. All authors read and approved the final manuscript.

Funding

This work was supported by grants from the National Natural Science Foundation of China (grant number 81573259)

Competing interests

The authors declare that they have no competing interests.

For peer review only - http://bmjopen.bmj.com/site/about/guidelines.xhtml

Ethics approval and materials

Ethics Committee of the School of Public Health (20140322), Shandong University.

Written informed consent was obtained from all participants.

Provenance and peer review

Not commissioned; externally peer reviewed.

Data sharing statement

No additional data are available.

Open Access

This is an Open Access article distributed in accordance with the Creative Commons Attribution Non Commercial (CC BY-NC 4.0) license, which permits others to distribute, remix, adapt, build upon this work noncommercially, and license their derivative works on different terms, provided the original work is properly cited and the use is non-commercial. See: http:// creativecommons.org/licenses/by-nc/4.0/

References

1 Bulterys M, Morgenstern H. Confounding or intermediate effect? An appraisal of iatrogenic bias in perinatal AIDS research. *Paediatr Perinat Epidemiol* 1993;7:387–394.

2 Herna´n MA, Hernandez-Diaz S, Werler MM, et al. Causal knowledge as a prerequisite for confounding evaluation: an application to birth defects epidemiology. *Am J Epidemiol* 2002;155:176–184.

3 MacKinnon DP, Krull JL, and Lockwood CM. Equivalence of the mediation, confounding and suppression effect. *Prevention Science* 2000;1:173–181.

4 Mansournia MA, Altman DG. Inverse probability weighting[J]. *Bmj*, 2016, 352:i189.

5 Gharibzadeh S, Mohammad K, Rahimiforoushani A, Amouzegar A, Mansournia MA. Standardization as a tool for causal inference in medical research. *Arch Iran Med*. 2016;19:666-70.

6 Howards PP, Schisterman EF, Poole C, et al. "Toward a clearer definition of confounding" revisited with directed acyclic graphs, *Am J Epidemiol* 2012;176:506–511.

7 Williamson EJ, Aitken Z, Lawrie J, et al. Introduction to causal diagrams for confounder selection. *Respirology*. 2014;19:303-311.

8 Wil cox AJ, Weinberg CR, Basso O. On the pitfalls of adjusting for gestational age at birth. *Am J Epidemiol*. 2011;174:1062–1068.

For peer review only - http://bmjopen.bmj.com/site/about/guidelines.xhtml

For peer review only

9 Psaty BM, Koepsell TD, Lin D, et al. Assessment and control for confounding by indication in observational studies. *J Am Geriatr Soc* 1999; 47:749-54.

10 Tilaki K H. Methodological issues of confounding in analytical epidemiology studies. *Caspian J intern Med* 2012;3:488-495.

11 Kamangar F MD. Confounding variable in epidemiology studies: Basics and beyond. *Arch Tran Med*. 2012;15:508-516.

12 Gaskins AJ, Schisterman EF. The effect of lipid adjustment on the analysis of environmental contaminants and the outcome of human health risks. *Methods Mol Biol* 2009;580:371-381.

13 Robins JM, Greenland S. Identifiability and exchangeability for direct and indirect effects. *Epidemiology* 1992;3:143–155.

14 Pearl J. Direct and indirect effects. Seventeenth conference of uncertainy in artificial intelligence, 2001. San Francisco, CA: Morgan Kaufmann, 2001.

15 Huang B, Sivaganesan S, Succop P, et al. Statistical assessment of mediational effects for logistic mediational models. *Statist. Med* 2004;23:2713-2728.

16 Valeri L, Coull B A. Estimating causal contrasts involving intermediate variables in the presence of selection bias [J]. *Statistics in Medicine*, 2016, 35(26): 4779-4793.

17 MacKinnon D P, Pirlott A G. Statistical approaches for enhancing causal interpretation of the M to Y relation in mediation analysis [J]. *Personality and Social Psychology Review*, 2015, 19(1): 30-43.

18 Imai K, Keele L, Tingley D. A general approach to causal mediation analysis. *Psychol Methods*. 2010;15(4):309–334.

19 VanderWeele TJ. Bias formulas for sensitivity analysis for direct and indirect effects. *Epidemiology*. 2010;21(4):540–551.

20 VanderWeele TJ, Vansteelandt S. Odds ratios for mediation analysis for a dichotomous outcome. *Am J Epidemiol*. 2010;172(12):1339–1348.

21 Valeri L, VanderWeele TJ. Mediation analysis allowing for exposure-mediator interactions and causal interpretation: theoretical assumptions and implementation with SAS and SPSS macros [J]. *Psychological methods*, 2013, 18(2): 137.

22 Robins J. The control of confounding by intermediate variable. *Statistics in Medicine*. 1989;8:679-701.

23 Schistermana EF, Coleb SR, Platt RW. Overadjustment Bias and Unnecessary Adjustment in Epidemiologic Studies. *Epidemiology*. 2009;20:488–495.

24 Pearl J. Causal diagrams for empirical research. *Biometrika*. 1995;82:669-688.

25 Pearl J. Causality: models, reasoning, and inference. *Econometric Theory*. 2003;19: 675-685.

26 Joffe M, Gambhir M, Chadeau-Hyam M, et al. Causal diagrams in systems epidemiology. *Emerg Themes Epidemiol*. 2012; 9:1-18.

27 Pearl, J. Causality: models, reasoning, and inference. Cambridge: Cambridge University Press; 2000.

28 Pearl J. Causal inference in statistics: an overview. *Statistics Surveys*. 2009; 3:96–146.

For peer review only - http://bmjopen.bmj.com/site/about/guidelines.xhtml

29 Rubin DB. Should observational studies be designed to allow lack of balance in covariate distributions across treatment groups? *Statistics in Medicine*. 2009;28:1420-1423.
30 Shpitser I. Counterfactual graphical models for longitudinal mediation analysis with unobserved confounding. *Cognitive Science* 2013;37:1011-1035.
31 Mansournia MA, Hernán MA, Greenland S. Matched designs and causal diagrams. *Int J Epidemiol*. 2013;42:860-869.
32 Greenland S, Robins JM, Pearl J. Confounding and collapsibility in causal inference. *Stat Sci* 1999;14:29–46.
33 Mansournia MA, Greenland S. The relation of collapsibility and confounding to faithfulness and stability. *Epidemiology*. 2015;26:466-72.
34 Clausen TD, Mathiesen ER, Hansen T, et al. Overweight and the metabolic syndrome in adult offspring of women with diet-treated gestational diabetes mellitus or type 1 diabetes.[J]. *J Clin Endocrinol Metab*, 2013, 94:2464-70.
35 Cheng JY, Wong VW, Tse YK, et al. Metabolic syndrome increases cardiovascular events but not hepatic events and death in patients with chronic hepatitis B[J]. *Hepatology*, 2016, 64:1507.
36 Mansournia MA, Danaei G, Forouzanfar MH, Mahmoudi M, Jamali M, Mansournia N, Mohammad K. Effect of physical activity on functional performance and knee pain in patients with osteoarthritis: analysis with marginal structural models. *Epidemiology*. 2012;23:631-40.
37 VanderWeele TJ, Mumford SL, Schisterman EF. Conditioning on intermediates in perinatal epidemiology. *Epidemiology*. 2012;23(1):1–9.
38 Lange T, Rasmussen M, Thygesen LC. Assessing natural direct and indirect effects through multiple pathways. *American Journal of Epidemiology*. 2013;179, 513–518.
39 Shpitser, Ilya and VanderWeele, Tyler J. "A complete graphical criterion for the adjustment formula in mediation analysis," *The International Journal of Biostatistics*.2011;7,1-24.
40 Albert J M, Wang W. Sensitivity analyses for parametric causal mediation effect estimation [J]. *Biostatistics*, 2015, 16(2): 339-351.
41 Wang W, Zhang B. Assessing natural direct and indirect effects for a continuous exposure and a dichotomous outcome[J]. *Journal of Statistical Theory and Practice*, 2016, 10(3): 574-587.
42 Li H, Yuan Z, Su P, et al. A simulation study on matched case-control designs in the perspective of causal diagrams[J]. *BMC Med Res Methodol*, 2016,16,102 .

Figure 1： Six causal diagrams were designed for estimating the causal effect of *E* on *D*. a) a single mediator *M*; b) two series mediators M_1 and M_2; c) two independent parallel mediators M_1 and M_2; d) two correlated parallel mediators M_1 and M_2; e) a single mediator with an unobserved confounder *U*; f) two independent parallel mediators M_1 and M_2 with an unobserved confounder *U*.

Figure 2： The biases with the effects of $E \rightarrow M$ (red) and $M \rightarrow D$ (blue) increasing,

For peer review only - http://bmjopen.bmj.com/site/about/guidelines.xhtml

respectively. Comparison of the bias of different effects in adjustment mediator. The OR of target effect (e.g. $E \rightarrow M$) from 1 to 10 given other effects fixed ln2 in Figure 2A. The OR of the effect of $M \rightarrow D$ from 1 to 10 with the effect of $E \rightarrow M$ being equal to zero in Figure 2B (Color figure online).

Figure 3: Illustrating the use of positive and negative signs on edges $E \rightarrow M$, $M \rightarrow D$ and $E \rightarrow D$.

Figure 4: The biases with the effects of $E \rightarrow M_1$ (red), $M_1 \rightarrow M_2$ (blue) and $M_2 \rightarrow D$ (black) increasing, respectively. Comparison of the bias of different effects in three adjustment models: A) adjustment for M_1, B) adjustment for M_2 and C) adjustment for M_1 and M_2. The OR of target effect (e.g. $E \rightarrow M_1$) from 1 to 10 given the effect of $M_1 \rightarrow M_2$ fixed ln8 and other effects fixed ln2 in Figure 4 (Color figure online).

Figure 5: The biases with the effects of $E \rightarrow M_1$ (red), $E \rightarrow M_2$ (blue), $M_1 \rightarrow D$ (black) and $M_2 \rightarrow D$ (green) increasing, respectively. Comparison of the bias of different effects in three adjustment models: A) adjustment for M_1, B) adjustment for M_2 and C) adjustment for M_1 and M_2. The OR of target effects (e.g. $E \rightarrow M_1$) from 1 to 10 given other edges effects fixed ln2 in Figure 5 (Color figure online).

Figure 6: The biases with the effects of $E \rightarrow M_1$ (red), $E \rightarrow M_2$ (blue), $M_1 \rightarrow D$ (black), $M_2 \rightarrow D$ (green) and the effect of $M_2 \rightarrow M_1$ (purple) increasing, respectively. Comparison of the bias of different effects in three adjustment models: A) adjustment for M_1, B) adjustment for M_2 and C) adjustment for M_1 and M_2. The OR of target effects (e.g. $E \rightarrow M_1$) from 1 to 10 given other effects fixed ln2 in Figure 6 (Color figure online).

Figure 7: The biases with the effects of $E \rightarrow M$ (red) and $M \rightarrow D$ (blue) respectively. Comparison of the bias of different effects in adjustment mediator M. The OR of target effects (e.g. $E \rightarrow M$) from 1 to 10 given the effects of causal edges fixed ln2 and the effect of confounder edges fixed ln5 in Figure ln8 (Color figure online).

Figure 8: The biases with the effects of $E \rightarrow M_1$ (red), $E \rightarrow M_2$ (blue), $M_1 \rightarrow D$ (black) and $M_2 \rightarrow D$ (green) respectively. Comparison of the bias of different effects in three adjustment models: A) adjustment for M_1, B) adjustment for M_2, and C) adjustment for M_1 and M_2. The OR of target effects (e.g. $E \rightarrow M_1$) from 1 to 10 given the effects of causal edges fixed ln2 and the effect of confounder edges fixed ln5 in Figure 8 (Color figure online).

For peer review only - http://bmjopen.bmj.com/site/about/guidelines.xhtml

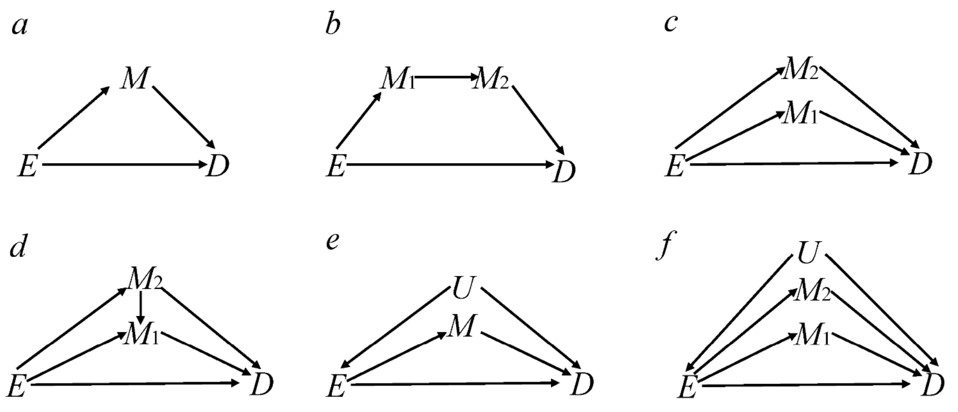

Figure 1: Six causal diagrams were designed for estimating the causal effect of E on D.

252x110mm (300 x 300 DPI)

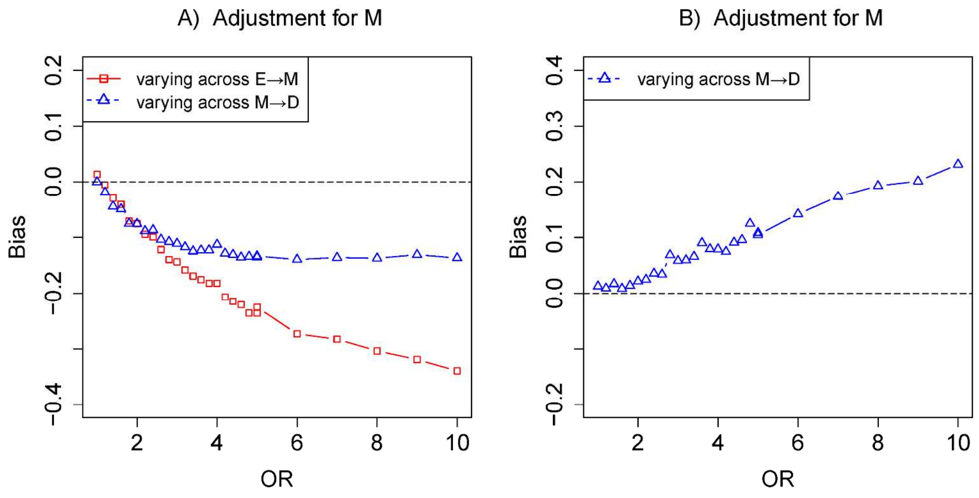

Figure 2 : The biases with the effects E→M (red) and M→D (blue) increasing, respectively.

281x148mm (300 x 300 DPI)

For peer review only

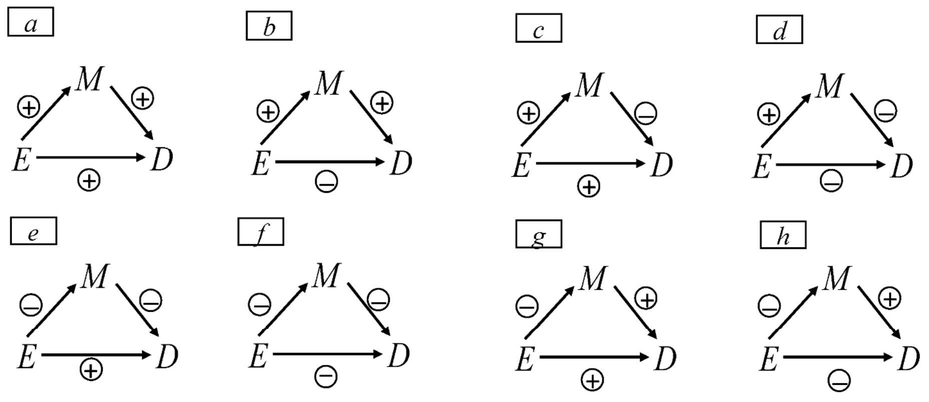

Figure 3: Illustrating the use of positive and negative signs on edges E→M, M→D and E→D.

237x106mm (300 x 300 DPI)

For peer review only

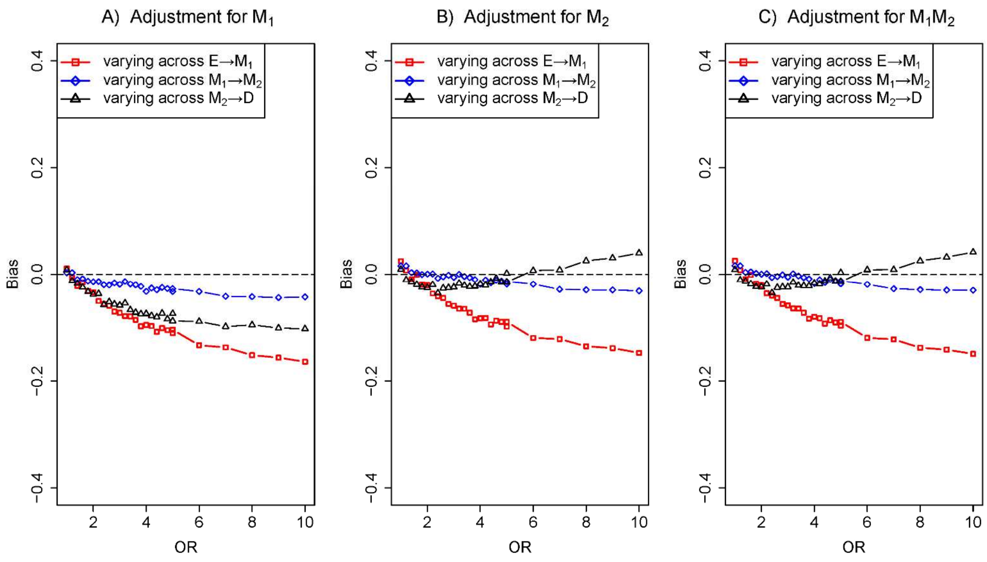

Figure 4: The biases with the effects E→M_1 (red), M_1→M_2 (blue) and M_2→D (black) increasing, respectively.

270x155mm (300 x 300 DPI)

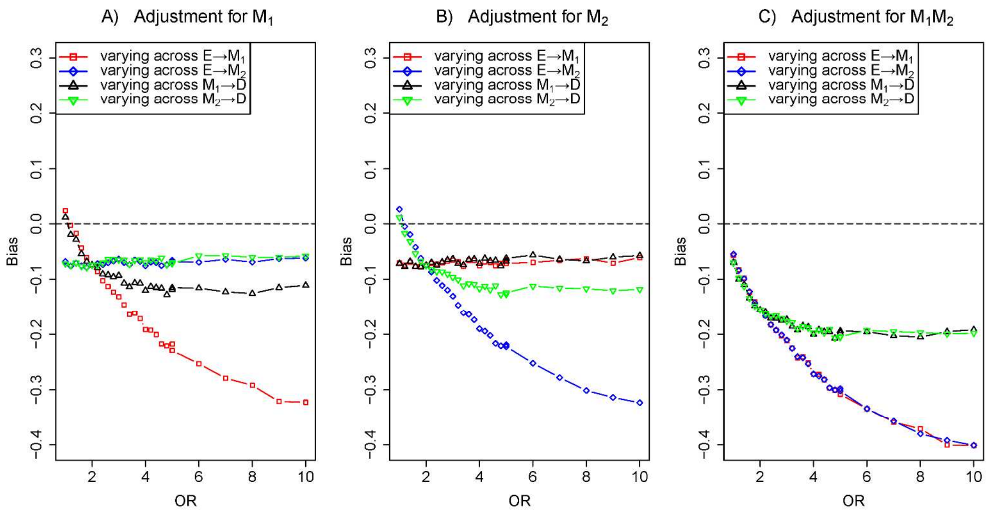

Figure 5 : The biases with the effects E→M_1 (red), E→M_2 (blue), M_1→D (black) and M_2→D (green) increasing, respectively.

279x147mm (300 x 300 DPI)

review only

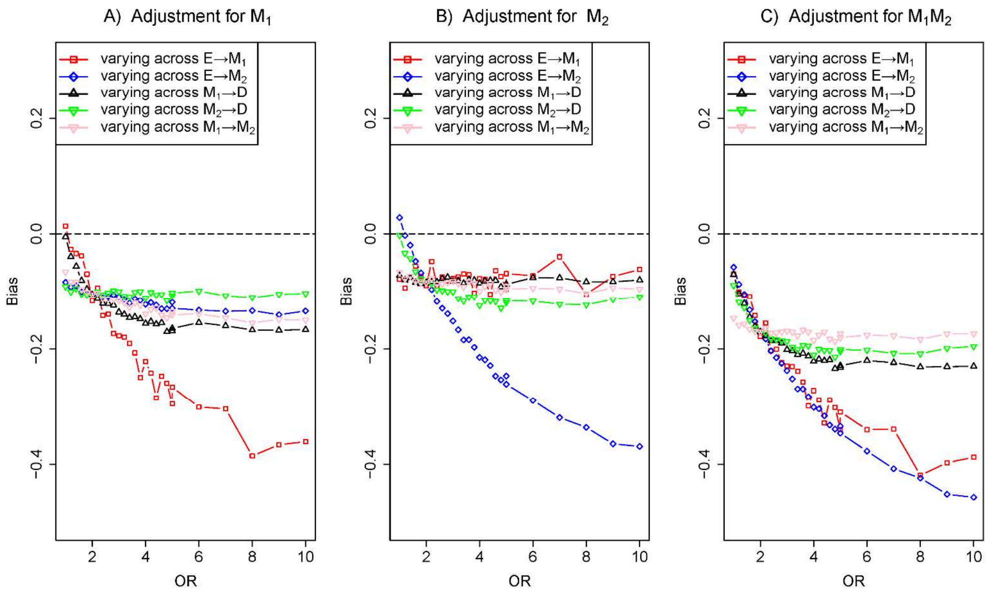

Figure 6: The biases with the effects E→M_1 (red), E→M_2 (blue), M_1 →D (black), M_2 →D (green) and the effect M_2 →M_1 (purple) increasing, respectively.

278x169mm (300 x 300 DPI)

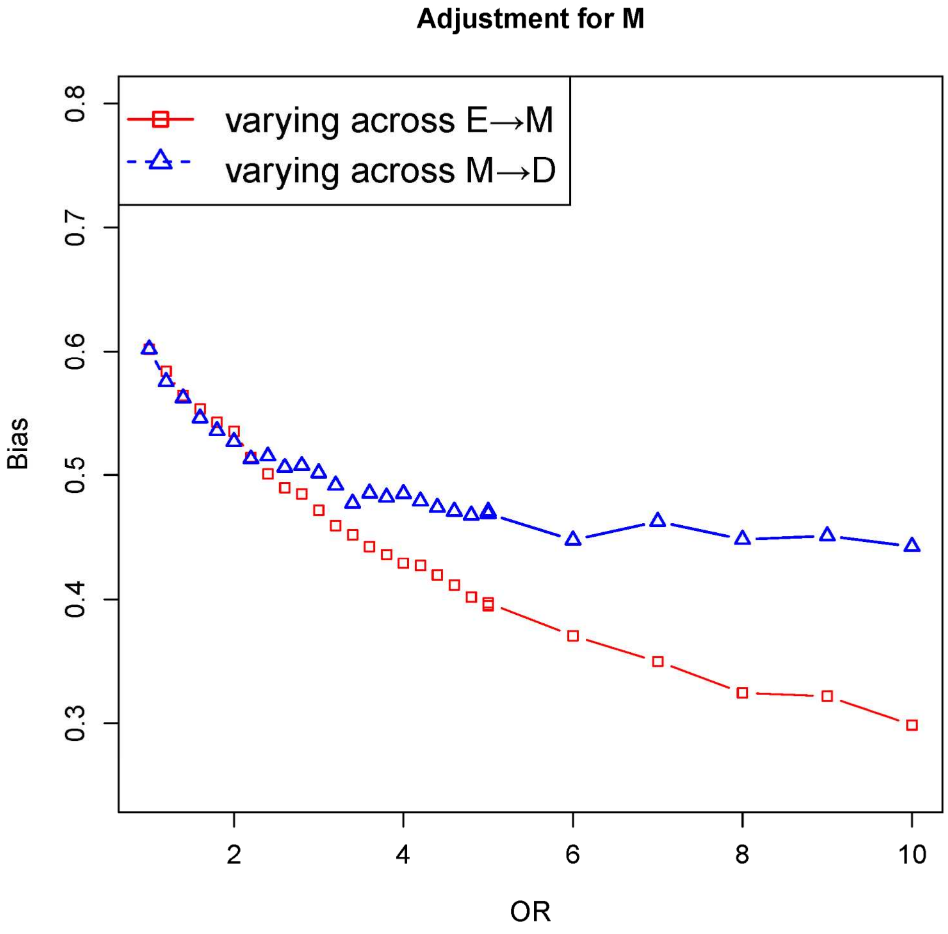

Figure 7: The biases with the effects E→M (red) and M→D (blue) respectively.

177x177mm (300 x 300 DPI)

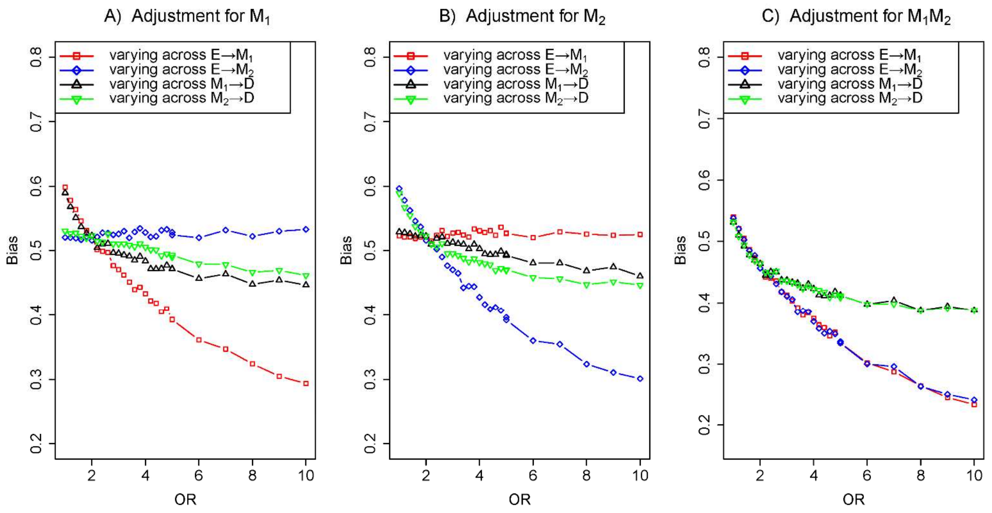

Figure 8 : The biases with the effects E→M_1 (red), E→M_2 (blue), M_1→D (black) and M_2→D (green) respectively.

281x148mm (300 x 300 DPI)

review only

Appendix:

The effect of adjusting for mediator was biased for estimating the total effect of exposure on outcome using logistic regression model. Theoretical derivation of Figure 1a as follow：

Suppose the logistic models among *E*, *M* and *D* are:

$$\text{logit}\{P(D=1|e,m)\}=\alpha_1+\beta_0 e+\beta_2 m,$$

$$\text{logit}\{P(M=1|e)\}=\alpha_0+\beta_1 e.$$

The total effect ($\beta^{TE}_{E\to D}$) of exposure *E* on outcome *D* on the odds ratio ($OR^{TE}_{E\to D}$) scale was equal to

$$\begin{aligned}
\beta^{TE}_{E\to D} &= \log(OR^{TE}_{E\to D}) \\
&= \log\left\{\frac{P(D_e=1)/\{1-P(D_e=1)\}}{P(D_{e*}=1)/\{1-P(D_{e*}=1)\}}\right\} \\
&= \log\left\{\frac{P(D_e=1)\times\{1-P(D_{e*}=1)\}}{\{1-P(D_e=1)\}\times P(D_{e*}=1)}\right\} \\
&= \log\left\{\frac{P(D=1|e=1)\times P(D=0|e*=0)}{P(D=0|e=1)\times P(D=1|e*=0)}\right\} \\
&= \log\left\{\frac{[\sum_m P(D=1|e=1,m)P(m|e=1)]\times[\sum_m P(D=0|e*=0,m)P(m|e*=0)]}{[\sum_m P(D=0|e=1,m)P(m|e=1)]\times[\sum_m P(D=1|e*=0,m)P(m|e*=0)]}\right\}
\end{aligned}$$

The effect ($\beta_{ED|M}(m)$) of adjusting for mediator *M* by logistic regression model is given

$$\begin{aligned}
\beta_{ED|M}(m) &= \text{logit}\{P(D=1|e=1,m)\}-\text{logit}\{P(D=1|e*=0,m)\} \\
&= \log\left\{\frac{P(D=1|e=1,m)\times P(D=0|e*=0,m)}{P(D=0|e=1,m)\times P(D=1|e*=0,m)}\right\} \\
&= \beta_0
\end{aligned}$$

Therefore,

For peer review only - http://bmjopen.bmj.com/site/about/guidelines.xhtml

$$
\begin{aligned}
\text{bias} &= \beta_0 - \log(OR_{E\to D}^{TE}) \\
&= \log\left\{\frac{\exp(\beta_0)}{\exp(\beta_0)\dfrac{\exp(\beta_2)\times A_1+\exp(\beta_2)\times B_1+C_1+D_1}{\exp(\beta_2)\times A_1+B_1+\exp(\beta_2)\times C_1+D_1}}\right\} \\
&= \log\left\{\frac{\exp(\beta_2)\times A_1+B_1+\exp(\beta_2)\times C_1+D_1}{\exp(\beta_2)\times A_1+\exp(\beta_2)\times B_1+C_1+D_1}\right\}
\end{aligned}
$$

where

$$
\begin{aligned}
A_1 &= \exp(\beta_1+\alpha_0)\times(1+\exp(\beta_0+\alpha_1))\times\exp(\alpha_0)\times(1+\exp(\alpha_1)) \\
B_1 &= \exp(\beta_1+\alpha_0)\times(1+\exp(\beta_0+\alpha_1))\times(1+\exp(\beta_2+\alpha_1)) \\
C_1 &= (1+\exp(\beta_0+\beta_2+\alpha_1))\times\exp(\alpha_0)\times(1+\exp(\alpha_1)) \\
D_1 &= (1+\exp(\beta_0+\beta_2+\alpha_1))\times(1+\exp(\beta_2+\alpha_1))
\end{aligned}
$$

Focusing on the difference of between $\exp(\beta_2)\times B_1+C_1$ and $B_1+\exp(\beta_2)\times C_1$.

$$
\begin{aligned}
T(\beta_1) &= \exp(\beta_2)\times B_1+C_1-(B_1+\exp(\beta_2)\times C_1) \\
&= \exp(\beta_2)\times(B_1-C_1)-(B_1-C_1) \\
&= (\exp(\beta_2)-1)\times(B_1-C_1) \\
&= (\exp(\beta_2)-1)\times(\exp(\beta_1+\alpha_0)\times(1+\exp(\beta_0+\alpha_1))\times(1+\exp(\beta_2+\alpha_1)) \\
&\quad -(1+\exp(\beta_0+\beta_2+\alpha_1))\times\exp(\alpha_0)\times(1+\exp(\alpha_1))) \\
&= (\exp(\beta_2)-1)\times\exp(\alpha_0)\times[\exp(\beta_1)\times(1+\exp(\beta_0+\alpha_1)\times(1+\exp(\beta_2+\alpha_1)) \\
&\quad -(1+\exp(\beta_0+\beta_2+\alpha_1))\times(1+\exp(\alpha_1))]
\end{aligned}
$$

Then, detailed dissection:

1: $\beta_2=0$, $bias=0$.

2: $\beta_2>0$,

① $\beta_1=0$: (i) $\beta_0=0$, $bias=0$; (ii) $\beta_0>0$, $bias>0$; (iii) $\beta_0<0$, $bias<0$.

② $\beta_1<0$: (i) $\beta_0=0$, $bias>0$; (ii) $\beta_0>0$, $bias>0$; (iii) $\beta_0<0$, $bias>0$.

proof (iii)

$$
\begin{aligned}
T(\beta_1) &= (\exp(\beta_2)-1)\times\exp(\alpha_0) \\
&\quad \times\{\exp(\beta_1)\times[1+\exp(\beta_0+\alpha_1)+\exp(\beta_2+\alpha_1)+\exp(\beta_0+\beta_2+2\alpha_1)] \\
&\quad -[1+\exp(\beta_0+\beta_2+\alpha_1)+\exp(\alpha_1)+\exp(\beta_0+\beta_2+2\alpha_1)]\}
\end{aligned}
$$

when $\beta_0<0$ and $\beta_2>0 \Rightarrow \exp(\beta_0)-1<0 \quad \exp(\beta_2)-1>0$

According to $(a-1)(b-1)=ab-a-b+1$, when $(a-1)(b-1)<0 \Rightarrow ab+1<a+b$

For peer review only - http://bmjopen.bmj.com/site/about/guidelines.xhtml

$$1+\exp(\beta_0+\beta_2+\alpha_1)+\exp(\alpha_1)+\exp(\beta_0+\beta_2+2\alpha_1)$$
$$<1+\exp(\beta_0+\alpha_1)+\exp(\beta_2+\alpha_1)+\exp(\beta_0+\beta_2+2\alpha_1)$$
$$\Rightarrow \exp(\beta_0+\beta_2)+1<\exp(\beta_0)+\exp(\beta_2)$$

when

$$\beta_1<\log\left\{\frac{\exp(\beta_0+\beta_2)+1}{\exp(\beta_0)+\exp(\beta_2)}\right\}<0$$

$$\beta_1<\log\left\{\frac{1+\exp(\beta_0+\beta_2+\alpha_1)+\exp(\alpha_1)+\exp(\beta_0+\beta_2+2\alpha_1)}{1+\exp(\beta_0+\alpha_1)+\exp(\beta_2+\alpha_1)+\exp(\beta_0+\beta_2+2\alpha_1)}\right\}<0$$

$$\Rightarrow \exp(\beta_1)<\frac{1+\exp(\beta_0+\beta_2+\alpha_1)+\exp(\alpha_1)+\exp(\beta_0+\beta_2+2\alpha_1)}{1+\exp(\beta_0+\alpha_1)+\exp(\beta_2+\alpha_1)+\exp(\beta_0+\beta_2+2\alpha_1)}<1$$

$$\Rightarrow T(\beta_1)=(\exp(\beta_2)-1)\times\exp(\alpha_0)$$
$$\times\{\exp(\beta_1)\times[1+\exp(\beta_0+\alpha_1)+\exp(\beta_2+\alpha_1)+\exp(\beta_0+\beta_2+2\alpha_1)]$$
$$-[1+\exp(\beta_0+\beta_2+\alpha_1)+\exp(\alpha_1)+\exp(\beta_0+\beta_2+2\alpha_1)]\}$$
$$<0$$

Therefore, when $\beta_2>0$, $\beta_1<0$, $\beta_0<0$, then $bias>0$.

③ $\beta_1>0$:(i) $\beta_0=0$, $bias<0$;(ii) $\beta_0<0$, $bias<0$;(iii) $\beta_0>0$, $bias<0$.

proof (iii)

$$T(\beta_1)=(\exp(\beta_2)-1)\times\exp(\alpha_0)$$
$$\times\{\exp(\beta_1)\times[1+\exp(\beta_0+\alpha_1)+\exp(\beta_2+\alpha_1)+\exp(\beta_0+\beta_2+2\alpha_1)]$$
$$-[1+\exp(\beta_0+\beta_2+\alpha_1)+\exp(\alpha_1)+\exp(\beta_0+\beta_2+2\alpha_1)]\}$$

when $\beta_0>0$ and $\beta_2>0 \Rightarrow \exp(\beta_0)-1>0 \quad \exp(\beta_2)-1>0$

According to $(a-1)(b-1)=ab-a-b+1$, when $ab>0\Rightarrow ab+1>a+b$

$$1+\exp(\beta_0+\beta_2+\alpha_1)+\exp(\alpha_1)+\exp(\beta_0+\beta_2+2\alpha_1)$$
$$>1+\exp(\beta_0+\alpha_1)+\exp(\beta_2+\alpha_1)+\exp(\beta_0+\beta_2+2\alpha_1)$$
$$\Rightarrow \exp(\beta_0+\beta_2)+1>\exp(\beta_0)+\exp(\beta_2)$$

when

$$\beta_1>\log\left\{\frac{\exp(\beta_0+\beta_2)+1}{\exp(\beta_0)+\exp(\beta_2)}\right\}>0$$

$$\beta_1>\log\left\{\frac{1+\exp(\beta_0+\beta_2+\alpha_1)+\exp(\alpha_1)+\exp(\beta_0+\beta_2+2\alpha_1)}{1+\exp(\beta_0+\alpha_1)+\exp(\beta_2+\alpha_1)+\exp(\beta_0+\beta_2+2\alpha_1)}\right\}>0$$

$$\Rightarrow \exp(\beta_1)>\frac{1+\exp(\beta_0+\beta_2+\alpha_1)+\exp(\alpha_1)+\exp(\beta_0+\beta_2+2\alpha_1)}{1+\exp(\beta_0+\alpha_1)+\exp(\beta_2+\alpha_1)+\exp(\beta_0+\beta_2+2\alpha_1)}>1$$

For peer review only

For peer review only - http://bmjopen.bmj.com/site/about/guidelines.xhtml

$$\Rightarrow T(\beta_1) = (\exp(\beta_2)-1)\times\exp(\alpha_0)$$
$$\times\{\exp(\beta_1)\times[1+\exp(\beta_0+\alpha_1)+\exp(\beta_2+\alpha_1)+\exp(\beta_0+\beta_2+2\alpha_1)]$$
$$-[1+\exp(\beta_0+\beta_2+\alpha_1)+\exp(\alpha_1)+\exp(\beta_0+\beta_2+2\alpha_1)]\}$$
$$>0$$

Therefore, when $\beta_2>0$, $\beta_1>0$, $\beta_0>0$, then $bias<0$.

3: $\beta_2<0$,

① $\beta_1=0$:(i) $\beta_0=0$, $bias=0$;(ii) $\beta_0>0$, $bias>0$;(iii) $\beta_0<0$, $bias<0$.

② $\beta_1<0$:(i) $\beta_0=0$, $bias<0$;(ii) $\beta_0<0$, $bias<0$;(iii) $\beta_0>0$, $bias<0$.

proof (iii)

$$T(\beta_1) = (\exp(\beta_2)-1)\times\exp(\alpha_0)$$
$$\times\{\exp(\beta_1)\times[1+\exp(\beta_0+\alpha_1)+\exp(\beta_2+\alpha_1)+\exp(\beta_0+\beta_2+2\alpha_1)]$$
$$-[1+\exp(\beta_0+\beta_2+\alpha_1)+\exp(\alpha_1)+\exp(\beta_0+\beta_2+2\alpha_1)]\}$$

when $\beta_0>0$ and $\beta_2<0 \Rightarrow \exp(\beta_0)-1>0 \quad \exp(\beta_2)-1<0$

According to $(a-1)(b-1)=ab-a-b+1$, when $ab<0 \Rightarrow ab+1<a+b$

$$1+\exp(\beta_0+\beta_2+\alpha_1)+\exp(\alpha_1)+\exp(\beta_0+\beta_2+2\alpha_1)$$
$$<1+\exp(\beta_0+\alpha_1)+\exp(\beta_2+\alpha_1)+\exp(\beta_0+\beta_2+2\alpha_1)$$
$$\Rightarrow \exp(\beta_0+\beta_2)+1<\exp(\beta_0)+\exp(\beta_2)$$

when

$$\beta_1<\log\left\{\frac{\exp(\beta_0+\beta_2)+1}{\exp(\beta_0)+\exp(\beta_2)}\right\}<0$$

$$\beta_1<\log\left\{\frac{1+\exp(\beta_0+\beta_2+\alpha_1)+\exp(\alpha_1)+\exp(\beta_0+\beta_2+2\alpha_1)}{1+\exp(\beta_0+\alpha_1)+\exp(\beta_2+\alpha_1)+\exp(\beta_0+\beta_2+2\alpha_1)}\right\}<0$$

$$\Rightarrow \exp(\beta_1)<\frac{1+\exp(\beta_0+\beta_2+\alpha_1)+\exp(\alpha_1)+\exp(\beta_0+\beta_2+2\alpha_1)}{1+\exp(\beta_0+\alpha_1)+\exp(\beta_2+\alpha_1)+\exp(\beta_0+\beta_2+2\alpha_1)}<1$$

$$\Rightarrow T(\beta_1) = (\exp(\beta_2)-1)\times\exp(\alpha_0)$$
$$\times\{\exp(\beta_1)\times[1+\exp(\beta_0+\alpha_1)+\exp(\beta_2+\alpha_1)+\exp(\beta_0+\beta_2+2\alpha_1)]$$
$$-[1+\exp(\beta_0+\beta_2+\alpha_1)+\exp(\alpha_1)+\exp(\beta_0+\beta_2+2\alpha_1)]\}$$
$$>0$$

Therefore, when $\beta_2<0$, $\beta_1<0$, $\beta_0>0$, then $bias<0$.

③ $\beta_1>0$:(i) $\beta_0=0$, $bias>0$;(ii) $\beta_0>0$, $bias>0$;(iii) $\beta_0<0$, $bias>0$.

For peer review only - http://bmjopen.bmj.com/site/about/guidelines.xhtml

proof (iii)

$$T(\beta_1)=(\exp(\beta_2)-1)\times\exp(\alpha_0)$$
$$\times\{\exp(\beta_1)\times[1+\exp(\beta_0+\alpha_1)+\exp(\beta_2+\alpha_1)+\exp(\beta_0+\beta_2+2\alpha_1)]$$
$$-[1+\exp(\beta_0+\beta_2+\alpha_1)+\exp(\alpha_1)+\exp(\beta_0+\beta_2+2\alpha_1)]\}$$

when $\beta_0<0$ and $\beta_2<0\Rightarrow\exp(\beta_0)-1<0\qquad\exp(\beta_2)-1<0$

According to $(a-1)(b-1)=ab-a-b+1$, when $ab>0\Rightarrow ab+1>a+b$

$$1+\exp(\beta_0+\beta_2+\alpha_1)+\exp(\alpha_1)+\exp(\beta_0+\beta_2+2\alpha_1)$$
$$>1+\exp(\beta_0+\alpha_1)+\exp(\beta_2+\alpha_1)+\exp(\beta_0+\beta_2+2\alpha_1)$$
$$\Rightarrow\exp(\beta_0+\beta_2)+1>\exp(\beta_0)+\exp(\beta_2)$$

when

$$\beta_1>\log\left\{\frac{\exp(\beta_0+\beta_2)+1}{\exp(\beta_0)+\exp(\beta_2)}\right\}>0$$

$$\beta_1>\log\left\{\frac{1+\exp(\beta_0+\beta_2+\alpha_1)+\exp(\alpha_1)+\exp(\beta_0+\beta_2+2\alpha_1)}{1+\exp(\beta_0+\alpha_1)+\exp(\beta_2+\alpha_1)+\exp(\beta_0+\beta_2+2\alpha_1)}\right\}>0$$

$$\Rightarrow\exp(\beta_1)>\frac{1+\exp(\beta_0+\beta_2+\alpha_1)+\exp(\alpha_1)+\exp(\beta_0+\beta_2+2\alpha_1)}{1+\exp(\beta_0+\alpha_1)+\exp(\beta_2+\alpha_1)+\exp(\beta_0+\beta_2+2\alpha_1)}>1$$

$$\Rightarrow T(\beta_1)=(\exp(\beta_2)-1)\times\exp(\alpha_0)$$
$$\times\{\exp(\beta_1)\times[1+\exp(\beta_0+\alpha_1)+\exp(\beta_2+\alpha_1)+\exp(\beta_0+\beta_2+2\alpha_1)]$$
$$-[1+\exp(\beta_0+\beta_2+\alpha_1)+\exp(\alpha_1)+\exp(\beta_0+\beta_2+2\alpha_1)]\}$$
$$<0$$

Therefore, when $\beta_2<0$, $\beta_1>0$, $\beta_0<0$, then $bias>0$.

In conclusion:

1: $\beta_2=0$, $bias=0$.

2: $\beta_2\neq0$, $\beta_1=0$:(i) $\beta_0=0$, $bias=0$;(ii) $\beta_0>0$, $bias>0$;(iii) $\beta_0<0$, $bias<0$.

3: (i) $\beta_1\beta_2>0$, $bias<0$. (ii) $\beta_1\beta_2<0$, $bias>0$.

For peer review only - http://bmjopen.bmj.com/site/about/guidelines.xhtml

Supplementary A

The theoretical results of others causal diagrams (Figure 1b-Figure 1f) have been shown in the supplementary of manuscript.

(1) Figure 1(b) is a depiction through two series mediators, decomposing total effects into direct ($E \rightarrow D$) and indirect ($E \rightarrow M_1 \rightarrow M_2 \rightarrow D$) components.

On the odds ratio ($OR_{E\rightarrow D}^{TE}$) scale, the total effect ($\beta_{E\rightarrow D}^{TE} = \log(OR_{E\rightarrow D}^{TE})$), comparing exposure level e with e^*, we could obtain the total effect:

$$\begin{aligned}
\beta_{E\rightarrow D}^{TE} &= \log(OR_{E\rightarrow D}^{TE}) \\
&= \log\left\{\frac{P(D_e=1)/\{1-P(D_e=1)\}}{P(D_{e^*}=1)/\{1-P(D_{e^*}=1)\}}\right\} \\
&= \log\left\{\frac{P(D_e=1)\times\{1-P(D_{e^*}=1)\}}{\{1-P(D_e=1)\}\times P(D_{e^*}=1)}\right\} \\
&= \log\left\{\frac{P(D=1\mid e=1)\times P(D=0\mid e^*=0)}{P(D=0\mid e=1)\times P(D=1\mid e^*=0)}\right\} \\
&= \log\left\{\frac{\xi_1}{\xi_2}\right\}
\end{aligned}$$

$$\xi_1 = [\sum_{m_1m_2} P(D=1\mid e=1,m_2)P(m_2\mid m_1)P(m_1\mid e=1)]\times[\sum_{m_1m_2} P(D=0\mid e^*=0,m_2)P(m_2\mid m_1)P(m_1\mid e^*=0)]$$

$$\xi_2 = [\sum_{m_1m_2} P(D=0\mid e=1,m_2)P(m_2\mid m_1)P(m_1\mid e=1)]\times[\sum_{m_1m_2} P(D=1\mid e^*=0,m_2)P(m_2\mid m_1)P(m_1\mid e^*=0)]$$

The effect ($\beta_{ED|M_1}(m_1)$) of adjusting for mediator M_1 by logistic regression model can be given

$$\begin{aligned}
\beta_{ED|M_1}(m_1) &= \text{logit}\{P(D=1\mid e=1,m_1)\} - \text{logit}\{P(D=1\mid e^*=0,m_1)\} \\
&= \log\left\{\frac{P(D=1\mid e=1,m_1)P(D=0\mid e^*=0,m_1)}{P(D=0\mid e=1,m_1)P(D=1\mid e^*=0,m_1)}\right\} \\
&= \log\left\{\frac{[\sum_{m_2} P(D=1\mid e=1,m_2)P(m_2\mid m_1)]\times[\sum_{m_2} P(D=0\mid e^*=0,m_2)P(m_2\mid m_1)]}{[\sum_{m_2} P(D=0\mid e=1,m_2)P(m_2\mid m_1)]\times[\sum_{m_2} P(D=1\mid e^*=0,m_2)P(m_2\mid m_1)]}\right\}
\end{aligned}$$

The effect ($\beta_{ED|M_2}(m_2)$) of adjusting for mediator M_2 by logistic regression model can be given

$$\begin{aligned}
&\beta_{ED|M_2}(m_2) \\
&= \text{logit}\{P(D=1\mid e=1,m_2)\} - \text{logit}\{P(D=1\mid e^*=0,m_2)\} \\
&= \log\left\{\frac{P(D=1\mid e=1,m_2)P(D=0\mid e^*=0,m_2)}{P(D=0\mid e=1,m_2)P(D=1\mid e^*=0,m_2)}\right\}
\end{aligned}$$

For peer review only

The effect ($\beta_{ED|M_1,M_2}(m_1,m_2)$) of adjusting for mediator M_1 M_2 by logistic regression model can be given

$$\beta_{ED|M_1,M_2}(m_1,m_2) = \text{logit}\{P(D=1|e=1,m_1,m_2)\} - \text{logit}\{P(D=1|e^*=0,m_1,m_2)\}$$
$$= \log\left\{\frac{P(D=1|e=1,m_1,m_2)P(D=0|e^*=0,m_1,m_2)}{P(D=0|e=1,m_1,m_2)P(D=1|e^*=0,m_1,m_2)}\right\}$$
$$= \log\left\{\frac{P(D=1|e=1,m_2)P(D=0|e^*=0,m_2)}{P(D=0|e=1,m_2)P(D=1|e^*=0,m_2)}\right\}$$

Therefore, we could evaluate the biases that contains three adjustment models: A) adjustment for M_1, $bias(m_1) = \beta_{ED|M_1}(m_1) - \beta^{TE}_{E\to D}$; B) adjustment for M_2, $bias(m_2) = \beta_{ED|M_2}(m_2) - \beta^{TE}_{E\to D}$ and C) adjustment for M_1 and M_2, $bias(m_1,m_2) = \beta_{ED|M_1,M_2}(m_1,m_2) - \beta^{TE}_{E\to D}$.

(2) Figure 1c shows that the exposure E independently causes M_1 and M_2 and indirectly influences the outcome D through M_1 and M_2, forming three causal paths $E\to D$, $E\to M_1\to D$ and $E\to M_2\to D$.

On the odds ratio ($OR^{TE}_{E\to D}$) scale, the total effect ($\beta^{TE}_{E\to D} = \log(OR^{TE}_{E\to D})$), comparing exposure level e with e^*, we could obtain the total effect:

$$\beta^{TE}_{E\to D} = \log(OR^{TE}_{E\to D})$$
$$= \log\left\{\frac{P(D_e=1)/\{1-P(D_e=1)\}}{P(D_{e^*}=1)/\{1-P(D_{e^*}=1)\}}\right\}$$
$$= \log\left\{\frac{P(D_e=1)\times\{1-P(D_{e^*}=1)\}}{\{1-P(D_e=1)\}\times P(D_{e^*}=1)}\right\}$$
$$= \log\left\{\frac{P(D=1|e=1)\times P(D=0|e^*=0)}{P(D=0|e=1)\times P(D=1|e^*=0)}\right\}$$
$$= \log\left\{\frac{\xi_1}{\xi_2}\right\}$$

$$\xi_1 = [\sum_{m_1m_2} P(D=1|e=1,m_1,m_2)P(m_2|e=1)P(m_1|e=1)]\times[\sum_{m_1m_2} P(D=0|e^*=0,m_1,m_2)P(m_2|e^*=0)P(m_1|e^*=0)]$$
$$\xi_2 = [\sum_{m_1m_2} P(D=0|e=1,m_1,m_2)P(m_2|e=1)P(m_1|e=1)]\times[\sum_{m_1m_2} P(D=1|e^*=0,m_1,m_2)P(m_2|e^*=0)P(m_1|e^*=0)]$$

The effect ($\beta_{ED|M_1}(m_1)$) of adjusting for mediator M_1 by logistic regression model can be given

$$\beta_{ED|M_1}(m_1) = \text{logit}\{P(D=1|e=1,m_1)\} - \text{logit}\{P(D=1|e^*=0,m_1)\}$$
$$= \log\left\{\frac{P(D=1|e=1,m_1)P(D=0|e^*=0,m_1)}{P(D=0|e=1,m_1)P(D=1|e^*=0,m_1)}\right\}$$
$$= \log\left\{\frac{[\sum_{m_2} P(D=1|e=1,m_1,m_2)P(m_2|e=1)]\times[\sum_{m_2} P(D=0|e^*=0,m_1,m_2)P(m_2|e^*=0)]}{[\sum_{m_2} P(D=0|e=1,m_1,m_2)P(m_2|e=1)]\times[\sum_{m_2} P(D=1|e^*=0,m_1,m_2)P(m_2|e^*=0)]}\right\}$$

The effect ($\beta_{ED|M_2}(m_2)$) of adjusting for mediator M_2 by logistic regression model can be given

$$\beta_{ED|M_2}(m_2) = \text{logit}\{P(D=1|e=1,m_2)\} - \text{logit}\{P(D=1|e^*=0,m_2)\}$$
$$= \log\left\{\frac{P(D=1|e=1,m_2)P(D=0|e^*=0,m_2)}{P(D=0|e=1,m_2)P(D=1|e^*=0,m_2)}\right\}$$
$$= \log\left\{\frac{[\sum_{m_1} P(D=1|e=1,m_1,m_2)P(m_1|e=1)]\times[\sum_{m_1} P(D=0|e^*=0,m_1,m_2)P(m_1|e^*=0)]}{[\sum_{m_1} P(D=0|e=1,m_1,m_2)P(m_1|e=1)]\times[\sum_{m_1} P(D=1|e^*=0,m_1,m_2)P(m_1|e^*=0)]}\right\}$$

The effect ($\beta_{ED|M_1,M_2}(m_1,m_2)$) of adjusting for mediator M_1 M_2 by logistic regression model can be given

$$\beta_{ED|M_1,M_2}(m_1,m_2) = \text{logit}\{P(D=1|e=1,m_1,m_2)\} - \text{logit}\{P(D=1|e^*=0,m_1,m_2)\}$$
$$= \log\left\{\frac{P(D=1|e=1,m_1,m_2)P(D=0|e^*=0,m_1,m_2)}{P(D=0|e=1,m_1,m_2)P(D=1|e^*=0,m_1,m_2)}\right\}$$

Therefore, we could evaluate the biases that contains three adjustment models: A) adjustment for M_1, $bias(m_1) = \beta_{ED|M_1}(m_1) - \beta^{TE}_{E\to D}$; B) adjustment for M_2, $bias(m_2) = \beta_{ED|M_2}(m_2) - \beta^{TE}_{E\to D}$ and C) adjustment for M_1 and M_2, $bias(m_1,m_2) = \beta_{ED|M_1,M_2}(m_1,m_2) - \beta^{TE}_{E\to D}$.

(3) In Figure 1d, there exists five paths from E to D: $E\to D$, $E\to M_1\to D$, $E\to M_2\to D$, $E\to M_1\leftarrow M_2\to D$ and $E\to M_2\to M_1\to D$. In particular, the path $E\to M_1\leftarrow M_2\to D$ is a blocked path, due to the M_1 being a collider node.

On the odds ratio ($OR^{TE}_{E\to D}$) scale, the total effect ($\beta^{TE}_{E\to D} = \log(OR^{TE}_{E\to D})$), comparing exposure level e with e^*, we could obtain the total effect:

For peer review only

$$\begin{aligned}\beta_{E\to D}^{TE} &= \log(OR_{E\to D}^{TE})\\ &= \log\left\{\frac{P(D_e=1)/\{1-P(D_e=1)\}}{P(D_{e*}=1)/\{1-P(D_{e*}=1)\}}\right\}\\ &= \log\left\{\frac{P(D_e=1)\times\{1-P(D_{e*}=1)\}}{\{1-P(D_e=1)\}\times P(D_{e*}=1)}\right\}\\ &= \log\left\{\frac{P(D=1\mid e=1)\times P(D=0\mid e*=0)}{P(D=0\mid e=1)\times P(D=1\mid e*=0)}\right\}\\ &= \log\left\{\frac{\xi_1}{\xi_2}\right\}\end{aligned}$$

$$\begin{aligned}\xi_1 &= [\sum_{m_1 m_2} P(D=1\mid e=1,m_1,m_2)P(m_2\mid e=1)P(m_1\mid e=1,m_2)]\\ &\times[\sum_{m_1 m_2} P(D=0\mid e*=0,m_1,m_2)P(m_2\mid e*=0)P(m_1\mid e*=0,m_2)]\\ \xi_2 &= [\sum_{m_1 m_2} P(D=0\mid e=1,m_1,m_2)P(m_2\mid e=1)P(m_1\mid e=1,m_2)]\\ &\times[\sum_{m_1 m_2} P(D=1\mid e*=0,m_1,m_2)P(m_2\mid e*=0)P(m_1\mid e*=0,m_2)]\end{aligned}$$

The effect ($\beta_{ED|M_1}(m_1)$) of adjusting for mediator M_1 by logistic regression model can be given

$$\begin{aligned}\beta_{ED|M_1}(m_1) &= \text{logit}\{P(D=1\mid e=1,m_1)\}-\text{logit}\{P(D=1\mid e*=0,m_1)\}\\ &= \log\left\{\frac{P(D=1\mid e=1,m_1)P(D=0\mid e*=0,m_1)}{P(D=0\mid e=1,m_1)P(D=1\mid e*=0,m_1)}\right\}\\ &= \log\left\{\frac{[\sum_{m_2} P(D=1\mid e=1,m_1,m_2)P(m_2\mid e=1,m_1)]\times[\sum_{m_2} P(D=0\mid e*=0,m_1,m_2)P(m_2\mid e*=0,m_1)]}{[\sum_{m_2} P(D=0\mid e=1,m_1,m_2)P(m_2\mid e=1,m_1)]\times[\sum_{m_2} P(D=1\mid e*=0,m_1,m_2)P(m_2\mid e*=0,m_1)]}\right\}\\ &= \log\left\{\frac{\xi_1}{\xi_2}\right\}\end{aligned}$$

$$\begin{aligned}\xi_1 &= [\sum_{m_2} P(D=1\mid e=1,m_1,m_2)\frac{P(m_1\mid e=1,m_2)P(m_2\mid e=1)}{\sum_{m_2} P(m_1\mid e=1,m_2)P(m_2\mid e=1)}]\\ &\times[\sum_{m_2} P(D=0\mid e*=0,m_1,m_2)\frac{P(m_1\mid e*=0,m_2)P(m_2\mid e*=0)}{\sum_{m_2} P(m_1\mid e*=0,m_2)P(m_2\mid e*=0)}]\\ \xi_2 &= [\sum_{m_2} P(D=0\mid e=1,m_1,m_2)\frac{P(m_1\mid e=1,m_2)P(m_2\mid e=1)}{\sum_{m_2} P(m_1\mid e=1,m_2)P(m_2\mid e=1)}]\\ &\times[\sum_{m_2} P(D=1\mid e*=0,m_1,m_2)\frac{P(m_1\mid e*=0,m_2)P(m_2\mid e*=0)}{\sum_{m_2} P(m_1\mid e*=0,m_2)P(m_2\mid e*=0)}]\end{aligned}$$

The effect ($\beta_{ED|M_2}(m_2)$) of adjusting for mediator M_2 by logistic regression model can be given

For peer review only

$$\beta_{ED|M_2}(m_2) = \text{logit}\{P(D=1 \mid e=1, m_2)\} - \text{logit}\{P(D=1 \mid e^*=0, m_2)\}$$
$$= \log\left\{\frac{P(D=1 \mid e=1, m_2)P(D=0 \mid e^*=0, m_2)}{P(D=0 \mid e=1, m_2)P(D=1 \mid e^*=0, m_2)}\right\}$$
$$= \log\left\{\frac{[\sum_{m_1} P(D=1 \mid e=1, m_1, m_2)P(m_1 \mid e=1, m_2)] \times [\sum_{m_1} P(D=0 \mid e^*=0, m_1, m_2)P(m_1 \mid e^*=0, m_2)]}{[\sum_{m_1} P(D=0 \mid e=1, m_1, m_2)P(m_1 \mid e=1, m_2)] \times [\sum_{m_1} P(D=1 \mid e^*=0, m_1, m_2)P(m_1 \mid e^*=0, m_2)]}\right\}$$

The effect ($\beta_{ED|M_1,M_2}(m_1, m_2)$) of adjusting for mediator M_1 M_2 by logistic regression model can be given

$$\beta_{ED|M_1,M_2}(m_1, m_2) = \text{logit}\{P(D=1 \mid e=1, m_1, m_2)\} - \text{logit}\{P(D=1 \mid e^*=0, m_1, m_2)\}$$
$$= \log\left\{\frac{P(D=1 \mid e=1, m_1, m_2)P(D=0 \mid e^*=0, m_1, m_2)}{P(D=0 \mid e=1, m_1, m_2)P(D=1 \mid e^*=0, m_1, m_2)}\right\}$$

Therefore, we could evaluate the biases that contains three adjustment models: A) adjustment for M_1, $bias(m_1) = \beta_{ED|M_1}(m_1) - \beta_{E\to D}^{TE}$; B) adjustment for M_2, $bias(m_2) = \beta_{ED|M_2}(m_2) - \beta_{E\to D}^{TE}$ and C) adjustment for M_1 and M_2, $bias(m_1, m_2) = \beta_{ED|M_1,M_2}(m_1, m_2) - \beta_{E\to D}^{TE}$.

(4) In Figure 1e, the causal diagrams contained a confounder of exposure-outcome relationship. On the odds ratio ($OR_{E\to D}^{TE}$) scale, the total effect ($\beta_{E\to D}^{TE} = \log(OR_{E\to D}^{TE})$), comparing exposure level *e* with *e**, we could obtain the total effect:

$$\beta_{E\to D}^{TE} = \log(OR_{E\to D}^{TE})$$
$$= \log\left\{\frac{P(D_e=1)/\{1-P(D_e=1)\}}{P(D_{e^*}=1)/\{1-P(D_{e^*}=1)\}}\right\}$$
$$= \log\left\{\frac{P(D_e=1)\times\{1-P(D_{e^*}=1)\}}{\{1-P(D_e=1)\}\times P(D_{e^*}=1)}\right\}$$
$$= \log\left\{\frac{P(D=1 \mid e=1)\times P(D=0 \mid e^*=0)}{P(D=0 \mid e=1)\times P(D=1 \mid e^*=0)}\right\}$$
$$= \log\left\{\frac{[\sum_{mu} P(D=1 \mid e=1, m, u)P(m \mid e=1)P(u)] \times [\sum_{mu} P(D=0 \mid e^*=0, m, u)P(m \mid e^*=0)P(u)]}{[\sum_{mu} P(D=0 \mid e=1, m, u)P(m \mid e=1)P(u)] \times [\sum_{mu} P(D=1 \mid e^*=0, m, u)P(m \mid e^*=0)P(u)]}\right\}$$

The effect ($\beta_{ED|M}(m)$) of adjusting for mediator M by logistic regression model can be given

For peer review only

$$\beta_{ED|M}(m) = \log it(P(D=1 \mid e=1,m)) - \log it(P(D=1 \mid e^*=0,m))$$

$$= \log\left\{\frac{P(D=1 \mid e=1,m) \times P(D=0 \mid e^*=0,m)}{P(D=0 \mid e=1,m) \times P(D=1 \mid e^*=0,m)}\right\}$$

$$= \log\left\{\frac{[\sum_u P(D=1 \mid e=1,m,u)p(u \mid e=1,m)] \times [\sum_u P(D=0 \mid e^*=0,m,u)p(u \mid e^*=0,m)]}{[\sum_u P(D=0 \mid e=1,m,u)p(u \mid e=1,m)] \times [\sum_u P(D=1 \mid e^*=0,m,u)p(u \mid e^*=0,m)]}\right\}$$

$$= \log\left\{\frac{[\sum_u P(D=1 \mid e=1,m,u)\frac{p(e=1 \mid u)p(u)}{\sum_u p(e=1 \mid u)p(u)}] \times [\sum_u P(D=0 \mid e^*=0,m,u)\frac{p(e^*=0 \mid u)p(u)}{\sum_u p(e^*=0 \mid u)p(u)}]}{[\sum_u P(D=0 \mid e=1,m,u)\frac{p(e=1 \mid u)p(u)}{\sum_u p(e=1 \mid u)p(u)}] \times [\sum_u P(D=1 \mid e^*=0,m,u)\frac{p(e^*=0 \mid u)p(u)}{\sum_u p(e^*=0 \mid u)p(u)}]}\right\}$$

Therefore, we could evaluate the biases of adjustment models: $bias(m) = \beta_{ED|M}(m) - \beta_{E\to D}^{TE}$.

(5) Figure 1f is a depiction of two parallel mediators M_1 and M_2 with confounder. On the odds ratio ($OR_{E\to D}^{TE}$) scale, the total effect ($\beta_{E\to D}^{TE} = \log(OR_{E\to D}^{TE})$), comparing exposure level *e* with *e**, we could obtain the total effect:

$$\beta_{E\to D}^{TE} = \log(OR_{E\to D}^{TE})$$

$$= \log\left\{\frac{P(D_e=1)/\{1-P(D_e=1)\}}{P(D_{e^*}=1)/\{1-P(D_{e^*}=1)\}}\right\}$$

$$= \log\left\{\frac{P(D_e=1) \times \{1-P(D_{e^*}=1)\}}{\{1-P(D_e=1)\} \times P(D_{e^*}=1)}\right\}$$

$$= \log\left\{\frac{P(D=1 \mid e=1) \times P(D=0 \mid e^*=0)}{P(D=0 \mid e=1) \times P(D=1 \mid e^*=0)}\right\}$$

$$= \log\left\{\frac{\xi_1}{\xi_2}\right\}$$

$$\xi_1 = [\sum_{m_1 m_2 u} P(D=1 \mid e=1,m_1,m_2,u)P(m_2 \mid e=1)P(m_1 \mid e=1)P(u)]$$
$$\times [\sum_{m_1 m_2 u} P(D=0 \mid e^*=0,m_1,m_2,u)P(m_2 \mid e^*=0)P(m_1 \mid e^*=0)P(u)]$$

$$\xi_2 = [\sum_{m_1 m_2 u} P(D=0 \mid e=1,m_1,m_2,u)P(m_2 \mid e=1)P(m_1 \mid e=1)P(u)]$$
$$\times [\sum_{m_1 m_2 u} P(D=1 \mid e^*=0,m_1,m_2,u)P(m_2 \mid e^*=0)P(m_1 \mid e^*=0)P(u)]$$

The effect ($\beta_{ED|M_1}(m_1)$) of adjusting for mediator M_1 by logistic regression model can be given

For peer review only

$$\beta_{ED|M_1}(m_1) = \text{logit}\{P(D=1|e=1,m_1)\} - \text{logit}\{P(D=1|e^*=0,m_1)\}$$
$$= \log\left\{\frac{P(D=1|e=1,m_1)P(D=0|e^*=0,m_1)}{P(D=0|e=1,m_1)P(D=1|e^*=0,m_1)}\right\}$$
$$= \log\left\{\frac{\xi_1}{\xi_2}\right\}$$

$$\xi_1 = [\sum_{m_2 u} P(D=1|e=1,m_1,m_2,u)\frac{P(m_2|e=1)P(e=1|u)P(u)}{\sum_u P(e=1|u)P(u)}]$$
$$\times[\sum_{m_2 u} P(D=0|e^*=0,m_1,m_2,u)\frac{P(m_2|e^*=0)P(e^*=0|u)P(u)}{\sum_u P(e^*=0|u)P(u)}]$$
$$\xi_2 = [\sum_{m_2 u} P(D=0|e=1,m_1,m_2,u)\frac{P(m_2|e=1)P(e=1|u)P(u)}{\sum_u P(e=1|u)P(u)}]$$
$$\times[\sum_{m_2 u} P(D=1|e^*=0,m_1,m_2,u)\frac{P(m_2|e^*=0)P(e^*=0|u)P(u)}{\sum_u P(e^*=0|u)P(u)}]$$

The effect ($\beta_{ED|M_2}(m_2)$) of adjusting for mediator M_2 by logistic regression model can be given

$$\beta_{ED|M_2}(m_2) = \text{logit}\{P(D=1|e=1,m_2)\} - \text{logit}\{P(D=1|e^*=0,m_2)\}$$
$$= \log\left\{\frac{P(D=1|e=1,m_2)P(D=0|e^*=0,m_2)}{P(D=0|e=1,m_2)P(D=1|e^*=0,m_2)}\right\}$$
$$= \log\left\{\frac{\xi_1}{\xi_2}\right\}$$

$$\xi_1 = [\sum_{m_1 u} P(D=1|e=1,m_1,m_2,u)\frac{P(m_1|e=1)P(e=1|u)P(u)}{\sum_u P(e=1|u)P(u)}]$$
$$\times[\sum_{m_1 u} P(D=0|e^*=0,m_1,m_2,u)\frac{P(m_1|e^*=0)P(e^*=0|u)P(u)}{\sum_u P(e^*=0|u)P(u)}]$$
$$\xi_2 = [\sum_{m_1 u} P(D=0|e=1,m_1,m_2,u)\frac{P(m_1|e=1)P(e=1|u)P(u)}{\sum_u P(e=1|u)P(u)}]$$
$$\times[\sum_{m_1 u} P(D=1|e^*=0,m_1,m_2,u)\frac{P(m_1|e^*=0)P(e^*=0|u)P(u)}{\sum_u P(e^*=0|u)P(u)}]$$

The effect ($\beta_{ED|M_1,M_2}(m_1,m_2)$) of adjusting for mediator M_1 M_2 by logistic regression model can be given

For peer review only

$$
\begin{aligned}
&\beta_{ED|M_1,M_2}(m_1,m_2) \\
&= \text{logit}\{P(D=1|e=1,m_1,m_2)\} - \text{logit}\{P(D=1|e^*=0,m_1,m_2)\} \\
&= \log\left\{\frac{P(D=1|e=1,m_1,m_2)P(D=0|e^*=0,m_1,m_2)}{P(D=0|e=1,m_1,m_2)P(D=1|e^*=0,m_1,m_2)}\right\} \\
&= \log\left\{\frac{[\sum_u P(D=1|e=1,m_1,m_2,u)\frac{P(e=1|u)P(u)}{\sum_u P(e=1|u)P(u)}]\times[\sum_u P(D=0|e^*=0,m_1,m_2,u)\frac{P(e=1|u)P(u)}{\sum_u P(e=1|u)P(u)}]}{[\sum_u P(D=0|e=1,m_1,m_2,u)\frac{P(e=1|u)P(u)}{\sum_u P(e=1|u)P(u)}]\times[\sum_u P(D=1|e^*=0,m_1,m_2,u)\frac{P(e=1|u)P(u)}{\sum_u P(e=1|u)P(u)}]}\right\}
\end{aligned}
$$

Therefore, we could evaluate the biases that contains three adjustment models: A) adjustment for M_1, $bias(m_1)=\beta_{ED|M_1}(m_1)-\beta^{TE}_{E\to D}$; B) adjustment for M_2, $bias(m_2)=\beta_{ED|M_2}(m_2)-\beta^{TE}_{E\to D}$ and C) adjustment for M_1 and M_2, $bias(m_1,m_2)=\beta_{ED|M_1,M_2}(m_1,m_2)-\beta^{TE}_{E\to D}$.

Supplementary B

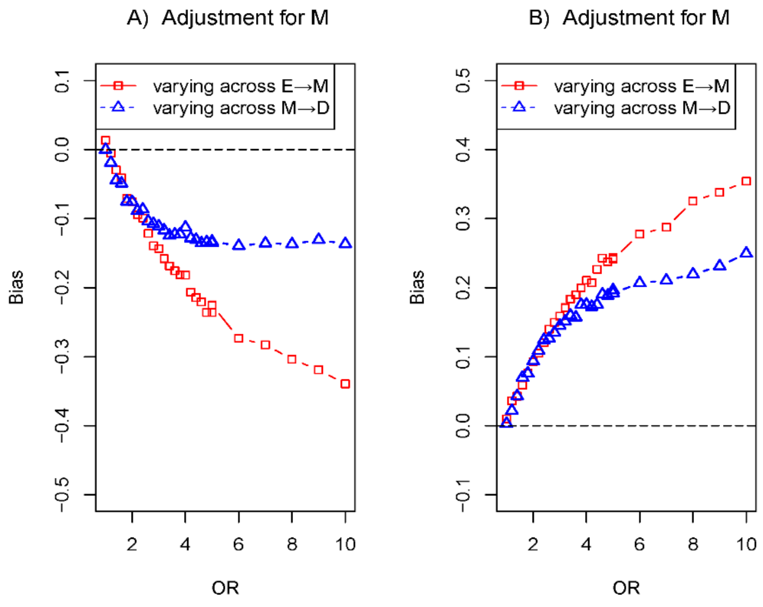

Figure S1: The biases with the effects $E\to M$ (red) and $M\to D$ (blue) increasing, respectively. Comparison of the bias of different effects in adjustment mediator.

The Figure S1-A obtained the result $bias<0$ in Figure 3a with the effects $E\to M$, $M\to D$ and $E\to D$ fixing to ln2. The Figure S1-B gained the result $bias>0$ in Figure 3c with the effects $E\to M$ and $E\to D$ fixing to ln2, effect $M\to D$ fixing to $-\ln 2$. We could obtain the bias performances of varying across the effects of exposure-mediator and mediator-outcome. The effect $E\to M$ of varying across was more sensitive than the effect $M\to D$ of varying across in Figure S1.

For peer review only - http://bmjopen.bmj.com/site/about/guidelines.xhtml

STROBE 2007 (v4) checklist of items to be included in reports of observational studies in epidemiology*
Checklist for cohort, case-control, and cross-sectional studies (combined)

Section/Topic	Item #	Recommendation	Reported on page #
**Title and abstract**	1	(*a*) Indicate the study's design with a commonly used term in the title or the abstract	1
		(*b*) Provide in the abstract an informative and balanced summary of what was done and what was found	2
**Introduction**			
Background/rationale	2	Explain the scientific background and rationale for the investigation being reported	3
Objectives	3	State specific objectives, including any pre-specified hypotheses	3-4
**Methods**			
Study design	4	Present key elements of study design early in the paper	4
Setting	5	Describe the setting, locations, and relevant dates, including periods of recruitment, exposure, follow-up, and data collection	5-6
Participants	6	(*a*) *Cohort study*—Give the eligibility criteria, and the sources and methods of selection of participants. Describe methods of follow-up<br>*Case-control study*—Give the eligibility criteria, and the sources and methods of case ascertainment and control selection. Give the rationale for the choice of cases and controls<br>*Cross-sectional study*—Give the eligibility criteria, and the sources and methods of selection of participants	5-6
		(*b*) *Cohort study*—For matched studies, give matching criteria and number of exposed and unexposed<br>*Case-control study*—For matched studies, give matching criteria and the number of controls per case	
Variables	7	Clearly define all outcomes, exposures, predictors, potential confounders, and effect modifiers. Give diagnostic criteria, if applicable	5-6
Data sources/ measurement	8*	For each variable of interest, give sources of data and details of methods of assessment (measurement). Describe comparability of assessment methods if there is more than one group	*5-6*
Bias	9	Describe any efforts to address potential sources of bias	5-6
Study size	10	Explain how the study size was arrived at	5-6
Quantitative variables	11	Explain how quantitative variables were handled in the analyses. If applicable, describe which groupings were chosen and why	Not applicable
Statistical methods	12	(*a*) Describe all statistical methods, including those used to control for confounding	4-6
		(*b*) Describe any methods used to examine subgroups and interactions	Not applicable
		(*c*) Explain how missing data were addressed	Not applicable
		(*d*) *Cohort study*—If applicable, explain how loss to follow-up was addressed<br>*Case-control study*—If applicable, explain how matching of cases and controls was addressed	Not applicable

For peer review only

		Cross-sectional study—If applicable, describe analytical methods taking account of sampling strategy	
		(*e*) Describe any sensitivity analyses	6
**Results**			
Participants	13*	(a) Report numbers of individuals at each stage of study—eg numbers potentially eligible, examined for eligibility, confirmed eligible, included in the study, completing follow-up, and analysed	Not applicable
		(b) Give reasons for non-participation at each stage	Not applicable
		(c) Consider use of a flow diagram	Not applicable
Descriptive data	14*	(a) Give characteristics of study participants (eg demographic, clinical, social) and information on exposures and potential confounders	7-13
		(b) Indicate number of participants with missing data for each variable of interest	Not applicable
		(c) *Cohort study*—Summarise follow-up time (eg, average and total amount)	Not applicable
Outcome data	15*	*Cohort study*—Report numbers of outcome events or summary measures over time	Not applicable
		Case-control study—Report numbers in each exposure category, or summary measures of exposure	Not applicable
		Cross-sectional study—Report numbers of outcome events or summary measures	7-13
Main results	16	(*a*) Give unadjusted estimates and, if applicable, confounder-adjusted estimates and their precision (eg, 95% confidence interval). Make clear which confounders were adjusted for and why they were included	7-13
		(*b*) Report category boundaries when continuous variables were categorized	Not applicable
		(*c*) If relevant, consider translating estimates of relative risk into absolute risk for a meaningful time period	Not applicable
Other analyses	17	Report other analyses done—eg analyses of subgroups and interactions, and sensitivity analyses	7-13
**Discussion**			
Key results	18	Summarise key results with reference to study objectives	13-15
Limitations	19	Discuss limitations of the study, taking into account sources of potential bias or imprecision. Discuss both direction and magnitude of any potential bias	13-15
Interpretation	20	Give a cautious overall interpretation of results considering objectives, limitations, multiplicity of analyses, results from similar studies, and other relevant evidence	13-15
Generalisability	21	Discuss the generalisability (external validity) of the study results	14
**Other information**			
Funding	22	Give the source of funding and the role of the funders for the present study and, if applicable, for the original study on which the present article is based	16

*Give information separately for cases and controls in case-control studies and, if applicable, for exposed and unexposed groups in cohort and cross-sectional studies.

Note: An Explanation and Elaboration article discusses each checklist item and gives methodological background and published examples of transparent reporting. The STROBE checklist is best used in conjunction with this article (freely available on the Web sites of PLoS Medicine at http://www.plosmedicine.org/, Annals of Internal Medicine at http://www.annals.org/, and Epidemiology at http://www.epidem.com/). Information on the STROBE Initiative is available at www.strobe-statement.org.

For peer review only
